# De novo *EHMT2* variants cause an autosomal dominant *EHMT2*-related Kleefstra syndrome via loss of G9a methyltransferase activity

**DOI:** 10.1038/s41467-026-74987-w

**Published:** 2026-07-01

**Authors:** Aleš Hnízda, Beatriz Martinez-Delgado, Diana Sanchez-Ponce, Javier Alonso, Jeanne Amiel, Tania Attie-Bitach, Ariadna Bada-Navarro, Beatriz Baladron, Eva Bermejo-Sanchez, Vítězslav Brinsa, Ivana Buková, Rosario Cazorla-Calleja, Sylvie Červenková, Shanshan Chow, Petr Dušek, Olha Fedosieieva, Marta Fernandez-Prieto, Sourav Ghosh, Gema Gomez-Mariano, Andrea Gřegořová, Mark James Hamilton, Hana Hartmannová, Esther Hernandez-SanMiguel, Marina Herrero-Matesanz, Kateřina Hodaňová, Alan Kádek, Jennifer Kerkhof, Tjitske Kleefstra, Didier Lacombe, Michael A. Levy, Estrella Lopez-Martin, Ruaud Lyse, Petr Man, Purificacion Marin-Reina, Ellen F. Macnamara, Haley McConkey, Petra Melenovská, Lidia M. Mielu, David Moore, Lenka Steiner Mrázová, Karolína Musilová, Kristýna Neffeová, Petr Nickl, David Pajuelo Reguera, Martina Pavlíková, Lea Pavlovičová, Manuel Posada, Jan Procházka, Kateryna Pysanenko, Sheila Ramos del Saz, Dmitrijs Rots, Jessica Rzasa, Radislav Sedláček, Viktor Stránecký, František Špoutil, Matthew L. Tedder, Louise Thompson, Cynthia J. Tifft, Frederic Tran Mau-Them, Helena Trešlová, Antonio Vitobello, Sarah Hilton, Christopher Campbell, Siddharth Banka, Daniel Jirák, Bekim Sadikovic, Jakub Sikora, Stanislav Kmoch, Maria J. Barrero, Lenka Nosková

**Affiliations:** 1 Research Unit for Rare Diseases, Department of Pediatrics and Inherited Metabolic Disorders, First Faculty of Medicine and General University Hospital, Charles University in Prague, Prague, Czech Republic; 2 Institute of Rare Diseases Research (IIER), Spanish National Institute of Health Carlos III (ISCIII), Madrid, Spain; 3 Undiagnosed diseases program SpainUDP, Madrid, Spain; 4 Centro de Investigación Biomedica en Red de Enfermedades Raras, (CIBERER), Madrid, Spain; 5 Service de Médecine Génomique des Maladies Rares, Hôpital Necker-Enfants Malades, AP-HP, Paris, France; 6 Department of Biochemistry, Faculty of Science, Charles University in Prague, Prague, Czech Republic; 7 Czech Centre for Phenogenomics, Institute of Molecular Genetics, Czech Academy of Sciences, Prague, Czech Republic; 8 Pediatric Neurology Department, Hospital Universitario Puerta de Hierro, Majadahonda, Madrid, Spain; 9 Undiagnosed Diseases Program, National Institutes of Health, Bethesda, MD USA; 10 Department of Neurology and Center of Clinical Neuroscience, First Faculty of Medicine, Charles University and General University Hospital in Prague, Prague, Czech Republic; 11 Verspeeten Clinical Genome Centre, London Health Sciences Centre, London, ON Canada; 12 Institute of Molecular and Clinical Pathology and Medical Genetics, University Hospital Ostrava, Ostrava, Czech Republic; 13 West of Scotland Clinical Genetics Service, Queen Elizabeth University Hospital, Glasgow, UK; 14 Institute of Microbiology, Czech Academy of Sciences, Prague, Czech Republic; 15 Department of Clinical Genetics, Erasmus MC, Rotterdam, The Netherlands; 16 Department of Human Genetics, Radboud University Medical Center, Nijmegen, The Netherlands; 17 Génétique Médicale, CHU Bordeaux, INSERM U1211 (MRGM), Université de Bordeaux, Bordeaux, France; 18 Département de génétique, APHP-Nord, Paris, France, Service de cytogénétique et génétique médicale, Hôpital de la Mère et de l’Enfant, CHU Limoges, Limoges, France; 19 University and Polytechnic Hospital La Fe, Valencia, Spain; 20 Department of Pathology and Laboratory Medicine, Western University, London, ON Canada; 21 South East Scotland Genetic Service, Western General Hospital, Edinburgh, UK; 22 Greenwood Genetic Center, Greenwood, SC USA; 23 Office of the Clinical Director and Medical Genetics Branch, National Human Genome Research Institute, National Institutes of Health, Bethesda, MD USA; 24 Université Bourgogne Europe, CHU Dijon Bourgogne, Laboratoire de Génomique Médicale, FHU-TRANSLAD, Centre de recherche Translationnelle en Médecine moléculaire – Inserm UMR1231 équipe GAD, Dijon, France; 25 Manchester Centre for Genomic Medicine, St Mary’s Hospital, Manchester University NHS Foundation Trust, Health Innovation Manchester, Manchester, UK; 26 Division of Evolution, Infection and Genomics, School of Biological Sciences, Faculty of Biology, Medicine and Health, University of Manchester, Manchester, UK; 27 Institute for Clinical and Experimental Medicine, Prague, Czech Republic; 28 Faculty of Health Studies, Technical University of Liberec, Liberec, Czech Republic; 29 Institute of Pathology, First Faculty of Medicine, Charles University and General University Hospital in Prague, Prague, Czech Republic

**Keywords:** Neurodevelopmental disorders, Disease genetics, Genetics research, Histone post-translational modifications

## Abstract

*EHMT1* and *EHMT2* genes encode human euchromatin histone lysine methyltransferase 1 and 2 (EHMT1 alias GLP; *EHMT2* alias G9a) that form heteromeric GLP/G9a complexes with essential roles in epigenetic regulation of gene expression. While *EHMT1* haploinsufficiency has been established as the cause of Kleefstra syndrome 1, the pathogenesis of G9a dysfunction in human disease remains largely unknown. We identified seven de novo *EHMT2* variants in patients with clinical presentation, episignatures, histone modifications and transcriptomic profiles similar to those of Kleefstra syndrome 1. In vitro studies reveal that these variants encode for structurally stable G9a proteins that are catalytically incompetent due to aberrant interactions either with histone H3 tail or with S-adenosylmethionine. Heterozygous mice carrying a patient-derived variant exhibit growth retardation, facial/skull dysmorphia and aberrant behavior. Here we report pathogenic *EHMT2* variants that likely exert dominant-negative effect on GLP/G9a complexes and thus genocopy the *EHMT1* haploinsufficiency via a distinct molecular mechanism, defining an autosomal dominant *EHMT2*-related Kleefstra syndrome.

## Abstract (web summary)

EHMT1 and EHMT2 regulate chromatin through the GLP/G9a complex. Here, the authors identify pathogenic *EHMT2* variants that impair G9a function, producing Kleefstra-like features and revealing a dominant-negative mechanism underlying *EHMT2*-related neurodevelopmental disease.

## Introduction

Histone methylation regulates chromatin compaction and dynamically orchestrates cellular transcription programs. Histone methylation status is maintained by the activity of histone methyltransferases and demethylases^1^. Their dysregulation has been implicated in many neurodevelopmental disorders, including monogenic and multifactorial diseases (e.g., Alzheimer's or Parkinson's disease)^2^. So far, at least eleven distinct genetic syndromes caused by malfunction of histone methyltransferases have been described^3^.

*EHMT1* and *EHMT2* genes encode human euchromatin histone lysine methyltransferase 1 and 2 (EHMT1 alias GLP; *EHMT2* alias G9a), respectively. GLP and G9a have identical domain architecture with high sequence similarity. Their main characteristic is the presence of a highly conserved SET domain that catalyzes the transfer of a methyl group from S-adenosylmethionine to lysine 9 of histone H3 (H3K9)^4^. The SET domains assemble into functional dimers^5^ and govern the formation of heteromeric GLP/G9a complexes in vivo^6^. Despite the ability of these proteins to form functional homodimers, heterodimers are the preferred form in vivo, displaying the maximum enzymatic activity^6,7^. The proper formation and methylation activity of the GLP/G9a complexes is critical for epigenetic regulation of gene expression in many biological processes including oogenesis^8^, metabolic regulation^9^, or neuronal maturation during brain development^10^.

Haploinsufficiency of *EHMT1* was originally described in the *9q* subtelomeric deletion syndrome, a clinically recognizable neurodevelopmental disorder with congenital anomalies, specific dysmorphic features and other characteristic symptoms known as Kleefstra syndrome 1 (KLEFS1, OMIM# 610253)^11,12^. Over time, additional Kleefstra syndrome 1 patients have been identified with pathogenic frameshift, nonsense or missense *EHMT1* variants, which mostly lead to haploinsufficiency through the instability and decreased amount of functional GLP proteins^13,14^. Recently, a broader phenotypic spectrum of Kleefstra syndrome 1 associated with diverse biochemical properties of differently mutated GLP has been described^15^.

Homozygous loss of either GLP (*Ehmt1*) or G9a (*Ehmt2*) in mice results in embryonic lethality, underscoring the essential role of both proteins in early development^6^. Moreover, heterozygous *Ehmt1*^*+/-*^ mice exhibit a spectrum of neurodevelopmental abnormalities, including facial/skull dysmorphia, behavioral deficits^16^, synaptic impairments and disrupted hippocampal neurogenesis^17^ recapitulating clinical features of Kleefstra syndrome 1^18^. Importantly, in contrast to severe effect observed for *Ehmt1*^*+/-*^, *Ehmt2*^*+/-*^ mice present with only minimal abnormalities^19^.

While the molecular mechanisms of epigenetic dysregulation and pathogenesis involved in the clinical symptoms of Kleefstra syndrome 1 are still under investigation, the epigenetic hallmarks that characterize *EHMT1* haploinsufficiency are well described. Recent research revealed that patients with Kleefstra syndrome 1 show a specific DNA methylation signature (episignature) in peripheral blood^20^. This episignature can aid the diagnosis of patients with clinical symptoms suggestive of Kleefstra syndrome 1, but with no pathogenic *EHMT1* variants revealed by molecular genetic testing and/or with variants of uncertain clinical significance^15,20,21^. Additionally, abnormal epigenetic states have been described in Kleefstra syndrome 1 neurons derived from induced pluripotent stem cells (iPSCs) and in *Ehmt1*^*+/-*^ mice, manifested by dysregulated H3K9me2 global levels^19,22^.

Despite the recognized functional cooperation and interdependency between GLP and G9a^6,23–25^, clinical correlates of G9a dysfunction in humans have not yet been thoroughly described, although the potential involvement of *EHMT2* biallelic loss in a patient with neurodevelopmental delay has been recently suggested^21^.

Here we report clinical, structural, biochemical and molecular correlates of seven de novo heterozygous *EHMT2* variants that we identified through exome/genome sequencing. We show that all these *EHMT2* variants encode for structurally stable but catalytically inactive G9a proteins that seem to possess dominant negative effects on the function of GLP/G9a complexes. Patients harboring these variants showed a clinical phenotype consistent with Kleefstra syndrome 1. Patient-derived cell lines displayed alterations in episignatures, histone modifications and gene expression profiles closely resembling Kleefstra syndrome 1. Notably, the *EHMT2* patients showed a specific episignature in blood, enabling the diagnosis of an additional patient carrying a pathogenic *EHMT2* variant. Moreover, a knock-in mouse model harboring a patient-derived heterozygous deletion of four amino acids in the SET domain of G9a recapitulates multiple key phenotypic features observed in patients with *EHMT2* variants reported here.

## Results

### Multiple de novo variants in *EHMT2* are associated with a neurodevelopmental phenotype and facial dysmorphism

Seven patients (P1-P7) carrying seven different de novo heterozygous variants in *EHMT2* gene have been identified through exome/genome sequencing in several national research or diagnostic programs for rare diseases. The investigators and health care professionals were gathered via GeneMatcher^26^, via Matchmaker Exchange using the RD-Connect Genome-Phenome Analysis Platform (GPAP)^27^ and personal communication. Five of the *EHMT2* variants encode for missense G9a mutations: NM_006709.5: c.3229 G > T p.(Ala1077Ser), c.3229 G > A p.(Ala1077Thr), c.3335 A > G p.(Asn1112Ser), c.3472 T > C p.(Phe1158Leu) and c.3485 A > G p.(Lys1162Arg) and two variants encode for *in-frame* G9a deletions: NM_006709.5: c.3225_3236del p.(Glu1076_Val1079del) and c.3487_3492del p.(Ser1163_Lys1164del). None of these variants have been previously reported in the Genome Aggregation Database (gnomAD v4.1.0) and ClinVar database. The variants encode for evolutionary conserved residues and are predicted as pathogenic by in silico prediction tools (Supplementary Data 1).

**Fig. 1:**
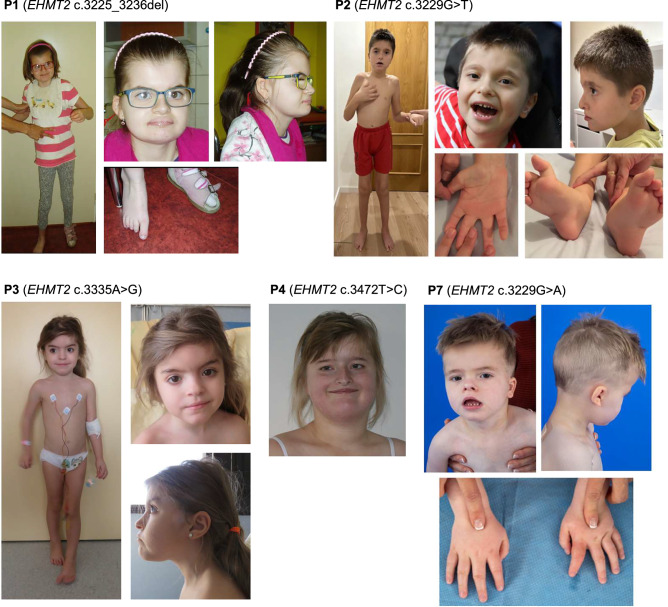
Facial and somatic features of patients with *EHMT2* variants. Photos of probands P1–P4 and P7 show facial dysmorphism including midface hypoplasia, synophrys, hypertelorism, dysplastic ears, short philtrum, and deeply set eyes. Long hallux is visible in P1–P3. Genotypes corresponding to the *EHMT2* transcript NM_006709.5 are listed for each patient.

The patients‘ cohort was composed of four females and three males aged 2 – 16 years. All patients presented with facial dysmorphism including midface hypoplasia, synophrys, hypertelorism, dysplastic ears, short philtrum and deeply set eyes (Fig. 1), and cardiovascular anomalies including septal defects (ventricular or atrial), pulmonal valve stenosis and coarctation of the aorta. All but one patient presented with hypotonia, psychomotor and developmental delay, absence of speech and behavioral abnormalities. A single patient (P3) with the c.3335 A > G p.(Asn1112Ser) variant had normal psychomotor, cognitive and speech abilities but exhibited paroxysmal hyperkinetic (choreoathetoid) movements. Individually, patients presented with various other abnormalities including pes planovalgus or equinovalgus, postaxial polydactyly or big halluxes, kidney cysts and other urogenital abnormalities. Detailed clinical description of patients is provided in Table 1, Supplementary Data 1 and in Supplementary Information. Overall, the clinical presentation of the patients was very similar to those of Kleefstra syndrome 1 caused by *EHMT1* haploinsufficiency.

**Table 1:** Summary of key clinical features in patients (see Supplementary Data 1 for full details)

Clinical data of patients with EHMT2 variants							
Study Patient ID	**P1**	**P2**	**P3**	**P4**	**P5**	**P6**	**P7**
Inheritance	de novo	de novo	de novo	de novo	de novo	de novo	de novo
Zygosity	heterozygous	heterozygous	heterozygous	heterozygous	heterozygous	heterozygous	heterozygous
Mutation (NM_006709.5)	c.3225_3236del	c.3229G>T	c.3335A>G	c.3472T>C	c.3487_3492del	c.3485A>G	c.3229G>A
Mutation (NP_006700.3)	p.Glu1076_Val1079del	p.Ala1077Ser	p.Asn1112Ser	p.Phe1158Leu	p.Ser1163_Lys1164del	p.Lys1162Arg	p.Ala1077Thr
Gender	female	male	female	female	male	female	male
**Neurological findings:**							
Hypotonia	yes	yes	no	yes	yes	yes	yes
Seizures	no	one episode	no	no	febrile seizure at 20 months	no	febrile seizures at 22 months
**Psycho-developmental and behavioral findings:**							
Development	severe developmental delay	psychomotor delay	normal	moderate developmental delay	motor delay	global developmental delay	psychomotor delay
Behavior	hyperkinetic movements	stereotypic hand movements	hyperkinetic and choreoathetotic movements	behavioral disorder	NA	stereotypic movements	atypical hand movements
Verbal abilities	absent speech	no speech	complex sentences	simple sentences	a few words	absent speech	no speech
**Cardiologic findings:**	stenosis of the arteria pulmonalis	septal defect, coarctation of the aorta	cardiac anomalies	atrial septal defect	ventricular septal defect	coarctation of the aortic isthmus	patent foramen ovale
**Other:**	growth and sexual maturation disorder	kidney cysts	kidney anomalies	no	stenosis of one lacrimal duct	livedo reticularis	cryptorchidism at birth

### Episignatures of patients with *EHMT2* variants overlap with those of Kleefstra syndrome 1 yet reveal a specific hypomethylated episignature

Our initial discovery cohort consisted of patients P1 to P6. Analyses of patients’ blood DNA methylation profiles using the Episign assay^20,28–30^ revealed an episignature profile that matched the Kleefstra syndrome 1 episignature (Supplementary Fig. 1). Unsupervised hierarchical clustering and multidimensional scaling (MDS) plots demonstrated that the patients with *EHMT2* variants clustered closely with the Kleefstra syndrome 1 cohort, with one sample (P3) showing complete overlap (Supplementary Fig. 1a, b). Furthermore, a multi-class supervised classification model predicted the *EHMT2* patients as Kleefstra syndrome 1 with high confidence, while effectively distinguishing them from other established episignatures associated with genetic disorders in the EpiSign™ Knowledge Database (EKD) (Supplementary Fig. 1c).

**Fig. 2:**
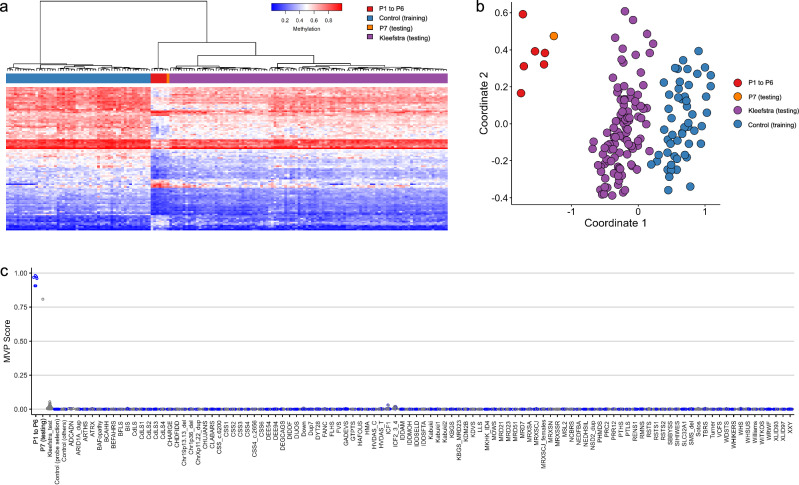
EpiSign (DNA methylation) analysis of peripheral blood from patients with *EHMT2* variants. Unsupervised clustering of the discovery cohort and assessment of *EHMT2* variants using the identified methylation signature, visualized via (**a**) heatmap and (**b**) multidimensional scaling (MDS) plot. Discovery samples from patients with *EHMT2* variants are shown in red, matched controls in blue, *EHMT2* test samples in orange, and Kleefstra (*EHMT1*) test samples in purple. **c** Methylation Variant Pathogenicity (MVP) scores from the SVM classifier for EpiSign™ Knowledge Database, *EHMT2*, and Kleefstra test samples, averaged over four-fold cross-validation (gray). Higher MVP scores indicate greater similarity to the discovery case methylation profile (blue).

Next, we conducted a discovery analysis to assess the presence of an *EHMT2*-specific episignature. For that, we compared the episignatures of the six patients carrying the *EHMT2* variants and 54 matched controls, and identified 244 top differentially methylated probes. The mean methylation difference across the probes between patients and controls ranged from +45.52% to −35.77% (median = −18.60%), with 88.52% of the signature probes being hypomethylated (Supplementary Data 2, Supplementary Fig. 2a). Interestingly, these probes were mapped to 155 genes that were enriched in the Gene Ontology term multicellular organism development at a *p*-value: 6.1E-7. Cluster analysis validated the feature selection, showing a clear separation between *EHMT2* patients and controls. The analysis also showed a clear separation from the testing Kleefstra syndrome 1 samples in both heatmap and multidimensional scaling (MDS) plots (Fig. 2a, b). Furthermore, an unsupervised leave-one-out cross-validation on the discovery cases demonstrated that each hold-out test case consistently clustered with the remaining discovery cases in heatmaps and aligned closely with patient group centroids in MDS plots (Supplementary Fig. 2b). This supports the reproducibility and robustness of the identified episignature. We then developed a Support Vector Machine (SVM)-based model to evaluate its diagnostic utility for *EHMT2*-related disorders. The model demonstrated high specificity, as nearly all EKD test samples yielded methylation variant pathogenicity (MVP) scores close to 0 (Fig. 2c). It also showed sensitivity to an additional patient (P7) with a de novo *EHMT2* variant (*EHMT2*(NM_006709.5):c.3229 G > A p.(Ala1077Thr) that was identified in the EKD database after the discovery cohort. This patient had an MVP score greater than 0.75, clustered with discovery cases in the MDS plot, and displayed similar profiles in the heatmap (Fig. 2a–c). The clinical characteristics of this patient were consistent with those of the *EHMT2* patient cohort (Table 1). Additionally, all testing Kleefstra samples had very low MVP scores ( < 0.1), did not match the signature, and exhibited profiles more similar to controls. Overall, the episignature analysis suggest that *EHMT2*-related disorders are related to Kleefstra syndrome 1 but represent a distinct molecular entity with its own specific episignature.

### *EHMT2* patients show histone modification and gene expression signatures that match those of Kleefstra syndrome 1

**Fig. 3:**
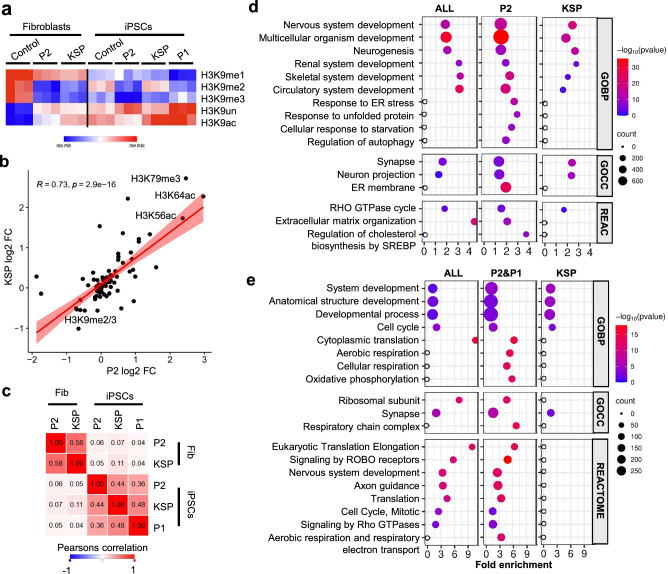
Epigenetic and transcriptomic analyses of cell lines derived from a healthy control, a Kleefstra Syndrome 1 patient (KSP) and patients P1 and P2 carrying *EHMT2* variants. **a** Relative abundance of the indicated histone H3K9 modifications quantified by mass spectrometry in fibroblasts cultures (Fib) and iPSCs in triplicates. **b** Correlation of changes in 92 histone modification states detected by mass spectrometry between KSP or P2 and control fibroblast. Pearson correlation coefficient (R) and two-sided *P*-value are shown. The red line represents the linear regression fit, and the shaded area indicates the 95% confidence interval. **c** Heatmap of Pearson's correlation coefficients of gene expression changes between the indicated cell lines. **d** DAVID enrichments of Gene Ontology Biological Processes (GOBP), Gene Ontology Cellular Compartments (GOCC), and Reactome (REAC) terms in genes commonly upregulated in KSP and P2 fibroblasts (ALL), upregulated in P2 fibroblasts only (P2) or KSP fibroblasts only (KSP). *P*-values correspond to DAVID’s modified one-tailed Fisher’s exact test (EASE score). **e** DAVID enrichments of GOBP, GOCC, and Reactome terms in genes commonly upregulated in P1, P2, and KSP iPSCs (ALL), upregulated in P2 and P1 (P1&P2) iPSCs only or KSP iPSCs only (KSP). *P*-values correspond to DAVID’s modified one-tailed Fisher’s exact test (EASE score). Hollow circles indicate not significant at *p*-value > 0.05.

Next, we investigated whether the identified patients had a histone modification profile similar to the one reported for Kleefstra syndrome 1 and in accordance with the loss of G9a activity. We established fibroblast cultures from skin biopsy from patient P2, together with a healthy skin donor and a Kleefstra syndrome 1 patient (KSP) carrying a frameshift variant in *EHMT1* (NM_024757.5: c.1881delT p.(His629ThrfsTer12)). We further generated iPSCs from the established fibroblasts cultures and from peripheral blood mononuclear cells available from patient P1 that were characterized according to the established standards^31,32^ (Supplementary Figs. 3 and 4). Quantification of histone modifications in fibroblasts and iPSCs lines by mass spectrometry revealed significantly (*p*-value < 0.05) decreased levels of H3K9me1 or H3K9me2 in all patients’ cell lines compared to control cell lines (Fig. 3a, Supplementary Data 3). While changes in histone modifications in iPSCs were rather subtle, more pronounced changes were observed in fibroblast cultures in which we detected significant downregulation of H3K9me1/2/3 and upregulation of unmodified and acetylated H3K9 in patients. The changes in histone modifications were consistent between Kleefstra syndrome 1 and P2 patient’s fibroblasts, showing coordinated upregulation of several histone marks involved in gene activation, such as H3K56ac, H3K64ac and H3K79me3 (Fig. 3b and Supplementary Fig. 5a).

In agreement with the altered histone modification patterns in the fibroblasts and iPSCs patient-derived cell lines, we observed significant changes in gene expression in patients compared to controls that correlated among patients (Fig. 3c and Supplementary Fig. 6a–c). Accordingly, there was a significant overlap between the patients’ cell lines in up- and down-regulated genes (Supplementary Fig. 6d–g). Upregulated genes, most likely G9a direct target genes^33–35^, were enriched in alternative lineages, such as skeletal, muscle and neuronal systems development, and in cell cycle and Rho GTPase pathways. In addition, we identified genes exclusively upregulated in *EHMT2* patients’ cell lines. Specifically, we observed a stress-related signature characterized by endoplasmic reticulum stress and unfolded protein response, accompanied by autophagy and increased cholesterol biosynthesis in fibroblasts and upregulated oxidative phosphorylation and aerobic respiration in iPSCs. None of these signatures were enriched among genes upregulated only in KSP cells or commonly upregulated in *EHMT2* patients’ cell lines and KSP cell lines. (Fig. 3d, e, Supplementary Figs. 7 and 8). Overall, the histone modification and gene expression changes observed in patient-derived cells carrying *EHMT2* variants largely overlap with those reported in Kleefstra syndrome 1, while also showing distinct differences, consistent with impaired H3K9 methyltransferase activity.

### *EHMT2* variants have no effect on GLP and G9a transcript and protein levels

To explore the effects of the identified variants, we examined *EHMT1/2* transcripts and GLP/G9a protein amounts in the available patient-derived samples. RNA sequencing revealed very similar amounts of wild-type and mutant *EHMT2* transcripts in peripheral leukocytes of P1, P2 and P3 and similar transcript levels of EHMT1 and *EHMT2* in peripheral leukocytes obtained from P1 to P3, iPSCs from P1 and P2, and fibroblasts from P2 (Supplementary Fig. 9a, b). The amounts of GLP and G9a protein were also very similar in patient-derived and control iPSCs as determined by western blot (Supplementary Fig. 9c). Next, we immunoprecipitated G9a from iPSCs and analyzed co-precipitating proteins by western-blotting and mass spectrometry (Supplementary Fig. 9d and Supplementary Data 4). We found that GLP, along with other known G9a interactors, such as WIZ and ZNF462^36^ was efficiently co-immunoprecipitated in both control and patients’ cell lines. Overall, the identified *EHMT2* variants do not affect the formation of sufficient amounts of a structurally stable GLP-G9a complex.

### *EHMT2* variants cluster at the G9a enzyme-substrate interface

**Fig. 4:**
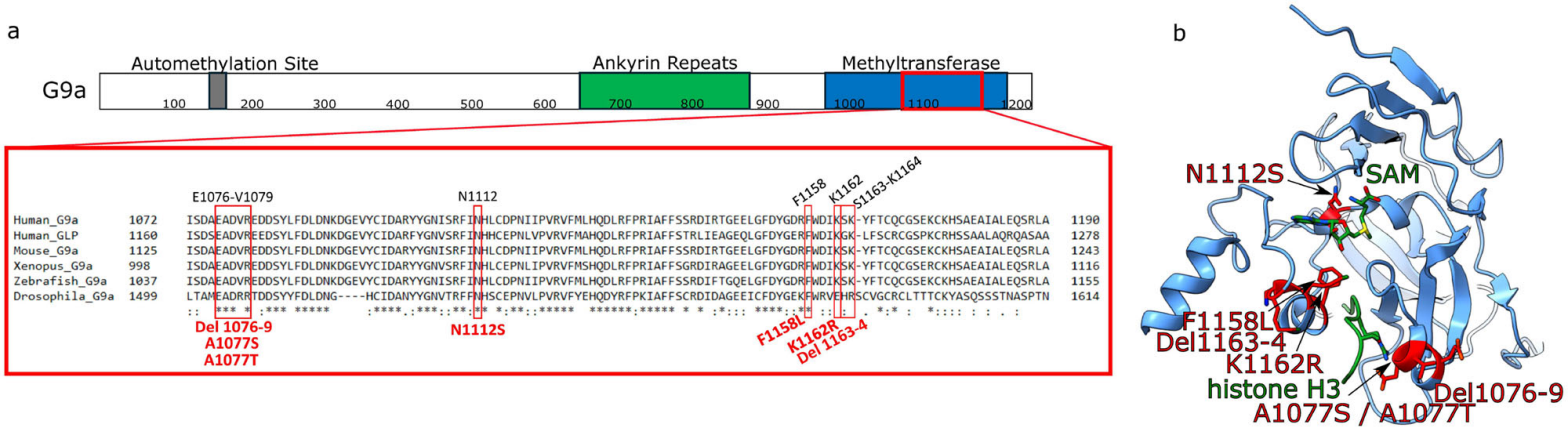
Sequence and structural positioning of mutations in the G9a protein. **a** Aminoacid sequence alignment shows highly conserved residues in the catalytic pocket of the methyltransferase domain. Positions of the mutations are highlighted by red squares. **b** Structural mapping demonstrates localization of the mutations in binding pockets for histone H3 tail and SAM in the G9a catalytic site. Mapping was performed using crystal structure of SET-domain of G9a in complex with SAM and histone H3 peptide (PDB ID 5JIN). The variant amino acid residues are highlighted as red sticks; G9a substrates - histone H3 and SAM - are depicted in green color.

Sequence alignment and structural modeling (Fig. 4, Supplementary Fig. 10 and Supplementary Table 1) revealed that six of the predicted amino acid changes (Del 1076-9, A1077S, A1077T, F1158L, K1162R, and Del 1163-4) affect the G9a binding site for histone H3 tail, whereas the N1112S substitution affects the G9a binding site for the methyl group donor (S-adenosylmethionine). Thus, all identified variants cluster at the G9a enzyme-substrate interface with potential effects on the histone H3 tail interaction and catalytic activity.

### All *EHMT2* variants compromise the catalytic activity of G9a through defective enzyme-substrate interactions

**Fig. 5:**
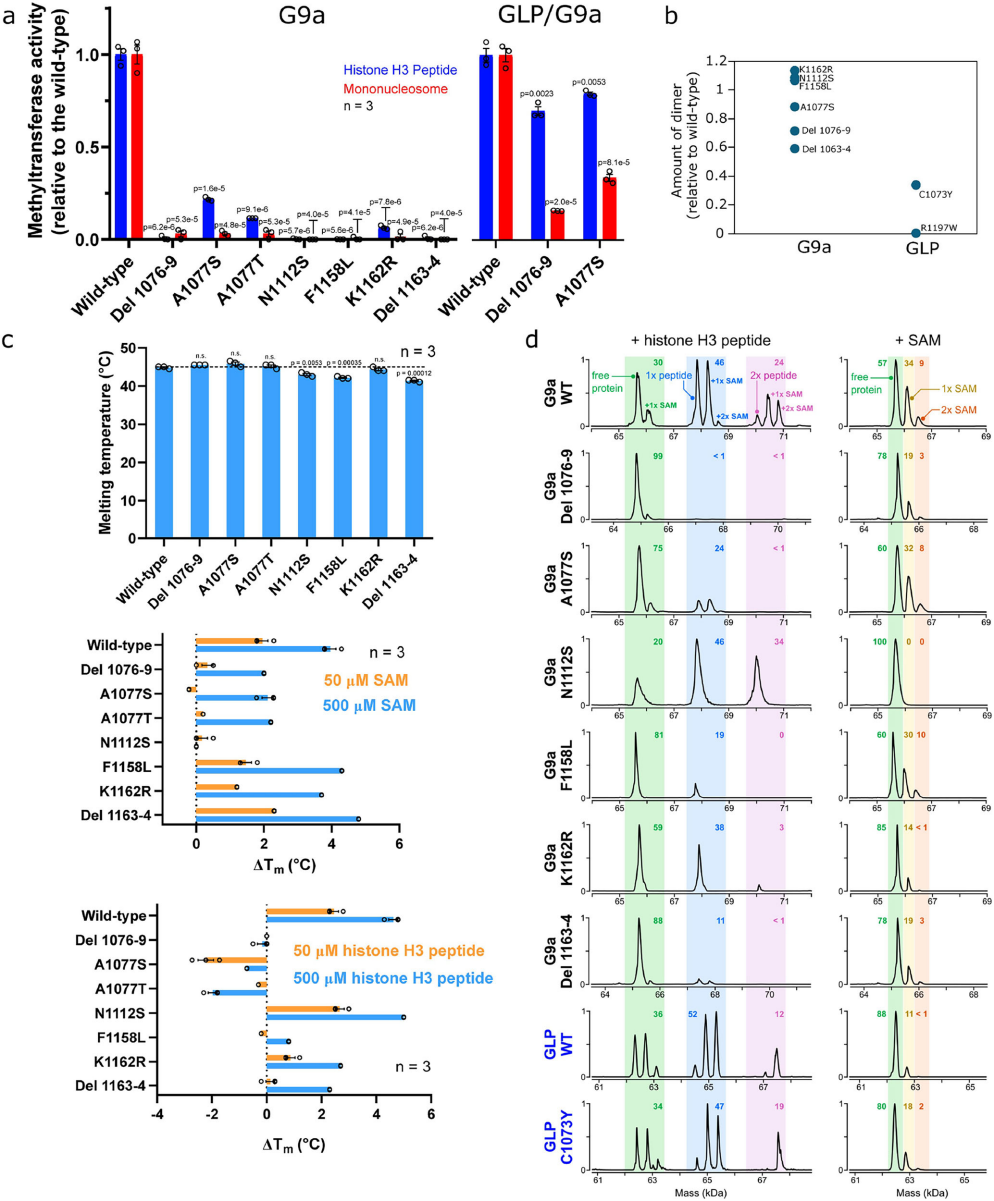
Functional characteristics of G9a variants assessed with recombinant proteins. **a** Methylation activity of the G9a mutants with histone H3 peptide (blue) and mononucleosome (red) as substrates was studied by quantitative bioluminescence-based assay (Mtase-Glo by Promega). The activity was assessed for G9a homodimers (left) and GLP-G9a heterodimers (right). The values represent the means of relative activity to the wild-type enzyme with standard errors from three measurements. The *p*-values indicate variants with significant differences from the wild-type. Whereas “n.s.“means changes were not significant, as determined by a two-tailed Student’s t-test (*p* < 0.01). The symbol “#” indicates “activity not detected”. **b** Quantitative assessment for dimer formation of G9a and GLP mutants using native mass spectrometry (nESI-MS). Points represent relative amounts of dimers related to the wild-type proteins. Dimerization is severely affected in GLP but not in G9a mutants. **c** Thermostability of the G9a mutants in their free and substrate-bound states studied by differential scanning fluorimetry in at least in triplicates. The upper panel shows the melting temperatures of free G9a proteins are represented as mean values with standard errors. The dashed line indicates the melting temperature of the wild-type protein. The *p*-values indicate variants with significantly different changes from the wild-type. Whereas “n.s.“ means changes were not significant, as determined by a two-tailed Student’s t-test (*p* < 0.01). The two lower panels show the stabilization of G9a proteins upon the addition of individual substrates (SAM or histone H3 peptide), as shown by differences in melting temperature in comparison to the free protein. The values represent the means with standard errors from three measurements. **d** Binding affinity of the G9a variants towards individual substrates was assessed by native mass spectrometry. The protein and substrate concentrations were maintained at 5 µM. Representative deconvoluted nMS spectra revealed specific mass shifts that corresponded to G9a complexes with SAM ( + 398 Da) or histone H3 peptide ( + 2100 Da) bound, as shown by highlighted areas with relative abundance.

Since structural modeling showed that all mutations cluster at the G9a enzyme-substrate interface, we cloned, expressed, and purified recombinant methyltransferase domains and examined their catalytic activities. Specifically, we determined the extent of methylation of K9 in an N-terminal histone H3 peptide (amino acid residues 1–20) and a nucleosome. Initially, using semi-quantitative analysis by MALDI-TOF mass spectrometry we observed that mono- and di-methylation of histone H3 peptide was decreased with A1077S, A1077T, K1162R and Del 1163-4 mutations and absent in Del 1076-9, N1112S and F1158L mutations (Supplementary Fig. 11). Subsequently, we used a bioluminescence-based assay analysis to quantify the initial velocity rates of lysine methylation on histone H3 peptide or nucleosome. We observed that six of the mutations, Del 1076-9, A1077T, N1112S, F1158L, and K1162R, Del 1163-4, decreased the methylation activity towards histone H3 peptide to less than 10% of the wild-type enzyme (Fig. 5a). A higher ( ~ 20%) residual activity was observed for the A1077S mutation. Notably, the methylation of mononucleosome was reduced to less than 5% of the wild-type enzyme activity with all studied mutations, including the A1077S. These data demonstrate that all studied mutations reduce the catalytic competence of G9a toward the histone H3 tail.

Next, we examined the impact of two G9a variants with compromised catalytic activity (Del 1076-9 and A1077S) in the context of the GLP-G9a heterodimer. In the GLP-G9a heterodimer, ankyrin repeat domains are required for efficient methylation with nucleosomal substrates^7^. Therefore, we co-expressed GLP and G9a constructs comprising the ankyrin repeat region and the methyltransferase domain and purified the formed heterodimeric complexes. A bioluminescence-based methylation assay revealed only a modest reduction in activity of the G9a mutant heterodimers with histone H3 peptide ( ≈ 70 % of wild-type), whereas activity with nucleosomes was markedly diminished ( < 35 % of wild-type) (Fig. 5a). These data indicate that the G9a variants substantially impair nucleosomal methylation activity of the GLP-G9a heterodimer and that heterodimer-associated GLP cannot compensate for catalytically deficient G9a mutants.

To identify the molecular mechanisms of this reduced catalytic competence, we assessed the structural stability and binding affinity of the recombinant G9a mutants towards their substrates. First, we used native mass spectrometry (nMS), which measures the mass of intact non-covalent protein complexes^37^, to quantify the relative amounts of properly folded dimeric forms of the corresponding mutated proteins. Native MS revealed that all G9a mutants predominantly assemble into compactly folded dimers (Fig. 5b and Supplementary Fig. 12a) and that GLP-G9a heterodimer formation is favored upon co-expression in all protein variants (Supplementary Fig. 12b). Using differential scanning fluorimetry (DSF) performed under temperature gradient, we determined that the melting temperatures of free homodimeric G9a proteins were slightly reduced by N1112S, F1158L, and Del1163-4 mutations and not affected by Del 1076-9, A1077S, A1077T, and K1162R mutations (Fig. 5c). These data showed that the variants do not have major destabilizing effects on the overall G9a structure and its dimerization.

Next, we determined the binding affinities of individual G9a mutations for histone H3 peptide and SAM using nMS. Binding affinities were estimated from observed substrate binding visible as 398 Da (for SAM) or 2100 Da (for histone H3 peptide) mass increases of corresponding non-covalent protein-ligand complexes. This analysis revealed that mutants Del 1076-9, A1077S, F1158L, K1162R, and Del 1163-4 had reduced binding to histone H3 peptide but unaffected binding to SAM (Fig. 5d). Opposite results were observed for the N1112S mutation, which exhibited normal binding to histone H3 peptide but failed to interact with SAM. Substrate binding behavior of individual G9a mutations was further corroborated by binding affinity estimated from substrate concentration-dependent increased thermostability of corresponding protein-ligand complexes using DSF. This analysis further confirmed the decreased binding capacity of Del 1076-9, A1077S, F1158L, K1162R, and Del 1163-4 mutations to histone H3 peptide and revealed that the Del 1076-9 and A1077S mutations had partially reduced binding capacities to SAM (Fig. 5c). Consistently with our observations above, opposite results were recorded for the N1112S mutation that exhibited normal binding to histone H3 peptide but failed to interact with SAM.

Together, nMS and DSF revealed that pathogenic G9a mutations disturb the catalytic competence of the enzyme through defective interaction with one or both of the enzyme substrates, while the structural integrity and stability of the protein are not affected.

Using the established protocol, we also examined the heterozygous G9a variant p.T961I previously reported as a variant of unknown significance in a patient with an autistic disorder and no phenotypic overlap with Kleefstra syndrome 1^38^. This variant, located outside the methyltransferase site, exhibited normal methyltransferase activity and protein stability (Supplementary Fig. 13). Thus, its biochemical profile differs from that of the pathogenic variants identified in this study. Given these results, we classify this variant as likely benign.

### Pathogenic EHMT1 SET domain variants affect protein stability and dimer formation

To compare the *EHMT2* variants with previously identified pathogenic missense *EHMT1* variants, we assessed the effects of two previously described SET-domain GLP pathogenic mutants: C1073Y and R1197W^13^.

Our biochemical examinations revealed that both mutations render the GLP SET-domain considerably unstable when expressed in *E.coli*, with most of the mutant proteins accumulating in insoluble fractions, which either entirely abolished (R1197W) or decreased (C1073Y) purification yields (Supplementary Fig. 14a, b). With reduced but sufficient amounts of purified C1073Y mutant, we were able to determine its decreased thermal stability (Supplementary Table 2) and impaired dimerization (Fig. 5b, d). Despite this, the C1073Y mutant showed binding affinities to histone H3 peptide and SAM close to those of wild-type GLP (Fig. 5d) and had a relatively high ( ~ 60%) methylation activity with histone H3 peptide but almost no ( < 1%) activity with mononucleosome compared to the wild-type protein (Supplementary Fig. 14c).

To further examine the aberrant catalysis of mutated methyltransferases, we compared the kinetic parameters of G9a (A1077S) and GLP (C1073Y) variants retaining relatively high residual activity toward histone H3 peptide. This analysis revealed that the C1073Y GLP mutation did not significantly affect enzyme turnover number (k_cat_), while the A1077S G9a mutation significantly decreased it (Supplementary Table 3). Specifically, k_cat_ values for A1077S G9a were 2.2-fold and 4.5-fold lower toward histone H3 peptide and SAM, respectively, compared to the wild-type enzyme. Therefore, our biochemical and enzymatic analyses confirm that pathogenic G9a variants are catalytically incompetent per se, while the tested pathogenic missense GLP mutations lose their activities due to structural instability.

### *Ehmt2*^+/del_1076-1079^ mice present with postnatal developmental abnormalities, craniofacial dysmorphia, and gait alterations

To further validate the functional relevance of the *EHMT2* variants at the organismal level, we generated a mouse *knock-in* model (*Ehmt2*^*+/del_1076-1079*^) lacking four amino acids in the SET domain of G9a that corresponds to the deletion identified in patient P1 (Supplementary Fig. 15).

*Ehmt2*^*+/del_1076-1079*^ and littermate *Ehmt2*^*+/+*^ control mice used in the studies were generated by in-vitro fertilization. None (out of 13) *Ehmt2*^*+/del_1076-1079*^ females gave any progeny when mated with fertile *Ehmt2*^*+/+*^ males. Two (out of 18) *Ehmt2*^*+/del_1076-1079*^ males reproduced once with fertile *Ehmt2*^*+/+*^ females.

**Fig. 6:**
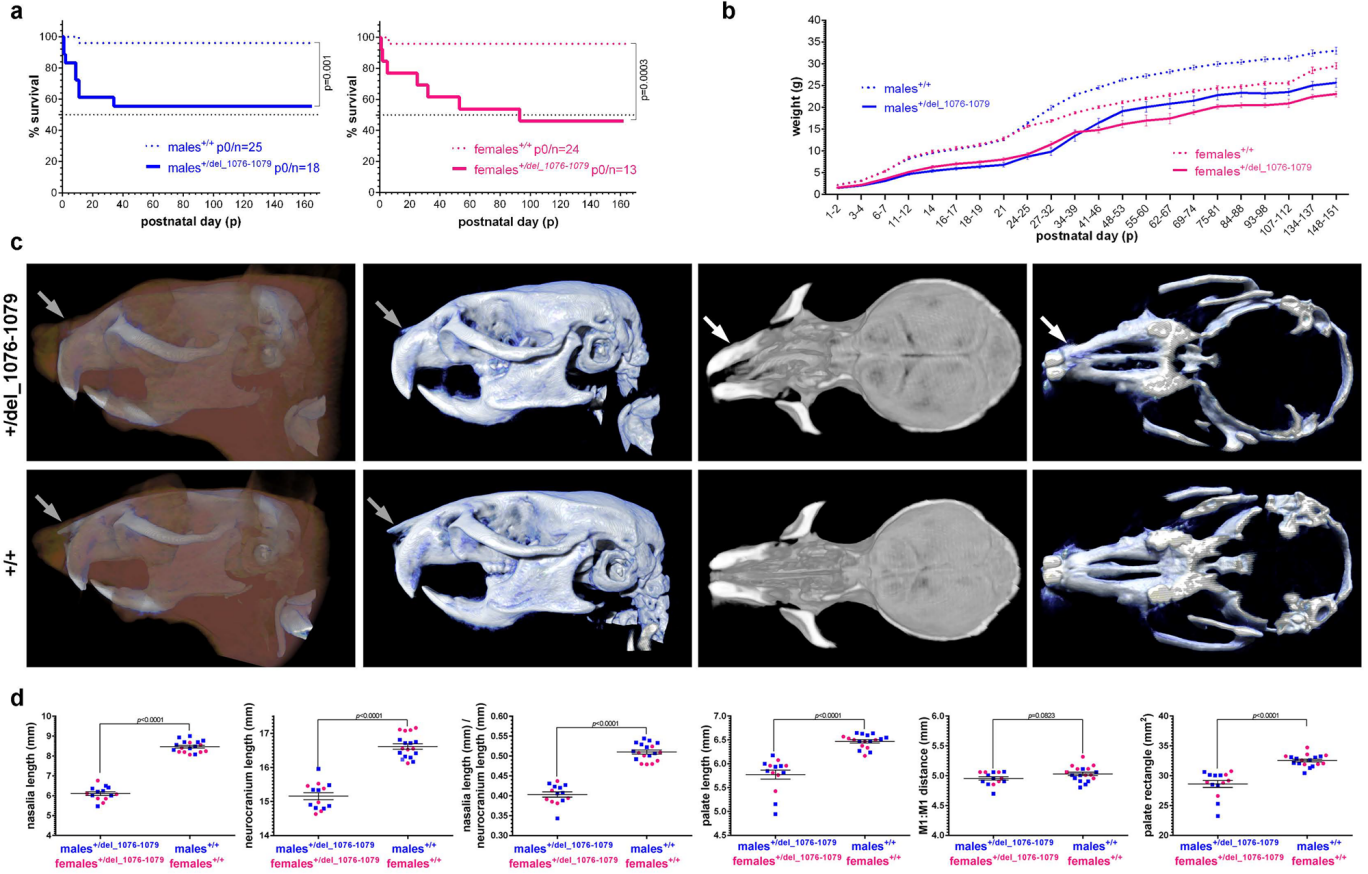
Key phenotypic features identified in *Ehmt2*^*+/del_1076-1079*^ mice. **a** Survival data (5 months) comparing *Ehmt2*^*+/del_1076-1079*^ *and Ehmt2*^*+/+*^ mice are shown for each sex. p0/n corresponds to the starting number of animals included into the study. Black dotted line highlights 50 % survival rate. Statistical significance was calculated using the log-rank (Mantel-Cox) test. **b** Weight curves (5 months) for all animals of both genotypes/sexes included in the survival study. Weights were significantly different (*p* < 0.05 by a two-tailed Student’s t-test) between the *Ehmt2*^*+/del_1076-1079*^ and *Ehmt2*^*+/+*^mice of each sex at all postnatal time points. Refer to Supplementary Fig. 16 for detailed weight comparisons at p1-2 and p21 and weight curves of animals surviving the entire 5 months period and to Supplementary Table 4 for number of animals for each time-point/genotype/sex. **c** MicroCT analyses in *Ehmt2*^*+/del_1076-1079*^ *and Ehmt2*^*+/+*^ mice. Lateral views of the skulls with (in brown) and without soft facial tissues, horizontal views of the nasal cavity and neurocranium, and 3D reconstruction of the palate (bottom view). Nasalia (gray arrows) are shorter in *Ehmt2*^*+/del_1076-1079*^ mice. White arrows highlight the “bent” nose phenotype in *Ehmt2*^*+/del_1076-1079*^ animals. For the proportion and range of the shorter nasalia and the “bent nose” abnormalities see Supplementary Figs. 18-19. **d** Quantitative microCT craniometric data comparisons in *Ehmt2*^*+/del_1076-1079*^ *and Ehmt2*^*+/+*^ mice. *Ehmt2*^*+/del_1076-1079*^ have significantly shorter nasalia and disproportionately shorter neurocranium, which results in a significantly lower ratio of these two values in comparison to *Ehmt2*^*+/+*^ mice. Palate length is shorter in *Ehmt2*^*+/del_1076-1079*^ while the palate width (molar1: molar1 – M1:M1 distance) remains comparable (*p* = 0.083) to *Ehmt2*^*+/+*^ mice. As a result, the palate (calculated as a square size of the latter two values) is significantly smaller in *Ehmt2*^*+/del_1076-1079*^ animals. Statistical significance was calculated using a two-tailed t-test. Values are color/shape-coded based on sex. See Supplementary Fig. 17 for technical and acquisition details of the presented craniometric measurements. B and D show mean values with standard errors. Evaluators were blinded to the genotype of individual mice.

Approximately half of the *Ehmt2*^*+/del_1076-1079*^ animals of both sexes did not survive over postnatal day (p) 30-40 (Fig. 6a). We were able perform urgent autopsies and/or retrieve carcasses for tissue analyses from 7 animals. Gross anatomical and histological analyses did not show any cardiac developmental defects. All surviving mice were terminated no later than p168. In comparison to sex-matched *Ehmt2*^*+/+*^ mice, *Ehmt2*^*+/del_1076-1079*^ presented with significant (and long-term lasting) weight-gain deficits detectable as early as p1-2 (Fig. 6b, Supplementary Fig. 16).

At 3-4 months of age, mice were subjected to facial/skull morphology assessment by microCT, SHIRPA scoring, and a battery of behavioral tests (open field, gait analysis, rotarod and grip strength), and echocardiography. MicroCT and craniometric analyses identified a systematically occurring dysmorphia comprised of a saddle and “bent” nose with shorter nasalia, nasal cavity disorganization, a shorter and smaller palate, and a smaller (shorter) neurocranium in *Ehmt2*^*+/del_1076-1079*^ mice (Fig. 6c, d, Supplementary Figs. 17–19). Importantly, sphenoid bone formation did not seem to be impacted in *Ehmt2*^*+/del_1076-1079*^ in comparison to *Ehmt2*^*+/+*^ animals (Supplementary Fig. 20).

The overall SHIRPA scores were comparable between the *Ehmt2*^+/del_1076-1079^ and *Ehmt2*^+/+^ animals of both sexes (Supplementary Data 5), but the locomotor activity was significantly lower in *Ehmt2*^*+/del_1076-1079*^ animals (Supplementary Fig. 21). This observation was consistent with results of the open-field test, in which *Ehmt2*^*+/del_1076-1079*^ mice traveled significantly shorter distances at lower speeds and rested more than their *Ehmt2*^*+/+*^ littermates. Interestingly, no difference in center zone preference/avoidance suggestive of anxious behavior was observed between the two alternative genotypes/sexes (Supplementary Fig. 22). Gait analysis of *Ehmt2*^*+/del_1076-1079*^ mice reveals a clear phenotype of compromised motor control and stability during the critical transitions of the locomotive cycle, evidenced by prolonged braking times and reduced maximal rates of paw contact area change during the braking phase. These deficits are compounded by postural abnormalities, including increased limbs midline distance and decreased paw overlap - that signal a fundamental breakdown in both intra- and inter-limb coordination (Supplementary Fig. 23, Supplementary Movie 1, Supplementary Movie 2).

Similarly to the open field test, *Ehmt2*^*+/del_1076-1079*^ mice had significantly shorter overall times spent and distances traveled on the rotating rod (rotarod test). While not significantly different, *Ehmt2*^*+/+*^ mice had higher average difference in the times-to-fall between the first and last (6th) trial on the beam (Supplementary Fig. 24). Last, grip strength values were comparable between the two genotypes (Supplementary Fig. 25). Transthoracic echocardiography did not identify any major structural or functional cardiac abnormalities in *Ehmt2*^*+/del_1076-1079*^ mice, as left ventricular morphology and systolic function were largely preserved, and the observed reduction in cardiac output was secondary to a lower heart rate rather than intrinsic ventricular dysfunction (Supplementary Figs. 26–29).

Together, the mouse model verified in vivo the pathogenicity of the identified heterozygous *EHMT2* variant.

## Discussion

Here, we report for the first time clinical and molecular correlates of de novo pathogenic heterozygous loss-of-function variants in the *EHMT2* gene in patients with clinical characteristics overlapping Kleefstra syndrome 1. Specifically, the probands reported here had recognizable facial dysmorphism (hypertelorism, synophrys, flat face, everted lips, protruding jaw) (See Fig. 1) and cardiac anomalies (septal defects, pulmonal valve stenosis, aortal coarctation). Moreover, the probands exhibited additional phenotypic symptoms typical for Kleefstra syndrome 1, including psychomotor developmental delay, intellectual disability, absent speech, structural brain anomalies, hypotonia, dental anomalies, and behavioral and sleep difficulties. These symptoms were observed in all patients except P3, whose clinically milder phenotype was reduced to dysmorphic features and paroxysmal hyperkinetic movement disorder. The clinical similarity of these patients to those with Kleefstra syndrome 1 caused by *EHMT1* haploinsufficiency, was accompanied by their similar episignature profiles in blood, indicating an overall similarity and overlap in the methylation profiles in these two syndromes. Similar clustering of episignatures has been reported for epigenetic disorders with malfunction of the BAF complex due to various mutations in its individual subunits^39^. Despite the overlap with the Kleefstra syndrome 1 episignatures, we identified a distinct set of differentially methylated probes that form a robust, condition-specific episignature for *EHMT2* that can be used to reliably identify additional patients with pathogenic *EHMT2* variants, as demonstrated for patient P7. This probe set provides a highly accurate, sensitive, and specific classifier that clearly differentiates *EHMT2* patients from EHMT1 patients and patients with other disorders with known episignatures. Most probes in this episignature were hypomethylated compared to healthy controls and mapped to genes involved in development. These findings agree with the reported role of G9a in maintaining DNA methylation at germline genes during mouse embryogenesis^34^. Overall, our observations indicate that mutations in *EHMT1* and *EHMT2* affect the same epigenetic module, albeit with specific functional differences. These findings further highlight the value and clinical relevance of episignature analysis in assessing variants of uncertain significance.

Consistent with the aberrant episignature profiles, we observed alterations in histone modifications and gene expression profiles in patient-derived cells. In line with our findings in fibroblasts, coordinated downregulation of H3K9me2 and upregulation of H3K9ac levels have been described in mouse embryonic stem cells knock-out for G9a^24^. Moreover, similar global changes in histone modifications have been observed by mass spectrometry in HEK293 cells with *EHMT1* and *EHMT2* knockdown, including decreased H3K9 methylation and increased levels of methylated H3K79^40^. Patient-derived iPSCs exhibited only minor changes in histone modifications, limited to a subtle yet significant decrease in H3K9 methylation, which could be explained by low levels of this mark in iPSCs compared to fibroblasts (Supplementary Fig. 5B). These differences are consistent with previous reports describing a significant reduction of the repressive marks H3K9me2/3 and H3K27me2/3 during the reprogramming of mouse embryonic fibroblasts to pluripotency^41^ and their re-establishment during differentiation to silence lineage-inappropriate gene expression^42^. Accordingly, changes in histone modifications were accompanied by upregulation of genes involved in alternative lineages, such as nervous, muscle, and skeletal system development, as well as genes involved in cell cycle and Rho signaling pathways. This upregulation is concordant with previously reported gene expression changes resulting from G9a dysfunction^43–47^. Consistent with the presence of an *EHMT2*-specific episignature, we identified gene sets and functional enrichments that were selectively upregulated in *EHMT2* patients‘ cell lines. Whether these unique signatures reflect *EHMT2*-specific molecular functions or a stronger perturbation of the common *EHMT1*/*EHMT2* epigenetic program remains to be determined.

Kleefstra syndrome 1 phenotype was recapitulated in the mouse model (*Ehmt2*^*+/del_1076-1079*^) harboring a heterozygous *Ehmt2* variant equivalent to Del 1076-9 (identified in patient 1). *Ehmt2*^*+/del_1076-*1079^ mice exhibited significantly impaired postnatal survival, growth, and weight-gain retardation, decreased locomotor activity, gait abnormalities, and were mostly infertile. Cranial/facial abnormalities identified in these mice resembled the dysmorphic features observed in the patients and, in specific details (e.g., “bent” nose) overlapped with pathologies earlier detected in the *Ehmt1*^*+/-*^ mouse model^18^.

All the identified *EHMT2* variants disturbed the catalytic site of the SET domain and resulted in a loss of methyltransferase activity. Interestingly, all patients presenting with classic symptoms of Kleefstra syndrome 1 carried *EHMT2* mutations that affected the histone H3 binding site. Conversely, patient P3 with the milder phenotype was heterozygous for the variant N1112S that affects the SAM binding pocket. In addition, the episignature of this patient clustered more closely with Kleefstra syndrome 1 than with the other *EHMT2* patients. These observations suggest that the severity of the phenotype depends on whether the defective enzyme-substrate interactions affect the binding affinity towards histone H3 tail or SAM.

Given the functional interdependency between GLP and G9a, it is remarkable that, twenty years after the initial description of Kleefstra syndrome 1, no individuals with *EHMT2* alterations have been reported. Kleefstra syndrome 1 is commonly caused by microdeletions or point mutations in *EHMT1,* leading to decreased GLP protein levels^22,48^. In agreement with previous reports, we describe that GLP pathogenic mutants C1073Y and R1197W are structurally unstable and/or cause impaired dimerization^13,15^, which is compatible with the mechanism of haploinsufficiency. In contrast, the G9a mutants reported here formed structurally stable dimers that were catalytically inactive. Moreover, *EHMT2* variants did not affect the levels of transcript and protein of both *EHMT1* and *EHMT2* in blood and patient-derived cell lines, nor did they affect the ability to form a stable GLP-G9a complex in the patients’ cells. These findings suggest that the pathogenic mechanism of *EHMT2* variants differs from that of *EHMT1*, which is consistent with previously published data showing complementarity of GLP and G9a enzymes but non-overlapping functions^49^.

Population genomics also points out a different mechanism of pathogenesis for both genes. The Genome Aggregation Database (gnomAD 4.1.0) shows marked differences in the probability of loss-of-function intolerance scores for *EHMT1* (LOEUF = 0.16) and *EHMT2* (LOEUF = 0.49) suggesting a high level of intolerance to loss of *EHMT1* function, in contrast to *EHMT2*. Similarly, the Database of Genomic Variants (DGV) shows marked differences in occurrence of genic deletions in the healthy population that are sporadic for *EHMT1*, but frequent for *EHMT2* (Supplementary Fig. 30a). Consistently, the Database of Chromosomal Imbalance and Phenotype in Humans Using Ensembl Resources (DECIPHER), a repository for variants identified in patients with rare diseases, shows a high frequency of *EHMT1* deletions, but not that of *EHMT2* (Supplementary Fig. 30b). Additionally, gnomAD v4.1.0 documented numerous patients of variants (6 variants with 27 allele counts) positioned in methyltransferase active site that were specifically identified in *EHMT1* and not in *EHMT2*. Of particular note is the *EHMT1* variant N1200S, an equivalent to N1112S in the *EHMT2* patient described here, which was reported in 14 individuals in gnomAD v4.1.0. In summary, the population genomic data support a notion that *EHMT2* haploinsufficiency is not sufficient to cause disease, in contrast to *EHMT1*.

**Fig. 7:**
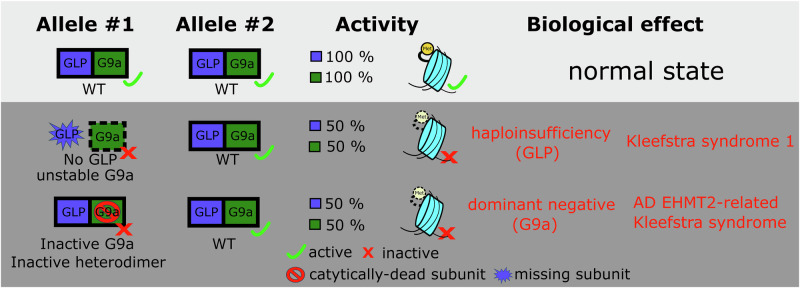
Scheme showing various scenarios for partially dysfunctional GLP/G9a heterodimer, that lead to physiological (upper part) or aberrant (lower part) H3K9 methylation.

Additional evidence supporting a differential mechanism of pathogenesis for *EHMT1* and *EHMT2* is provided by the mouse model. *Ehmt1*^⁺/⁻^ mice have been reported to exhibit significant changes in histone methylation and transcriptome profiles, leading to severe cognitive impairments^17^. In contrast, *Ehmt2*^⁺/⁻^ mice display only mild alterations in epigenetic and transcriptomic profiles, resulting in minor phenotypic effects, according to two independent studies^19,24^. However, our mouse model (*Ehmt2*^*+/del_1076-1079*^) displays severe phenotype abnormalities. This suggests that the reported SET-domain variants are more pathogenic than the heterozygous loss of *EHMT2*. Overall, our experimental results, population genetics data, and phenotypes of the mouse model suggest that the described *EHMT2* pathogenic variants probably act via dominant-negative effects (Fig. 7).

Mechanistically, differences in the pathogenic mechanisms of *EHMT1* and *EHMT2* can be explained by the different biochemical properties and cellular functions attributed to both enzymes. While mouse embryonic stem cells deficient in GLP show significantly reduced levels of G9a protein, G9a-deficient cells exhibit normal GLP levels^6^. These findings indicate that G9a protein stability is highly dependent on the presence of GLP, while GLP stability is independent of G9a. Therefore, G9a’s biological function seems to be exclusively exercised through the G9a/GLP heterodimer, whereas GLP might be capable of forming both homo- and heterodimeric complexes.

In addition, each subunit of the GLP/G9a complex plays a distinctive role in governing the physiological levels of H3K9 methylation. Specifically, GLP contributes to the propagation of H3K9 methylation via recognition of H3K9me1/2 modified nucleosomes through its ankyrin-repeats domain (denoted as reader function)^49^, whereas G9a executes the methylation reaction per se by its enzymatically active SET domain^25^. Consistently, a recent report evaluating 209 individuals with rare variants in *EHMT1* found that missense variants in the GLP ankyrin repeat domain, resulting in a structurally stable GLP protein yet impairing its reader function, were associated with Kleefstra syndrome 1^15^. In addition, variants that disrupted the GLP methyltransferase activity without affecting its structural stability could not be correlated with Kleefstra episignatures and phenotype^15^. These data indicate that both normal structural stability and reader function of GLP together with G9a writer activity are necessary conditions for the proper function of the GLP/G9a complexes. It is therefore plausible that pathogenic variants in *EHMT1*, but not in *EHMT2*, lead to haploinsufficiency caused by the reduced number of functional dimeric units of GLP/G9a. This reduction might be compensated by the formation of GLP homodimers in the case of limited G9a availability (e.g., in *Ehmt2*^*+/-*^ mice), which seems unlikely to apply in the reverse scenario. In contrast, the pathogenic variants in *EHMT2* identified in this study give rise to stable GLP/G9a complexes with severely impaired nucleosomal methylation activity. These inactive mutant complexes might exert dominant-negative effects by binding to chromatin and blocking the access of the other functional complexes, resulting in disrupted epigenetic regulation.

In summary, we demonstrate that *EHMT2* missense variants located in the SET domain, which impair its catalytic function, lead to clinical symptoms that mimic Kleefstra syndrome 1. This is supported by Kleefstra syndrome 1-like epigenetic changes, aberrant biochemical behavior of the mutant proteins and recapitulation of Kleefstra syndrome 1 phenotype in the mouse model. We also provide additional evidence that *EHMT2* and *EHMT1* variants act through different pathogenetic mechanisms in the context of the GLP/G9a complex. Therefore, the characterized patients with pathogenic *EHMT2* variants establish an autosomal dominant *EHMT2*-related Kleefstra syndrome. We believe that our findings will further facilitate the identification of additional individuals affected by pathogenic *EHMT2* variants and promote further research of the physiological and pathogenetic aspects of GLP/G9a biology.

## Methods

### Ethics Declaration

Informed consent for genetic analyses was obtained for all individuals, and genetic studies were performed as approved by the review boards of the corresponding institutes. The patient’s parents provided written informed consent for participation in the study, clinical data and specimen collection, genetic analysis, publication of relevant findings with potentially identifiable information and use and publication of their photographs. All data were de-identified. The study adheres to the principles set out in the Declaration of Helsinki. The study was approved by the corresponding author’s institutional ethical review board (Ethics Committee of the General University Hospital in Prague, no. 34/22). The generation of human iPSCs was approved by the ethical committee of the Instituto de Salud Carlos III CEI PI 03_2022. Informed consents for sample donation and iPSCs generation were signed by the legal guardians. The animal study was approved by the Animal Care and Use Committee of the Institute of Molecular Genetics (AVCR 7341/2021 SOV II, AVCR 6144/2022 SOV II) and conducted in accordance with national and EU guidelines for the care and use of laboratory animals.

### Identification of *EHMT2* variants

Variants in *EHMT2* were discovered by exome or genome analyses. Detailed data analysis workflow for individual patients is summarized in Supplementary Information.

### DNA methylation data analysis

#### Data generation and processing

Methylation analyses were conducted using the clinically validated EpiSign assay, following previously established protocols^28–30,50^. All computational analyses were performed using R statistical software^51^ (version 4.4.1) along with relevant libraries. Raw intensity data files containing methylated and unmethylated signal values underwent quality control using the SeSAMe package^52^. Standard preprocessing of Illumina microarray data involves dye bias correction, background subtraction, and masking of low-quality probes^53^. Detection *p*-value-based probe masking is further applied using Infinium I out-of-band signal calibration, with a significance threshold set at *p* = 0.05.

#### Episignature discovery

A signature discovery analysis was conducted using six case samples (P1-P6) carrying variants in the SET domain of *EHMT2*. Before analysis, probes were excluded if they met any of the following criteria: located on the X or Y chromosomes, targeting CpG sites overlapping known single nucleotide polymorphisms (SNPs), identified as cross-reactive, exhibiting high variability in previous Illumina DNA methylation array versions following manufacturing changes, or listed among EPICv1 probes that were omitted in the updated EPICv2 array. Matched control samples for the discovery analysis were selected using the ‘matchIt’ package^54^, with matching criteria based on age, sex, and the array type used for methylation profiling. Samples from the EpiSign™ Knowledge Database (EKD) previously identified as contributing to batch effects, as well as those with more than 5% failed probes, were excluded during the matching process. A total of 54 matched controls were included in the analysis, yielding a match ratio of 1:9. Principal component analysis (PCA) was subsequently performed to assess data structure and identify potential outliers.

Differential analysis was conducted on the discovery cohort using the ‘limma’ package^55^. Methylation beta values were first transformed into M-values via a logit transformation. The data were then modeled using multivariate linear regression, where methylation levels served as predictors, case/control status as the response variable, and estimated blood cell compositions as covariates. To enhance statistical reliability, empirical Bayes moderation was applied, and adjusted *p*-values were calculated using the Benjamini-Hochberg method to control the false discovery rate. To identify probes that define the episignature, a multi-step ranking process was employed. Initially, probes were ranked based on a composite score derived from the absolute mean methylation difference and the negative logarithm of their adjusted *p*-value. The top subset of these probes was then selected using ROC curve analysis implemented via the ‘caret’ package^56^. Highly correlated probes were subsequently removed to reduce redundancy. The resulting probe lists were evaluated using unsupervised clustering, including Euclidean distance-based clustering with Ward’s method and multidimensional scaling. Visualization was performed using the ‘ggplot2’^57^ and ‘gplots’^58^ packages in R. The probe set that yielded the most distinct clustering was chosen to define the final signature.

The selected probes were utilized to construct a support vector machine (SVM) classification model using the e1071 package^59^ as previously described^20,39,50^. For each sample, the classifier generates a Methylation Variant Pathogenicity (MVP) score ranging from 0 to 1. An MVP score approaching 1 indicates a methylation pattern closely resembling the *EHMT2* episignature, whereas scores near 0 reflect a pattern similar to that of control samples. The SVM classifier was trained using the discovery case samples, matched controls, 75% of the remaining control samples, and 75% of the neurodevelopmental disorder samples from the EKD. The remaining 25% of control and neurodevelopmental disorder samples were reserved for model testing. SVM hyperparameters, including class weights, regularization parameters, and kernel functions, were optimized via grid search.

To evaluate the reproducibility and robustness of the identified methylation profile, six rounds of leave-one-out cross-validation were performed on the discovery case samples. In each round, one of the six *EHMT2* samples was excluded from probe selection, and the remaining five were used to train the model. The excluded sample was then used for testing and visualized using heatmaps and multidimensional scaling (MDS) plots. To provide an additional independent assessment, the seventh sample in the cohort was subsequently used as a test validation case.

### Cell cultures

The human samples have been obtained through the biobank of rare diseases BioNER https://bioner.isciii.es/home/.

#### Fibroblast culture

Human dermal fibroblast cultures were established from skin tissue samples. Briefly, fibroblasts were mechanically isolated by dissecting the dermal layer of the skin, and the resulting fragments were incubated at 37 °C in Dulbecco’s modified Eagle’s medium (DMEM) containing 2% of fetal calf serum. Cells were expanded in 75 cm^2^ culture flasks at 37 °C with 5% CO_2_ and 95% humidity in DMEM containing 10% fetal bovine serum.

#### Reprogramming to pluripotency

Cell lines were generated from cultured fibroblasts or peripheral blood mononuclear cells using the CytoTune iPS 2.0 Sendai Reprogramming Kit (Invitrogen). Growing colonies were manually selected according to morphology and passed for at least seven passages before assessing the presence of viral mRNAs.

#### Maintenance of iPSCs

Established cell lines were grown in Essential E8 Media (Gibco) in Vitronectin (Gibco) coated plates. Cells were split twice a week using ReLeSR (Stem Cell Technologies) at a ratio of 1:4-1:10 in 5 µM Rock inhibitor Y-27632 (Tocris) for 1–3 h before changing to fresh media.

#### RNA preparation and real-time PCR

RNA was extracted using RNeasy kit (Qiagen) and cDNA prepared using the Maxima First Strand cDNA Synthesis Kit. Oligonucleotides for detecting the expression of transgenes were the ones recommended in the reprogramming kit. Clones free from viral expression were selected for further characterization. Oligonucleotides used to detect the expression of pluripotency genes and reprogramming factors were purchased from Merck and have been previously described^60^.

#### Immunofluorescence staining

Cells were fixed using 4% PFA for 20 min at room temperature and washed with phosphate-buffered saline (PBS) three times, followed by permeabilization and blocking with 0.25% Triton X-100 and 5% normal horse serum at room temperature for one hour. Subsequently, cells were incubated with primary antibodies (anti-Sox2 AB5603 Sigma and PA1-094 Fisher Scientific (1:400), anti-NANOG AF1997-SP R&D (1:50), anti-Oct3/4 (C-10) sc-5279 Santa Cruz Biotechnology, anti-TRA-1-60 sc-21705 Santa Cruz Biotechnology, anti-Alpha-1-fetoprotein A000829-2 Agilent Dako (1:500), Alpha-Smooth Muscle A5228 Sigma (1:500), TUBB3 Biolegend 801202 (1:500)) overnight at 4°C. The next day, cells were washed with PBS and incubated with secondary antibodies for 1 h at room temperature in the dark and stained with DAPI. Images were captured using a laser confocal microscope (Stellaris 8, Leica Microsystems).

#### Embryoid body (EB) differentiation

For in vitro differentiation, cells were trypsinized using ReLeSR, and 50,000 cells per well were seeded into low-attachment 96-well v-bottom plates in Essential 8 media and 5 µM Rock inhibitor Y-27632 (Tocris). After two days, EBs were moved from the plate to a 10 cm low attachment plate with fresh Essential 8 media. The next day, they were transferred to gelatin-coated dishes and cultured in differentiation media consisting of DMEM media supplemented with 20% fetal bovine serum for up to twenty days.

#### Karyotype and genotype

The generated iPSC lines were free from genetic abnormalities according to the hPSC Genetic Analysis Kit from Stem Cell Technology and karyotype from 20 metaphases. To confirm the presence of the mutations, genomic DNA was extracted with the DNeasy Blood and Tissue Kit from Qiagen, and mutations were verified by amplification and Sanger sequencing. Oligonucleotides used for amplifying and sequencing the *EHMT1* gene were primer forward: 5’-CTTCTTCTCTGTGGGGCGAG-3’ primer reverse: 5’-CACCATAAGCATCAGCATCAGC-3’ and the *EHMT2* gene primer forward 5’TCCATATCGCCCATTCCTGC-3’ and primer reverse 5’-ATGAAGCGGCTGATGTTGCC-3’. These custom oligonucleotides were purchased from Merck.

### RNA-seq analysis

Total RNA was extracted from fibroblasts and iPSCs using the RNeasy mini kit from Qiagen. RNA-seq was performed at BGI Tech Solutions with two or three biological replicates per condition. Briefly, ribosomal RNAs were removed using an RNase H-based method, and first-strand cDNA was generated using random hexamer-primed reverse transcription, followed by a second-strand cDNA synthesis with dUTP instead of dTTP, end repair, A addition, and adapter ligation. The U-labeled second-strand template was digested with Uracil-DNA-Glycosylase (UDG) and amplified by PCR. The resulting library was validated by quality control. The PCR products were then heat denatured and circularized by the splint oligo sequence to generate a single strand circle DNA, followed by rolling circle replication to create DNA nanoballs (DNB) for sequencing on the MGI DNBSEQ platform. Raw sequencing data with adapter sequences or low-quality sequences were trimmed or filtered and examined by FastQC for basic quality controls. The sequencing analysis was carried out using Galaxy (https://usegalaxy.eu/). Paired reads were aligned to the human hg19 genome build using STAR^61^. Gene counts were calculated using HTseq-count^62^, and differential expression between patients and healthy fibroblasts was interrogated using DEseq^63^. Differentially expressed genes were considered at an adjusted *p*-value < 0.05. PCA plots were generated using the DESeq2 package in R software (version 4.4.1). Enrichment analysis in differentially expressed genes was carried out using DAVID^64^. Scatterplots showing correlations were generated with ggplot2 (https://ggplot2.tidyverse.org) in Galaxy. Pearson’s correlation and bubble plots were calculated using SRPlot^65^. Heatmaps were generated using Morpheus (https://software.broadinstitute.org/morpheus/).

### Proteomic mass spectrometry for histone extracts

Proteomic analyses were performed by Active Motif (Waterloo, Belgium). Histones were acid extracted from a cell pellet containing 2.5×10^6^ cells, derivatized via propionylation, digested with trypsin, and newly formed N-termini were propionylated as previously described^66^. Histones were extracted by incubating samples at room temperature for 1 h in 0.2 M sulfuric acid with intermittent vortexing. Histones were then precipitated by the addition of trichloroacetic acid (TCA) on ice and recovered by centrifugation at 10,000 × g for 5 min at 4 °C. The pellet was then washed once with 1 mL cold acetone/0.1% HCl and twice with 100% acetone, and then air dried in a clean hood. The histones were propionylated by adding 1:3 (v/v) propionic anhydride/2-propanol and incrementally adding ammonium hydroxide to keep the pH around 8, and subsequently dried in a SpeedVac concentrator. The pellet was then resuspended in 100 mM ammonium bicarbonate and adjusted to pH 7-8 with ammonium hydroxide. The histones were then digested with trypsin, resuspended in 100 mM ammonium bicarbonate overnight at 37 °C, and dried in a SpeedVac concentrator. The pellet was resuspended in 100 mM ammonium bicarbonate and propionylated a second time by adding 1:3 v/v propionic anhydride/2-propanol and incrementally adding ammonium hydroxide to keep the pH around 8, and subsequently dried in a SpeedVac concentrator. Histone peptides were resuspended in 50 µL of 0.1% TFA, and 3 µl were injected in 3 technical replicates in a Thermo Scientific TSQ Quantum Ultra mass spectrometer (Thermo Scientific) coupled with an UltiMate 3000 Dionex nano-liquid chromatography system. The data was quantified using Skyline^67^ and represents the percent of each modification within the total pool of that amino acid residue. A total of 92 modification states were quantified, including unmodified ones.

### Western blot analyses of fibroblasts and iPSCs

Cell extracts were prepared in a lysis buffer (0.9× CelLytic MT reagent supplemented with 200 mM NaCl, 1.5 mM magnesium chloride, 20 mM EDTA and protease inhibitor cocktail) with gentle shaking for 30 min at 4 °C. Crude cell extracts were centrifuged for 16,000×g for 10 min at 4 °C. Supernatants were collected, and protein concentration was determined using the Bradford assay with bovine serum albumin as standard. Subsequently, extracts were analyzed using SDS-PAGE (4–12% gradient gels) followed by western blotting (with 20 µg and 10 µg of total protein loaded in the case of fibroblasts and iPSCs, respectively) with immunodetection against G9a (rabbit anti-G9a, 1:1000; Sigma, G 6919), GLP (rabbit anti-GLP, 1:1000, Sigma, 09-078) and GAPDH (mouse Anti-GAPDH, 1:10000; Sigma, G8795) as a reference.

### Immunoprecipitation (IP) of G9a

Dynabeads Protein A (ThermoFisher Scientific) were coated with rabbit anti-G9a or rabbit anti-PFAS IgG as a negative control (1 ug per 50 ul of Dynabeads suspension; ab185050 and ARP46181 from Abcam and Aviva Systems Biology, respectively) in PBS containing 0.1% Tween 20 for 10 min at room temperature. Whole cell extracts from iPSCs (100 µg of total protein) were added to antibody-coated Dynabeads and incubated with rotation for 45 min at 4 °C. After three washing cycles (0.9× CelLytic MT reagent supplemented with 200 mM NaCl, 1.5 mM magnesium chloride, and 20 mM EDTA), immunoprecipitated proteins were eluted by 50 mM Glycine (pH 2.8) followed by the addition of 100 mM Tris-HCl (pH 7.5). The eluate was analyzed by western blotting against G9a and GLP (see above) and by mass spectrometry.

#### Mass spectrometric analysis of eluates from IP

Tris(2-carboxyethyl)phosphine (TCEP) and 2-chloroacetamide were added to final concentrations of 10 mM and 40 mM, respectively, and samples were incubated at 70 °C for 10 min. Samples were then added to Sera-Mag Carboxylate SpeedBeads (Cytiva), diluted with ethanol to a final concentration of 50% (v/v) and incubated for 10 min on a rocker at 1000 rpm to allow protein binding. Beads were washed three times with 80% (v/v) ethanol. Proteins bound to beads were digested with Lys-C (Promega) for 2 h at 37 °C, followed by the addition of trypsin (Promega) and overnight digestion at 37 °C. Peptides were desalted using homemade StageTips containing three punches of Empore SPE C18 disks (Merck).

LC–MS/MS analysis was performed using a Vanquish Neo UHPLC system (Thermo Fisher Scientific) coupled to a timsTOF SCP mass spectrometer (Bruker Daltonics). Peptides were separated on a reversed-phase PepSep C18 analytical column (15 cm × 75 µm; Bruker Daltonics) using solvent A (2% ACN, 0.1% formic acid) and solvent B (80% ACN, 0.1% formic acid). A 60-min linear gradient from 1 to 35% solvent B was applied. Data were acquired in data-dependent acquisition (DDA) mode.

Raw data were analyzed using PEAKS 12.5 software with a parent mass error tolerance of 20 ppm and a fragment mass error tolerance of 0.05 Da. Carbamidomethylation of cysteine was specified as a fixed modification, while N-terminal acetylation and methionine oxidation were set as variable modifications, with a maximum of three variable modifications per peptide. Database searches were performed against the human UniProt database. This analysis was performed in three replicates.

### Functional studies

#### In-silico structural mapping

Mutations were mapped onto the crystal structure of G9a SET-domain bound to the N-terminal peptide of histone H3 and S-adenosylmethionine (PDB ID 5JIN)^68^. Structural models were visualized and assessed by Pymol Viewer 2.1 (Schrodinger, LLC.). Representative images were prepared using UCSF ChimeraX 1.6.1^69^.

#### Preparation of recombinant proteins

The methyltransferase domains of G9a (aa residues 913–1193) and GLP (aa 1001–1266) with an N-terminal hexa-histidine tag followed by a recognition site for TEV protease were expressed in *E.coli* (BL21-CodonPlus (DE3)-RIPL strain) using pET28a plasmid (synthesized in Gene Universal). For co-expression of GLP (aa residues 710–1285) and G9a (aa residues 601–1193), both constructs comprising N-terminal hexa-histidine tags with a recognition site for TEV protease, the ankyrin repeats, and the methyltransferase domain in pETDuet-1 were used (synthesized in Gene Universal). Mutations were generated using Q5 Site-Directed Mutagenesis Kit (New England Biolabs) with corresponding primers (Generi Biotech) listed in Supplementary Table 5. Bacterial culture was cultivated in Terrific Broth medium supplemented by 0.1 mM zinc chloride. When cells reached an OD600 of 1.0, protein expression was induced by the addition of 0.5 mM IPTG at 15 °C for 24 h.

After cell lysis by sonication in lysis buffer (G9a: 20 mM potassium phosphate (pH 7.5) containing 500 mM NaCl, 20 mM imidazole, 10 mM beta-mercaptoethanol, 5% glycerol and SigmaFast inhibitor cocktail; GLP: 50 mM Tris-HCl (pH 7.5) containing 500 mM NaCl, 20 mM imidazole, 0.5 mM TCEP, 10% glycerol and SigmaFast inhibitor cocktail), proteins were purified by affinity chromatography using an imidazole gradient from 20 to 500 mM over 20 column volumes with Profinity IMAC column (Biorad) followed by cleavage of the histidine tag by TEV-protease overnight at 4 °C (protein:protease w/w ratio of 50:1). In the next step, proteins were separated using size-exclusion chromatography (Enrich SEC 650 column, BioRad) in SEC buffer (G9a: 50 mM potassium phosphate (pH 7.5) containing 100 mM NaCl, 2 mM dithiotreitol, 20 mM imidazole and 5% glycerol; GLP: 50 mM Tris-HCl (pH 7.5) containing 100 mM NaCl, 0.5 mM TCEP and 10% glycerol) and a second round of affinity chromatography to remove TEV protease containing uncleavable histidine tag. Finally, proteins were dialyzed against storage buffer (G9a: 50 mM potassium phosphate (pH 7.0) containing 100 mM NaCl, 1 mM TCEP and 5% glycerol; GLP: 25 mM Tris-HCl (pH 8.0) containing 50 mM NaCl, 0.5 mM TCEP and 10% glycerol), and stored in −80 °C. The GLP–G9a heterodimer was purified using the same protocol as for G9a, as described above. Aminoacid sequence of the purified proteins was verified using on-line pepsin digestion followed by LC-MS/MS analysis (details described in ref.^70^).

#### Solubility test of GLP mutants expressed in E.coli

Bacterial pellets were resuspended in the lysate buffer (as described above) and cells were disintegrated by sonication or by an addition of BugBuster reagent, lysozyme (0.5 mg/ml) and benzonase (25 mU/µl) followed by gentle shaking at room temperature for 20 min. Soluble and insoluble fractions were separated by centrifugation at 4 °C at 16,000 G for 20 min. Amount of soluble GLP proteins in supernatant was determined using western blotting (with 10 µg of total protein loaded) followed by immunodetection using Anti-6× His tag primary antibody (ab9108 from Abcam) and Rabbit IgG (H + L) Secondary Antibody (31460 from Thermo Fisher Scientific). Insoluble fraction was washed four times for by BugBuster solution followed by centrifugation 4 °C at 5000×g for 15 min and analyzed by SDS-PAGE with Coomassie-Blue staining.

#### Bioluminescence-based methyltransferase assay

Enzymatic activity of G9a, GLP, and GLP-G9a variants was assessed using MTase-Glo Methyltransferase Assay (Promega) according to the manufacturer's instructions. The activity assay was performed with a peptide derived from the N-terminal part of histone H3 (aa residues 1–20; manufactured by Merck Life Science) or mononucleosome (prepared according to^71^). Concentration of the histone H3 peptide and S-adenosylmethionine was kept at 10 µM. Methyltransferase reactions were performed at room temperature for 30 min with 5 nM G9a or GLP enzymes. In the assay with mononucleosome, the concentration was kept at 0.5 µM and 5 µM for mononucleosome and SAM, respectively. Reaction was executed at 37 °C for 1 h with 0.5 µM enzyme. For the GLP–G9a heterodimer, nucleosome methylation assays were performed at room temperature for 1 h with 0.25 µM enzyme. Obtained signals were recorded using a Spark plate reader (TECAN). Quantification was performed using an external calibration standard of S-adenosyl homocysteine solution. Kinetic data were fitted according to the Michaelis-Menten equation using OriginPro 2015 (OriginLab).

#### MALDI-TOF MS-based methyltransferase assay

Reactions were performed in 50 mM HEPES (pH 7.9) containing 0.5 mM DTT, 0.25 mM PMSF and 2 mM magnesium chloride with 25 µM histone H3 peptide, 50 µM S-adenosylmethionine and G9a or GLP enzyme (25 or 100 nM) for 30 min at 30 °C. The reaction was quenched by the addition of 0.1% TFA. The resulting mixture was 10-fold diluted in 0.1% TFA, mixed with a saturated solution of α-cyano-4-hydroxycinnamic acid in a ratio of 1:1 (v/v), and deposited onto a MALDI target plate (MTP 384 target plate ground steel, Bruker). Samples were measured using Autoflex Speed (Bruker Daltonics) in a reflection positive mode with parameters as follows: mass range 700–3500 m/z, frequency 1000 Hz and ion extraction delay 130 ns. The instrument was externally calibrated using Bruker Peptide calibration standard mix II. Spectra were processed in FlexAnalysis 3.4 (Bruker Daltonics).

#### Differential scanning fluorimetry

Proteins (3 µM) dissolved in an assay buffer (G9a: 50 mM potassium phosphate (pH 7.0) containing 100 mM NaCl, 1 mM TCEP; GLP: 25 mM Tris-HCl (pH 8.0) containing 50 mM NaCl and 0.5 mM TCEP) were analyzed in a free state or bound to histone H3 peptide (50 or 500 µM) or S-adenosylmethionine (50 or 500 µM). After preincubation of the G9a proteins with ligands at room temperature for 10 min, 5× Sypro-Orange dye (Invitrogen) was added to the samples, which were subsequently subjected to melting analysis using CFX96 Real-Time System (BioRad). The proteins were heated from 20 °C to 90 °C in increments of 0.5 °C and with 1-minute hold intervals. The signal was monitored using fluorescence detection (excitation wavelength of 470 nm and emission wavelength of 570 nm). The melting temperatures (Tm) of the proteins were determined as minima from first derivative curves.

#### Native mass spectrometry

In order to examine the protein dimerization and substrate binding, we performed native MS analysis on a Waters Synapt G2Si instrument. Analyzed G9a, GLP, and GLP-G9a variants were first transferred into 150 mM ammonium acetate, pH 7.5, containing 0.5 mM DTT by one or two cycles of spin gel filtration (Micro Bio-Spin P-6 Gel columns, 6-kDa cut off; Bio-Rad). Next, the protein samples were diluted to 5 μM concentration, optionally mixed with SAM or histone H3 peptide in an equimolar ratio, and incubated for 10 min at 4 °C. The samples were electrosprayed at 0.9–1.2 kV from an in-house prepared borosilicate glass spraying tips^72^. (Kwik-Fil 1B120F-4, World Precision Instruments) pulled with P-97 platinum-wire Flaming/Brown Micropipette puller (Sutter Instrument) and coated with 40 nm thick layer of gold using an ACE600 sputter coater (Leica). Data acquisition was performed in positive ionization sensitivity mode. For assessing the dimerization and substrate binding of the G9a and GLP variants, the trap collision energy was optimized to maximize sensitivity while keeping ion activation minimal at 10 V and 40 V, respectively. The argon flow as collision gas was maintained at 6 ml/min, sampling cone voltage set to 80 V, source offset to 0 V, and source temperature to 80 °C. Quadrupole was operated in broad transmission mode up to 4000 m/z. Acquired data were externally mass recalibrated on cesium iodide clusters, summed over 50 scans, and smoothed using two passes of Savitzky-Golay filtering (window 50 scans)^73^ in MassLynx 4.1 (Waters). Subsequently, mass deconvolution of the spectra was performed in UniDec v6.0.4^74^. Additionally, spectra were background subtracted in UniDec v6.0.4 and used to quantify the monomer-dimer distribution of the analyzed proteins in their apo-form (Supplementary Fig. 12).

### Mouse model

#### Generation of the knock-in mouse model

To model the effects of the variant (c.3225_3236del, p.Glu1076_Val1079del) identified in patient 1, the *Ehmt2* c.3385_G3396del (p.E1129_V1132del, (C57BL/6NCrl-Ehmt2^em1Ccpcz^/Ph) mouse model (further referenced in the manuscript as *Ehmt2*^*+/del_1076-1079*^) was generated on the C57BL/6NCrl background (Charles River Laboratories) by targeting exon 25 (ENSMUSE00001282962) (Supplementary Fig. 15A). *Ehmt2* codons E1129, A1130, D1131 and V1132, that are complementary to human *EHMT2* codons 1076–1079, were deleted using CRISPR/Cas9 technology in combination with single-stranded oligonucleotides (ssODN) template: 5’- GGTGCTGCATCCCACCTTGTTATCTAAATCGAAGAGGTAAGAATCATCCTCTCTCGCATCAGAGATCAGCTCTCCTACGTACCTGTTGGACGGAAACTTCAGTTCTGCATGAACAGTGAGC -3’ as described by ref.^75^. The guide RNAs (gRNAs) of highest score and specificity were designed using http://crispor.tefor.net/.The following guide was selected: gRNA- AGAGCTGATCTCTGATGCCG. The gRNAs (100 ng/µl, Integrated DNA Technologies) were assembled into a ribonucleoprotein (RNP) complex with Cas9 protein (500 ng/µl; 1081058, 1072532, Integrated DNA Technologies), electroporated into 1-cell zygotes, and transferred into ICR (CD1) pseudopregnant foster females. Putative founders were analyzed by PCR and sequencing. A founder carrying a E1129-V1132del mutation also harbors *SnaBI* (Eco105I) restriction enzyme site, enabling direct detection of the mutation by the restriction enzyme digest. Genotyping was performed by PCR with forward (F) 5′- TTCCCTCAACTTCTCAGACACC -3′ and reverse (R)5’- ACCCGGACAGGGATGATGTT -3’ primers. The F and R product is 775 bp, *SnaBI* digestion of the E1129-D1132del homozygote mice PCR product results in 439 bp and 324 bp fragments (Supplementary Fig. 15b, c). The selected founder was confirmed by sequencing and bred with C57BL/6NCrl wild-type to confirm germ-line transmission of the target deletion. The only male founder carrying E1129-V1132del gave rise to infertile females and sub-viable males, often dying in 10 days after birth. Due to this limitation, the founder was archived in the form of frozen sperm. To propagate this line, in vitro fertilization was performed as described previously^76^, and viable embryos were implanted into ICR (CD1) surrogate females (Charles River Laboratories). Animals born after IVF were genotyped and sequenced (Sanger sequencing) within 5 days after birth to detect and monitor positive mice. Bulk sequencing data were further analyzed using DECODR.org online software^77^. The sperm from the founder male was sequenced using NGS sequencing to detect all possible *Ehmt2* gene variants in the germ line.

Mice were housed in individually ventilated cages (IVCs) under controlled environmental conditions with a 12 h light/12 h dark cycle, ambient temperature maintained at 20–24 °C, and relative humidity at 45–70%. Animals had ad libitum access to a standard rodent diet and water. Animal husbandry and welfare monitoring were performed daily by qualified personnel under veterinary supervision.

Evaluators were blinded to the genotype of individual mice in all the following tests.

#### SHIRPA and locomotor activity scoring

Standardized SHIRPA scoring scale^78^ was used to generally assess the phenotype of the *Ehmt2*^*+/del_1076-1079*^ and *Ehmt2*^*+/+*^ mice. Locomotor Activity Test was used for quick evaluation of spontaneous exploratory behavior and general activity level of mice. The test was performed in a room with 100–120 lux light intensity using a Maneko rat cage (55 × 33 cm) with a floor divided into 15 equal squares. Each mouse was placed in the arena and allowed to move freely. Locomotor activity was quantified by counting the number of squares the animal entered during the first 30 s of exploration.

#### In-vivo microCT mouse scanning

All mice were anesthetized via intramuscular administration of 20% Zoletil-Xylazine (Virbac). In-vivo scans of mice were performed using the SkyScan 1278 (Bruker) as continuous seven connected scans. Scanning parameters included a voxel size of 50 μm, a 0.5 mm aluminum filter, 180° rotation, and no averaging. Source voltage was set to 54 kV and source current to 909 µA. Data reconstruction was carried out using NRecon software, version 2.2.0.6 (Bruker), with the following parameters: ring artifact correction at 3, beam hardening at 28%, threshold for defect pixel mask at 5%, no smoothing, and intensity range set from 0.0025 to 0.10 AU^79^. CTvox software, version 3.3.0.0 (Bruker) was used for data visualization. Craniometric measurements (Supplementary Fig. 17) were performed in DataViewer, version 1.5.2 (Bruker).

#### Open Field test

The activity of the animals in a novel environment and the level of anxiety displayed were evaluated in the open field test^80^. The area of the open field was a square of 42 × 42 cm uniformly illuminated with a light intensity of 200 lux in the center of the field. The testing arena was virtually divided into periphery and center zones, where the center zone constituted 38% of the whole arena. Each mouse was placed in the corner of the arena for a 20 min period of free maze exploration. The time spent in each zone, the distance traveled, and other indices were automatically computed based on video recordings (Viewer software 3.0.1.452, Biobserve GmbH).

#### Gait analysis

Gait analysis was conducted using the DigiGait system (Mouse Specifics Inc., Framingham, MA, USA) to assess the specific differences in gait between *Ehmt2*^*+/+*^ (males *n* = 15 and females *n* = 15) and *Ehmt2*^*+/del_1076-1079*^ (males *n* = 8 and females *n* = 6) mice at 3-4 months of age.

Briefly, the DigiGait system consisted of a transparent treadmill, horizontally fixed at 0 ° (5 cm in width, 25 cm in length), which contained the mice as they ran at a constant velocity. The velocity selected for the assessment was fixed at 13 cm/s, a speed at which the *Ehmt2*^*+/del_1076-1079*^ animals were able to consistently ambulate throughout the experiment. A minimum of five fluent strides was recorded during each trial. A high-temporal-resolution video was recorded ( ~ 5 s) for each mouse, and video analysis was performed using the DigiGait Imaging and Analysis software ver. 16 (Mouse Specifics Inc., Framingham, MA, USA). The software automatically analyzed images to create a digital paw print and dynamic gait signals. Spatial and temporal gait parameters were measured from the generated signals. Data were averaged between the left and right paws in both the forelimbs and hindlimbs. To acclimatize to the apparatus, mice were trained on the DigiGait treadmill at speeds of 10 to 15 cm/s for a maximum of 1 min per mouse for 5 days prior to testing. The average of 3 days was used for subsequent calculations.

#### Grip strength

A grip strength meter was used to determine the maximal peak force (×g) exerted by the mouse forelimbs as well as all four limbs when the examiner tried to pull it out of a specially designed grid for mice (Bioseb, BIO-GS3 & BIO-GRIPGS, Bioseb). The maximal effort (g) was used as absolute force (×g) and was corrected for body mass (×g/×g). The experiment was conducted as a singly-anonymized trial. and a total of three measurements were applied to each mouse with a 10–15 min interval between measurements. The average of the 3 values was used for subsequent calculations.

#### Rotarod

The rotarod system was used to assess the sense of balance, motor learning, and motor coordination in the animals. Three trials per day for each mouse were recorded on a rod with an accelerating speed of rotation (4–40 rpm/5 min, RotaRod, TSE Systems) during two consecutive days. The average latency to fall was determined from three trials with 15 min intertrial intervals (ITI).

#### Echocardiography

Transthoracic echocardiography was performed using a high-resolution ultrasound imaging system (Vevo® F2, FUJIFILM VisualSonics) equipped with an ultra-high-frequency transducer (VisualSonics UHF46x, 46–20 MHz). Fifteen-week-old animals were anesthetized with 5% isoflurane for induction and positioned in the supine position on a temperature-controlled imaging platform maintained at 38 °C. During image acquisition, the isoflurane concentration was reduced to 1.5–2% to maintain stable and comparable heart rates (425 ± 50 bpm) and respiration rates.

Following the removal of chest hair and the application of pre-warmed ultrasound gel, two-dimensional B-mode cine loops were acquired. Parasternal long-axis (LAX) views were obtained to visualize the left ventricle (LV) from apex to base. Parasternal short-axis (SAX) views were subsequently recorded at the mid-ventricular level, typically at the level of the papillary muscles, to assess LV morphology and function.

After sonography, animals were returned to their cages, placed on a heating pad, and monitored until full recovery from anesthesia.

All measurements and calculations were made using AutoLV mode with the VevoLab software (FUJIFILM VisualSonics Inc.).

### Reporting summary

Further information on research design is available in the Nature Portfolio Reporting Summary linked to this article.

## Supplementary information

Supplementary Information

Description of Additional Supplementary Files

Supplementary Data 1-5

Supplementary Movie 1

Supplementary Movie 2

Reporting Summary

Transparent Peer Review file

## Source data

Source Data

## Data Availability

The pathogenic variants detected in our study have been submitted to ClinVar (https://www.ncbi.nlm.nih.gov/clinvar/), submission numbers SCV004231847, SCV006279706 to SCV006279710 and SCV007518740. Native MS data are available in ZENODO under 10.5281/zenodo.19630380. Mass spectrometry data from histone extracts are available in MassIVE repository under https://proteomecentral.proteomexchange.org/cgi/GetDataset?ID=PXD079241. Proteomic data from immunoprecipitation experiments are available in Pride (Project Number PXD076523). List of differentially methylated probes, histone modification data, thermostability data and kinetic data are provided in Supplementary Tables and Supplementary Data. Source data file is provided with the paper. RNA sequencing data are available at the European Genome-phenome Archive under accession https://ega-archive.org/studies/EGAS50000001637. Because of the Personal Information Protection Law, individual participant DNA sequencing data beyond that reported in this article are under regulation by the individual institutional review boards. Datasets for episignature analyses used in this study that are available publicly are previously described^20^. The individual genomic and epigenomic or any other personally identifiable data for other samples in the EpiSign Knowledge Database (EKD) are prohibited from deposition in publicly accessible databases due to institutional and ethics restrictions. Specifically, these include data and samples submitted from external institutions to London Health Sciences EKD that are subject to Institutional Material and Data Transfer agreements, data submitted to London Health Sciences for episignature assessment under Research Services Agreements, and research study cohorts under Institutional Research Ethics Approval (Western University REB 106302; and REB 116108). Some of the software packages used in this study are publicly available as described in the Materials and Methods. EpiSign™ is a commercial software and is not publicly available. Source data are provided with this paper.
