# Supplementary material for: De novo EHMT2 variants cause an autosomal dominant EHMT2-related Kleefstra syndrome via loss of G9a methyltransferase activity
Source: Nat Commun. 2026 Jul 1;17:8203. doi: 10.1038/s41467-026-74987-w (PMC13462943; doi:10.1038/s41467-026-74987-w)
Supplement: Supplementary file 1 [file 41467_2026_74987_MOESM1_ESM.pdf]

De novo EHMT2 variants cause an autosomal dominant *EHMT2*-related Kleefstra syndrome via loss of G9a methyltransferase activity

Supplementary Information

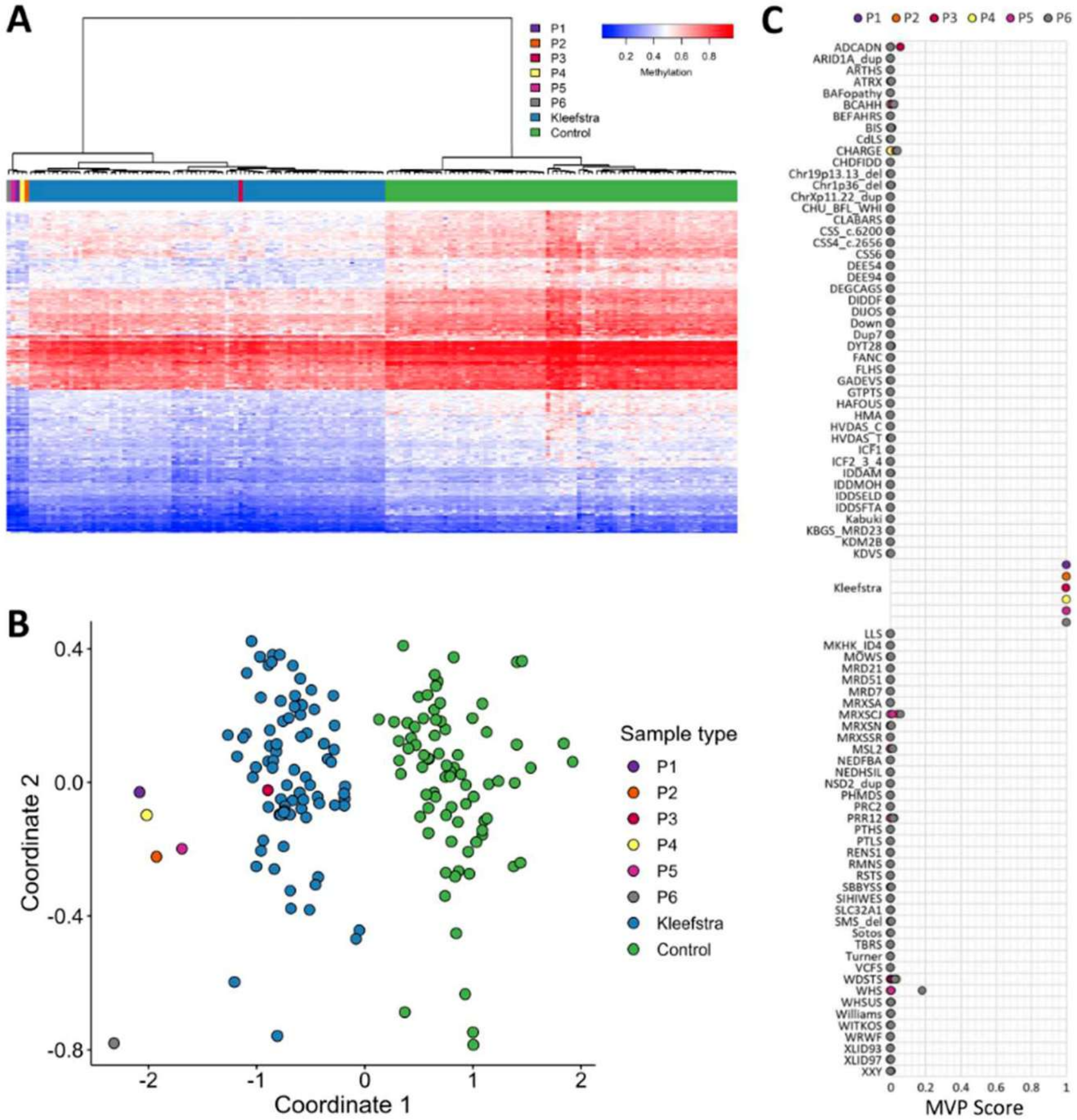

Supplementary Figure 1. EpiSign (DNA methylation) analysis of peripheral blood from patients with EHMT2 variants.

(A) Hierarchical clustering and (B) multidimensional scaling plots indicate the patients (purple, orange, red, yellow, pink and grey) have a DNA methylation profile similar to subjects with a confirmed Kleefstra episignature (blue) and distinct from controls (green). (C) MVP score, a multi-class supervised classification system capable of discerning between multiple episignatures by generating a probability score for each episignature. The elevated score for Kleefstra shows an episignature similar to reference patients with pathogenic variants in EHMT1.

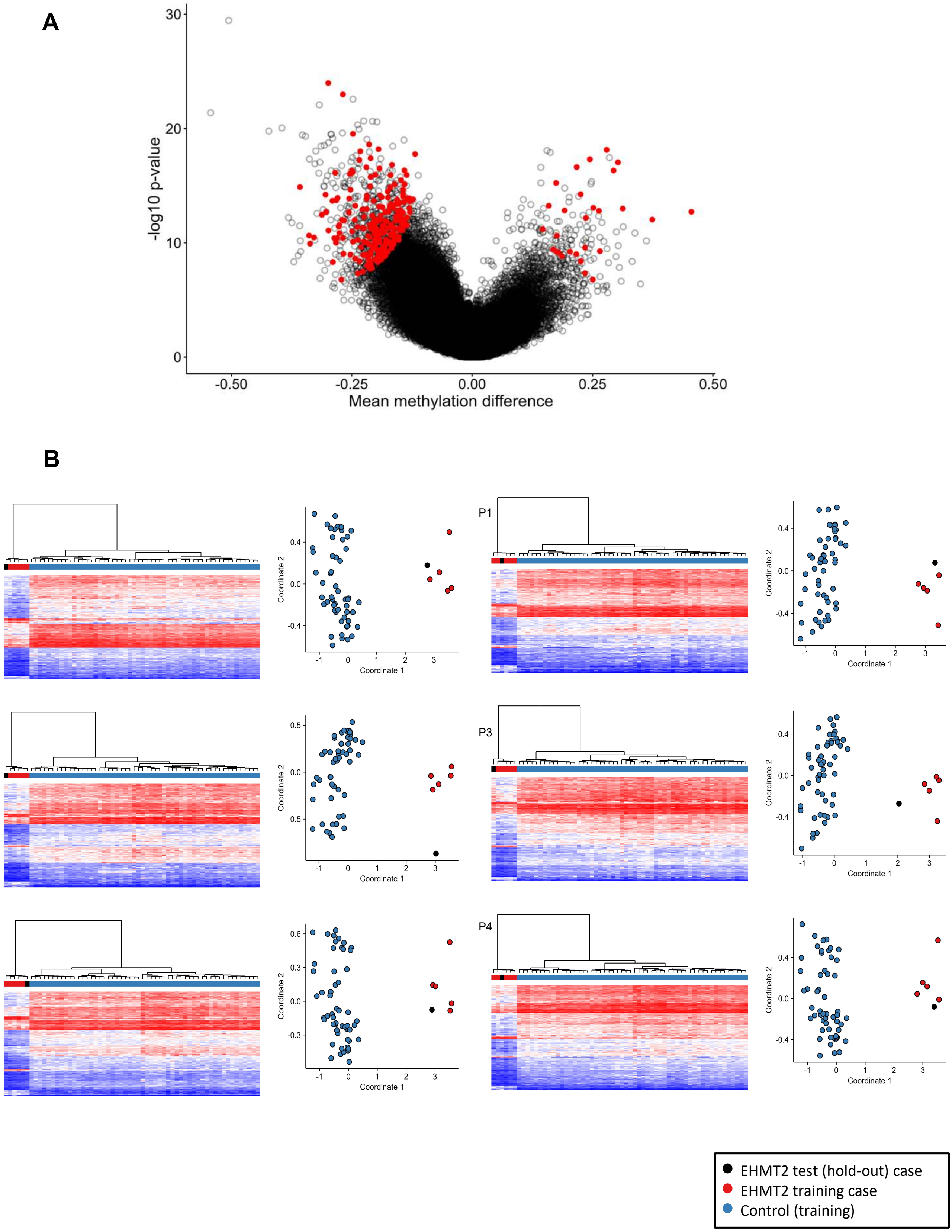

Supplementary Figure 2. A. Visualization of mean methylation differences and p-values for the episignature probes. The volcano plot displays all episignature probes, with mean methylation differences on the x-axis and –log10 p values on the y-axis. Probes selected through the multi-step ranking procedure for the EHMT2 episignature are highlighted in red.

B. Unsupervised clustering results for leave-one-out cross-validation on PTBP1 discovery cases. In each cross-validation iteration, a single PTBP1 start-loss case was held-out from the discovery data as a test case and a sub-signature was generated using the same feature selection parameters of the identified signature. Reproducibility and robustness of the episignature is validated by consistent clustering of the hold-out case (black) with the remaining discovery cases (red) instead of the matched controls (blue).

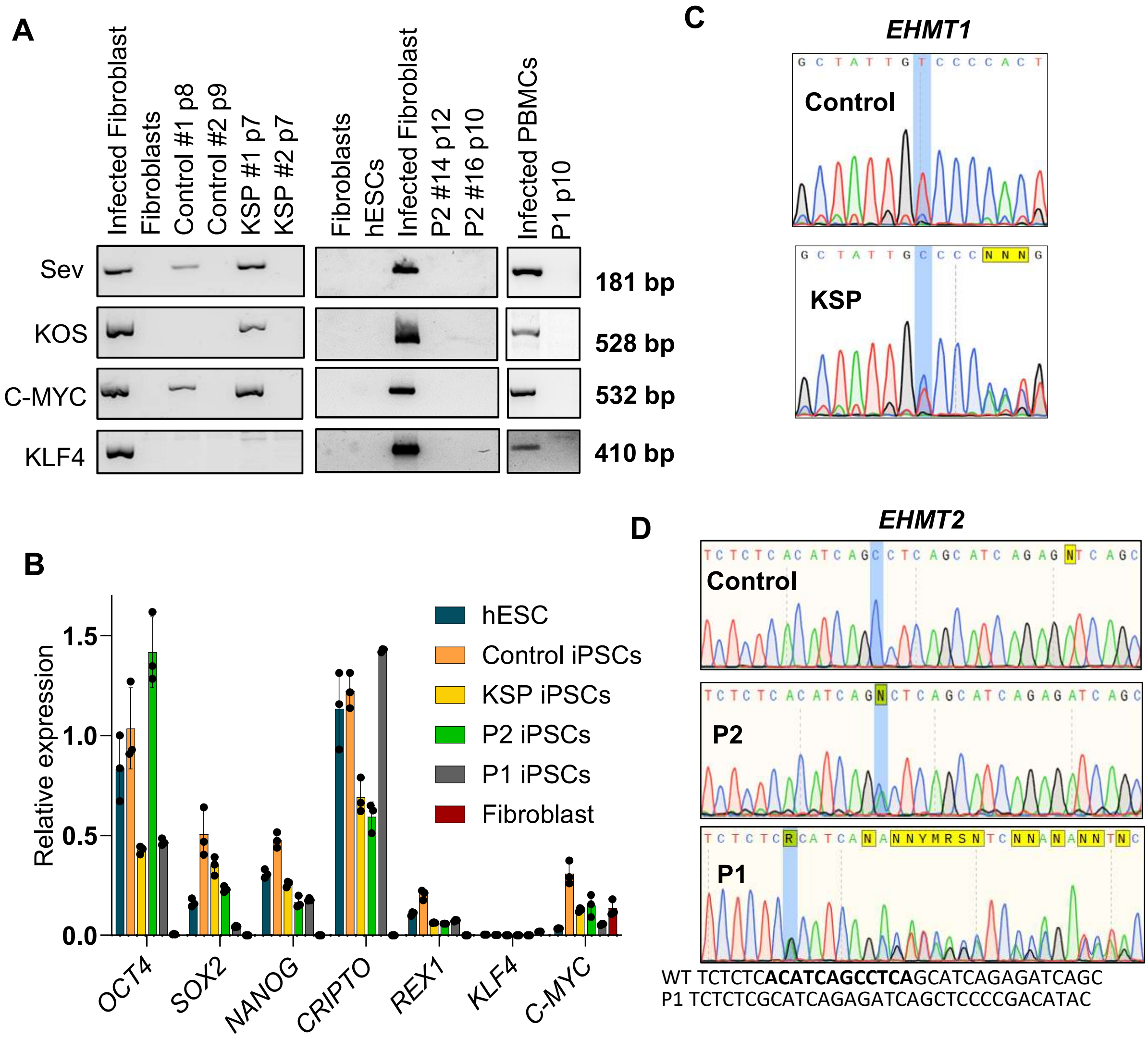

Supplementary Figure 3. Characterization of iPSCs lines. A. Transgenes expression in the different lines at the indicated passages. Transgene-free clones Control #2, KSP #2 and P2 #16 were selected for subsequent analysis. B. Pluripotency markers expression in the indicated cell lines obtained by qPCR. Three replicates are shown. The human embryonic stem cell hESC (AND-2) is included as a positive control. C. Sanger sequencing of EHMT1 mutation in control and KSP iPSC lines D. Sanger sequencing of EHMT2 mutations in control and P1 and P2 iPSC lines. For patient P1 missing nucleotides are highlighted in bold.

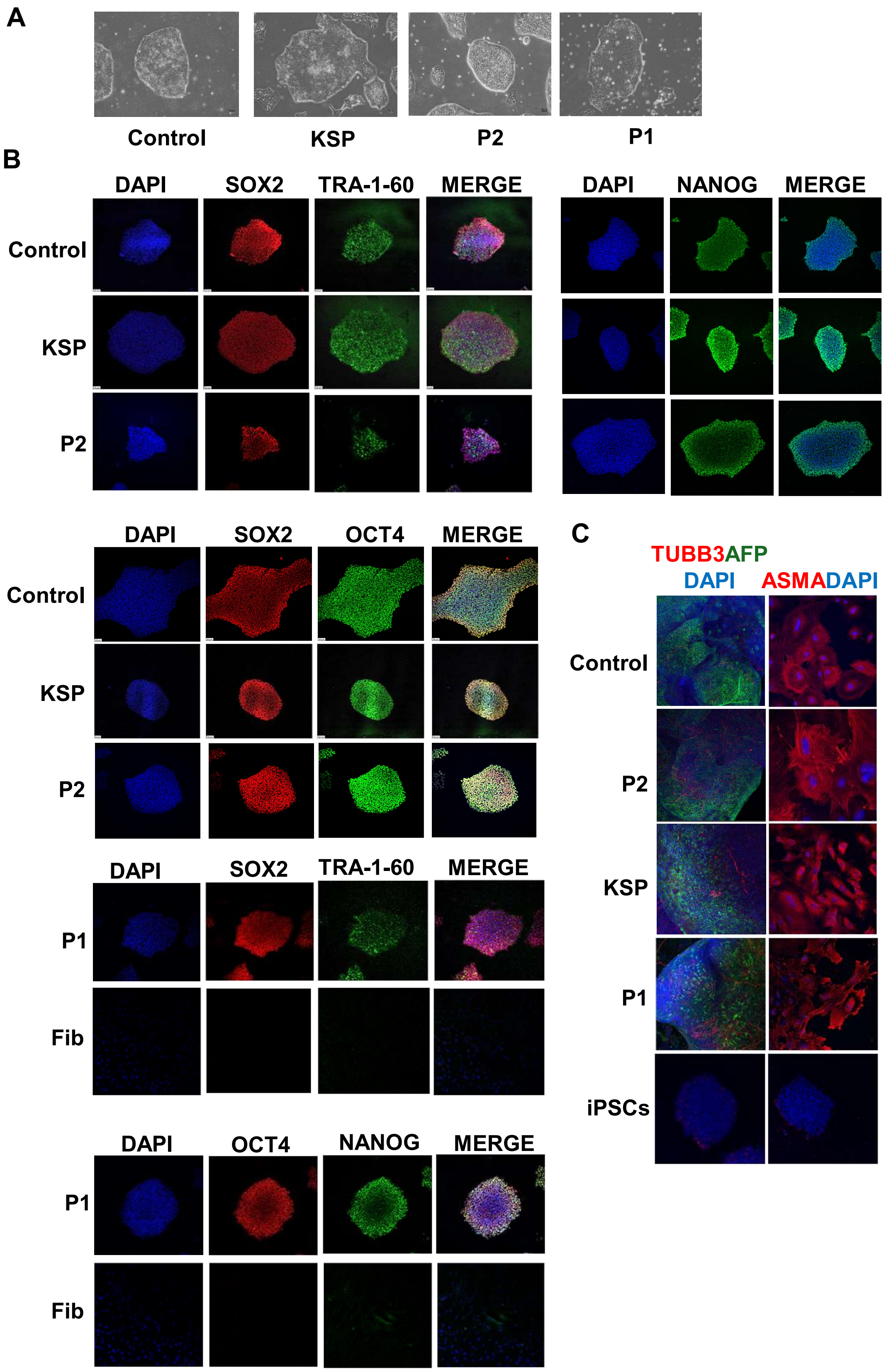

Supplementary Figure 4. Characterization of iPSCs lines by immunochemistry. A. Phase contrast pictures of growing cell lines. B. Immunohistochemistry of pluripotency markers in the indicated cell lines. All pictures correspond to iPSCs except negative controls performed in fibroblasts (Fib) C. Detection of differentiation markers in EBs at day 20 of in vitro differentiation. Negative controls were performed in undifferentiated iPSCs.

A

Histone PTM	Log2 P2/C	Log2 KSP/C
H1.4: k25me2	-1.87	0.28
H3: k79ac	-1.74	-0.14
H3R2un: q5me1	-0.73	-0.50
H3: k9me2	-0.71	-0.53
H1.4: k25me1	-0.66	-1.01
H3: k9me3	-0.60	-0.55
H3: k79un	-0.60	-0.30
H3.3: k27ac	-0.53	0.22
H3.3: k36un	-0.48	-0.56
H4: k20un	-0.38	0.07
H3: q55me1	-0.23	0.38
H3.1: k27un	-0.21	0.21
H3: k9me1	-0.18	-0.23
H3.1: k36un	-0.17	-0.28
H3: k79me1	-0.14	-0.20
H4: k20me1	-0.14	0.03
H3: k14un	-0.12	-0.08
H3.3: k36me1	-0.11	-0.56
H3.3: k36me3	-0.11	0.19
H3R2un: k4me1	-0.07	0.08
H4: k16un	-0.06	-0.05
H3: r49un	-0.05	-0.06
H3: k23un	-0.05	-0.04
H3.3: k27me1	-0.04	-0.12
H3: k122ac	-0.04	1.54
H4: k8un	-0.04	-0.04
H2A: k5ac	-0.04	0.04
H3: r42un	-0.04	-0.03
H3.1: k27me2	-0.03	-0.22
H4: k12un	-0.03	-0.03
H4: k5un	-0.02	-0.01
H3: k18un	-0.02	-0.03
H3R2un: k4me2	-0.01	-0.07
H3.1: k27me1	-0.01	0.08
H3: k56un	0.00	0.00
H2A: k9un	0.00	0.00
H2A3: k15un	0.00	0.00
H3: q19un	0.00	0.00
H2A: k36un	0.00	0.00
H3: k64un	0.00	0.00
H2A1: k13un	0.00	0.00
H2A1: k15un	0.00	0.00
H2A3: k13un	0.00	0.00
H3: k122un	0.00	0.00
H3: q55un	0.00	0.00
H2A: k5un	0.00	0.00
H3R2un: q5un	0.01	0.00
H3R2un: k4un	0.01	-0.01
H3.3: k27me3	0.01	-0.10
H1.4: k25un	0.02	0.03
H4: k20ac	0.04	0.05
H3.1: k36me3	0.04	0.26
H3.1: k36me2	0.06	0.20
H3.3: k27un	0.07	0.19
H3.3: k27me2	0.09	-0.16
H4: k16ac	0.13	0.11
H3.1: k27ac	0.13	0.05
H4: k20me2	0.16	-0.20
H3.3: k36ac	0.16	0.26
H3: k79me2	0.22	0.14
H3: k18ac	0.23	0.42
H3: k18me1	0.24	-0.14
H3: k23ac	0.24	0.24
H3.3: k36me2	0.25	0.15
H2A3: k13ac	0.27	0.32
H3: k14ac	0.28	0.19
H3.1: k36me1	0.28	0.02
H3: k9un	0.33	0.30
H3.1: k27me3	0.38	0.13
H2A: k9ac	0.41	0.41
H4: k5ac	0.46	0.28
H3: q19me1	0.48	-0.07
H3: k9ac	0.50	0.70
H3: k56me1	0.50	0.85
H3: k23me1	0.50	0.77
H2A: k36ac	0.59	-0.51
H3.1: k36ac	0.63	1.36
H4: k8ac	0.63	0.62
H2A3: k15ac	0.78	2.22
H2A1: k15ac	0.83	0.41
H4: k12ac	0.86	0.88
H4: k20me3	0.90	0.72
H3R2un: k4me3	0.93	0.45
H3R2un: k4ac	0.95	-0.33
H2A1: k13ac	0.99	0.28
H1.4: k25ac	1.09	1.15
H3: r49me2	1.10	1.22
H3: r42me2	1.42	1.33
H1.4: k25me3	1.60	0.51
H3: k56ac	2.37	1.73
H3: k64ac	2.46	2.73
H3: k79me3	2.97	2.27

B

Histone PTM	Log2 FIB/iPSCs
H3: q19me1	-4.56
H3: k64ac	-4.21
H3: q55me1	-3.66
H4: k20me3	-3.62
H2A: k9ac	-3.53
H1.4: k25me3	-3.51
H4: k12ac	-3.16
H4: k8ac	-2.83
H3.1: k27ac	-2.78
H3R2un: k4ac	-2.52
H4: k20me1	-1.80
H3: k18ac	-1.80
H4: k20me2	-1.72
H3: k23ac	-1.65
H2A: k5ac	-1.63
H4: k5ac	-1.52
H3.1: k36me2	-1.41
H4: k16ac	-1.17
H3.1: k27un	-1.09
H2A3: k15ac	-0.87
H3: k14ac	-0.75
H3: k9ac	-0.74
H3.3: k27ac	-0.73
H3: k23me1	-0.59
H3.3: k36me2	-0.59
H2A: k36ac	-0.51
H3: k79un	-0.51
H3.1: k27me1	-0.45
H3: k9un	-0.33
H3.3: k27un	-0.31
H3R2un: k4me1	-0.27
H3R2un: k4me2	-0.20
H3.3: k27me1	-0.16
H3: k9me2	0.19
H3: k18me1	0.20
H2A: k5un	0.23
H3: k18un	0.25
H3: k79me1	0.29
H4: k12un	0.31
H3.3: k36me1	0.35
H3.1: k36me1	0.39
H3.1: k36me3	0.41
H3: k9me3	0.41
H3: k14un	0.42
H3.3: k36un	0.44
H3: k9me1	0.46
H3.3: k36me3	0.46
H4: k8un	0.49
H3: k23un	0.64
H3: r49me2	0.68
H4: k16un	1.06
H3.1: k36ac	1.08
H3.3: k27me2	1.08
H3: r42me2	1.17
H1.4: k25me1	1.32
H3.3: k27me3	1.37
H4: k20un	1.54
H3.1: k36un	1.64
H3.3: k36ac	1.65
H3: k56me1	1.68
H3.1: k27me2	1.88
H3: k79me2	2.96
H3.1: k27me3	3.36
H4: k20ac	3.66
H1.4: k25me2	5.12
H3: k79me3	5.16

Supplementary Figure 5. Fold change of histone modifications determined by mass spectrometry. A. Log2 of fold change in the indicated modifications in P2 or KSP fibroblasts relative to healthy control fibroblasts. B. Log2 of fold change in the indicated modifications in control fibroblasts relative to control iPSCs. Modifications that significantly change at least 10% in abundance at a p-value<0.05 are shown.

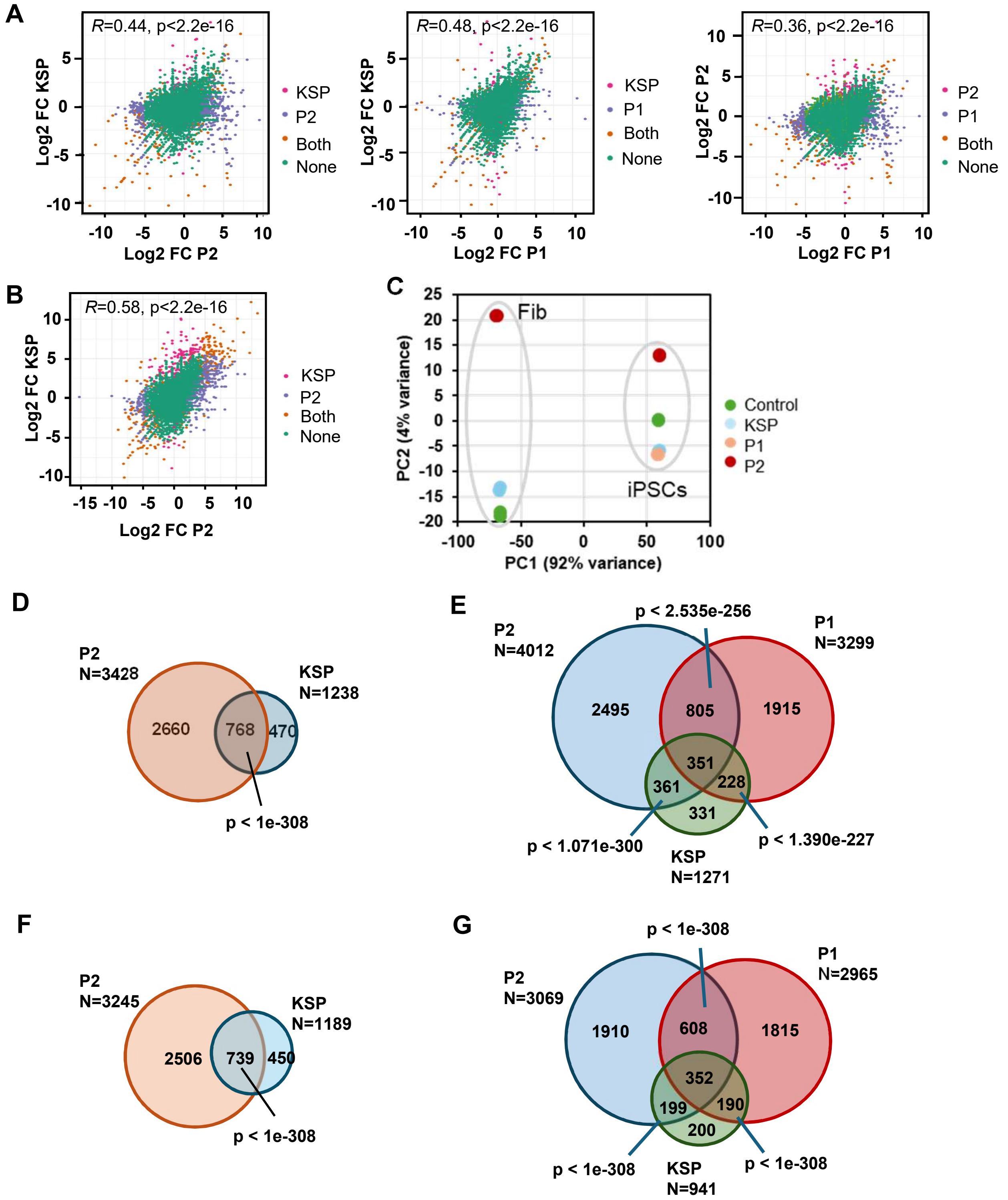

Supplementary Figure 6 . Transcriptomic analysis of cell lines derived from a healthy control, a Kleefstra Syndrome patient (KSP) and patients P1 and P2 carrying EHMT2 variants. A. Scatter plot showing correlation between expression fold change of all genes in patients compared to control iPSCs. Pearson's correlation coefficient (R) and p-value is indicated. Genes changing expression at an adjusted p-value<0.05 only in the indicated patients only (pink or purple), in both (orange) or not significantly changing expression (green) are shown. B. Scatter plot showing correlation between expression fold change of all genes in the patients' compared to control fibroblasts. Pearson's correlation coefficient (R) and p-value is indicated. Genes changing expression at an adjusted p-value<0.05 only in KSP (pink), only in P2 (purple), in both (orange) or not significantly changing expression (green) are shown. C. Principal component analysis of healthy and patient's fibroblasts and iPSCs. D. Overlap of differentially upregulated genes compared to control in patient's fibroblasts. P-value shows the significance of the overlap. E. Overlap of differentially upregulated genes compared to control in patient's iPSCs. P-value shows the significance of the overlap. F. Overlap of differentially downregulated genes compared to control in patient's fibroblasts. P-value shows the significance of the overlap. G. Overlap of differentially downregulated genes compared to control in patient's iPSCs. P-value shows the significance of the overlap.

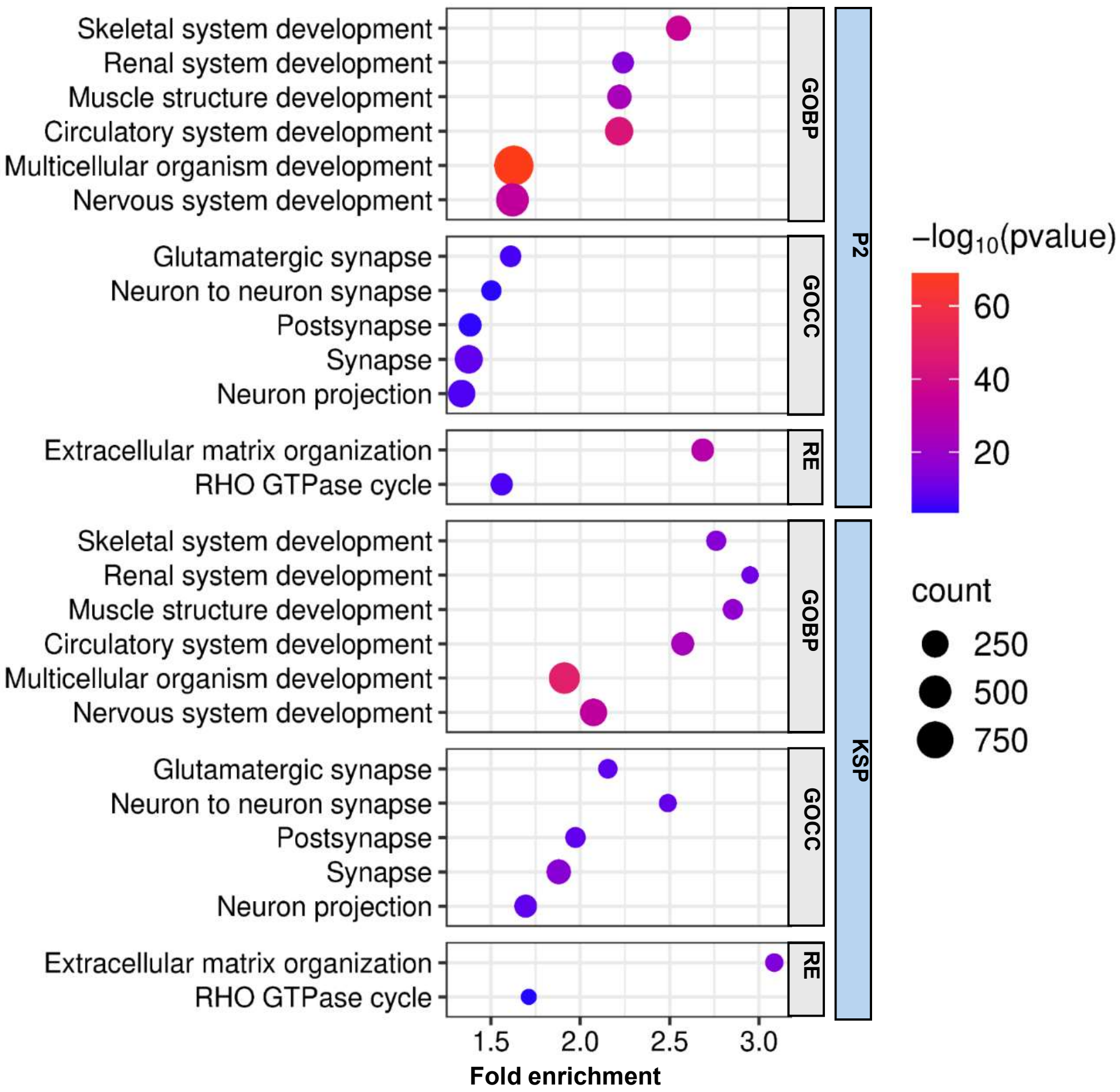

Supplementary Figure 7. DAVID enrichments of GOBP, GOCC and Reactome (RE) terms in genes upregulated in KSP or P2 fibroblasts compared to healthy control fibroblasts.

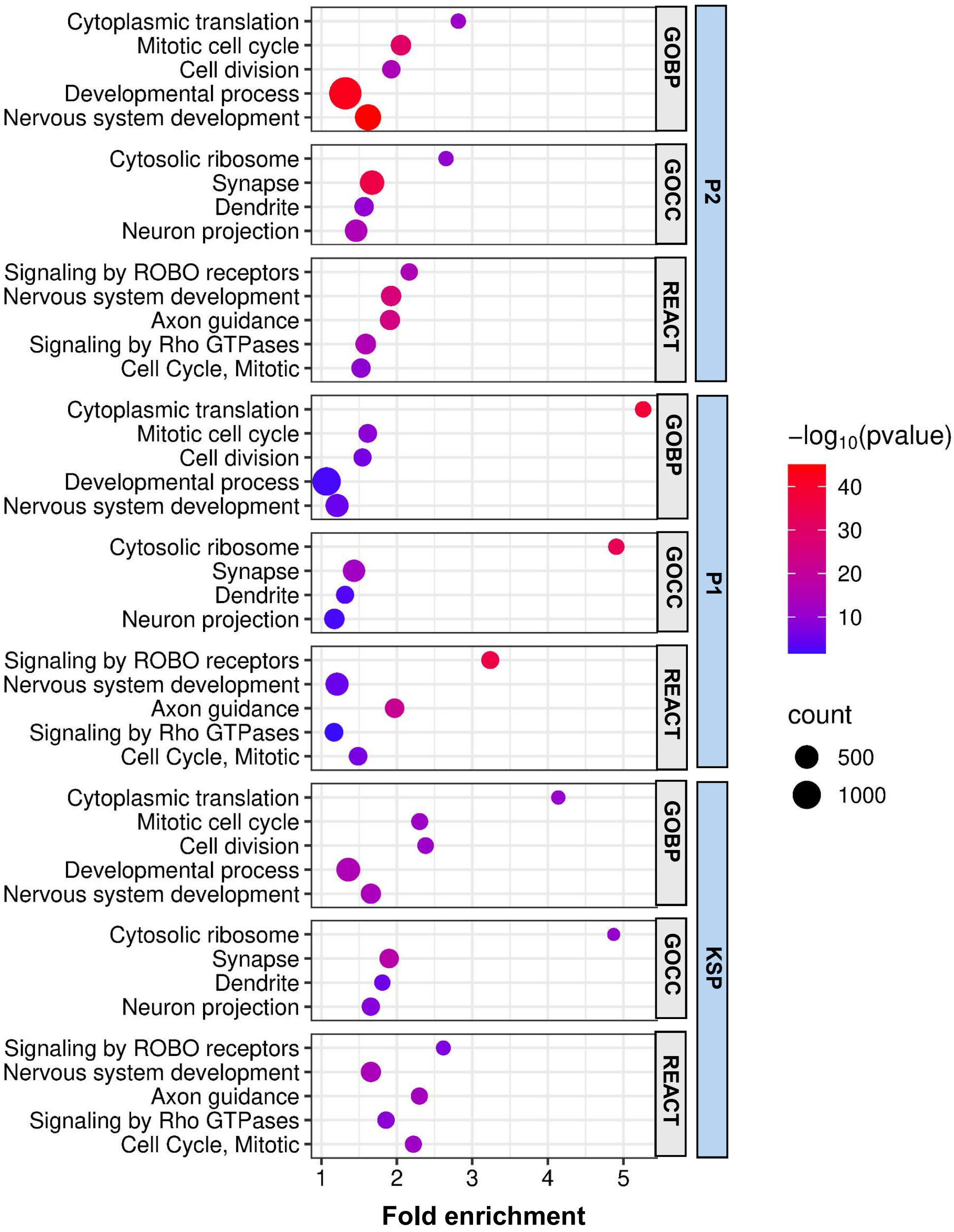

Supplementary Figure 8 . DAVID enrichments of GOBP, GOCC and Reactome (REACT) terms in genes upregulated in P2, P1 or KSP iPSCs compared to healthy control iPSCs.

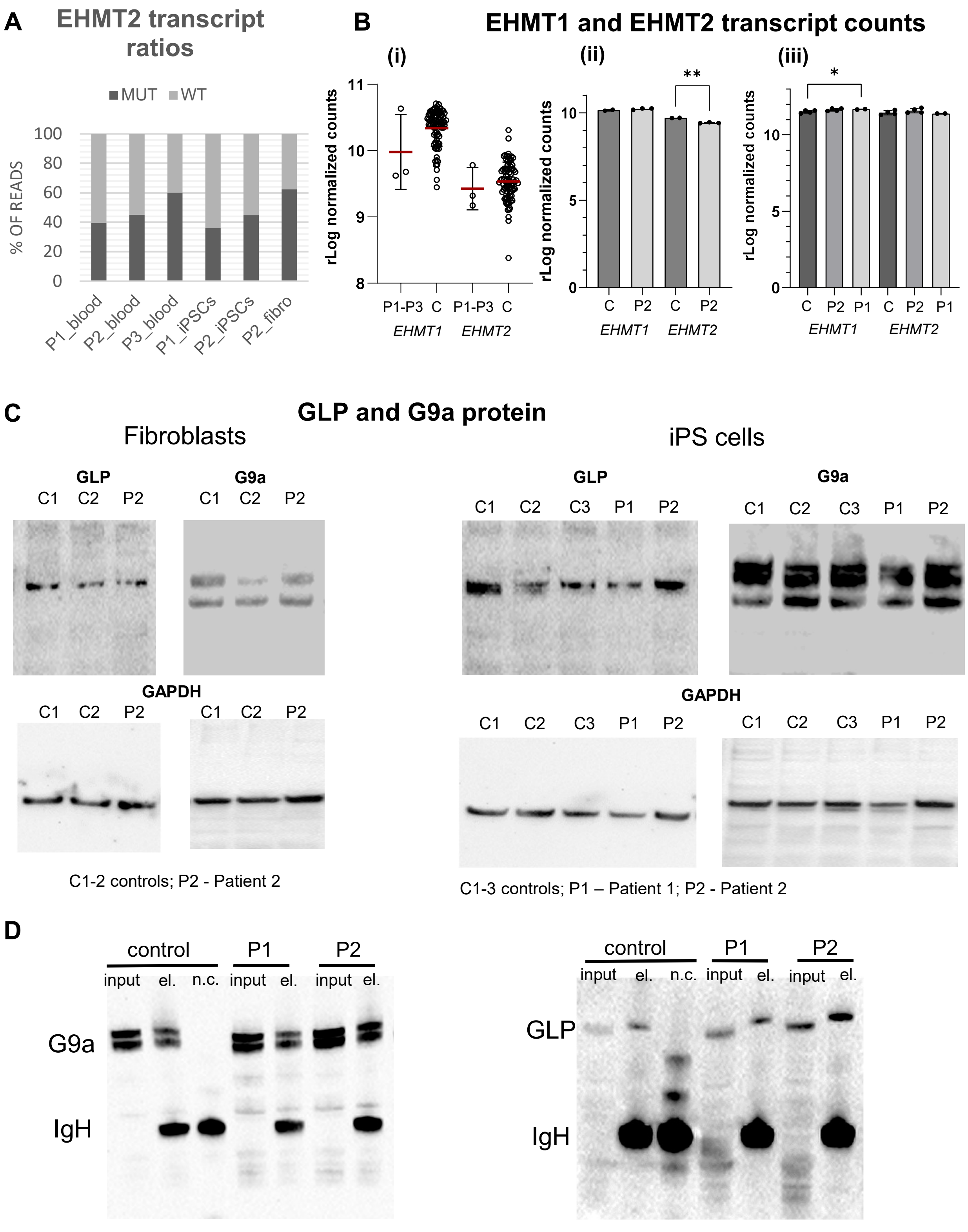

Supplementary Figure 9.

A. Percentage of mutant (MUT) and wild type (WT) reads of *EHMT2* transcript detected by RNA-seq from leukocytes of P1, P2 and P3, iPSCs from P1 and P2 and fibroblasts from P2.
B. (i) mRNA levels of EHMT1 and EHMT2 in the blood of patients P1, P2 and P3 and 83 control samples.
(ii) mRNA levels of EHMT1 and in fibroblasts derived from patient P2 (n=3) and control fibroblasts (C) n=2.
(iii) mRNA levels of EHMT1 and in iPSCs derived from patient P2 (n=4), P1 (n=2) and control fibroblasts (n=4).
All plots show means with standard deviation. Significant differences by t-test are indicated. * p<0.05, **p<0.005
C. Western blot analysis of whole- cell extracts of patient-derived fibroblasts and iPSCs to determine the amount of GLP and G9a protein. GAPDH protein was used as a housekeeping reference.
D. Western blot analysis of G9a immunoprecipitation in iPSCs. “el.” and “n.c.” mean elution and negative control, respectively.

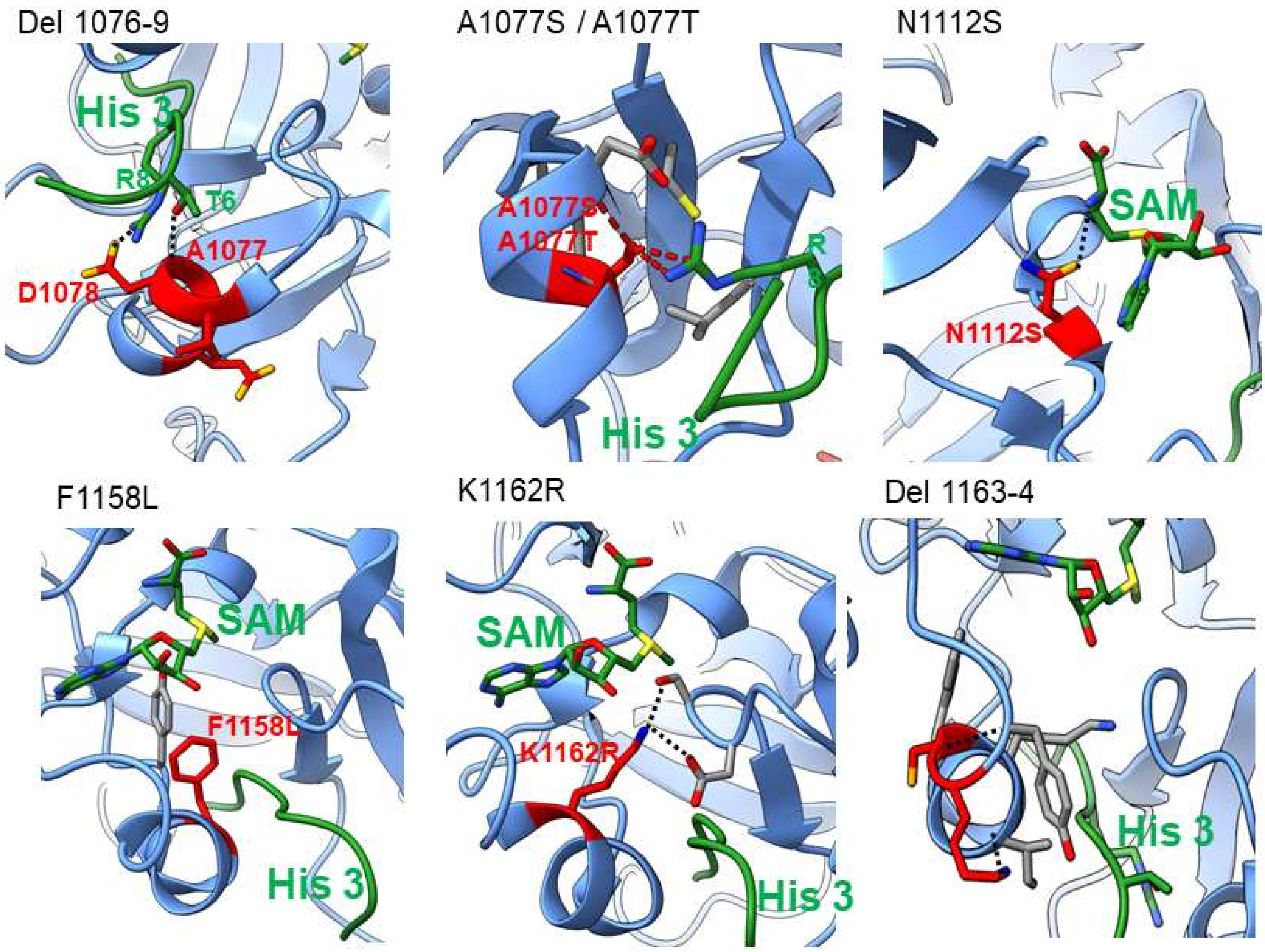

Supplementary Figure 10. Structural analyses of G9a mutants using available crystal structure of the G9a SET-domain in complex with SAM and histone 3 peptide (PDB ID 5JIN). Mutated residues are highlighted by red color; lost interactions and sterical clashes are depicted as dashed black and red lines, respectively.

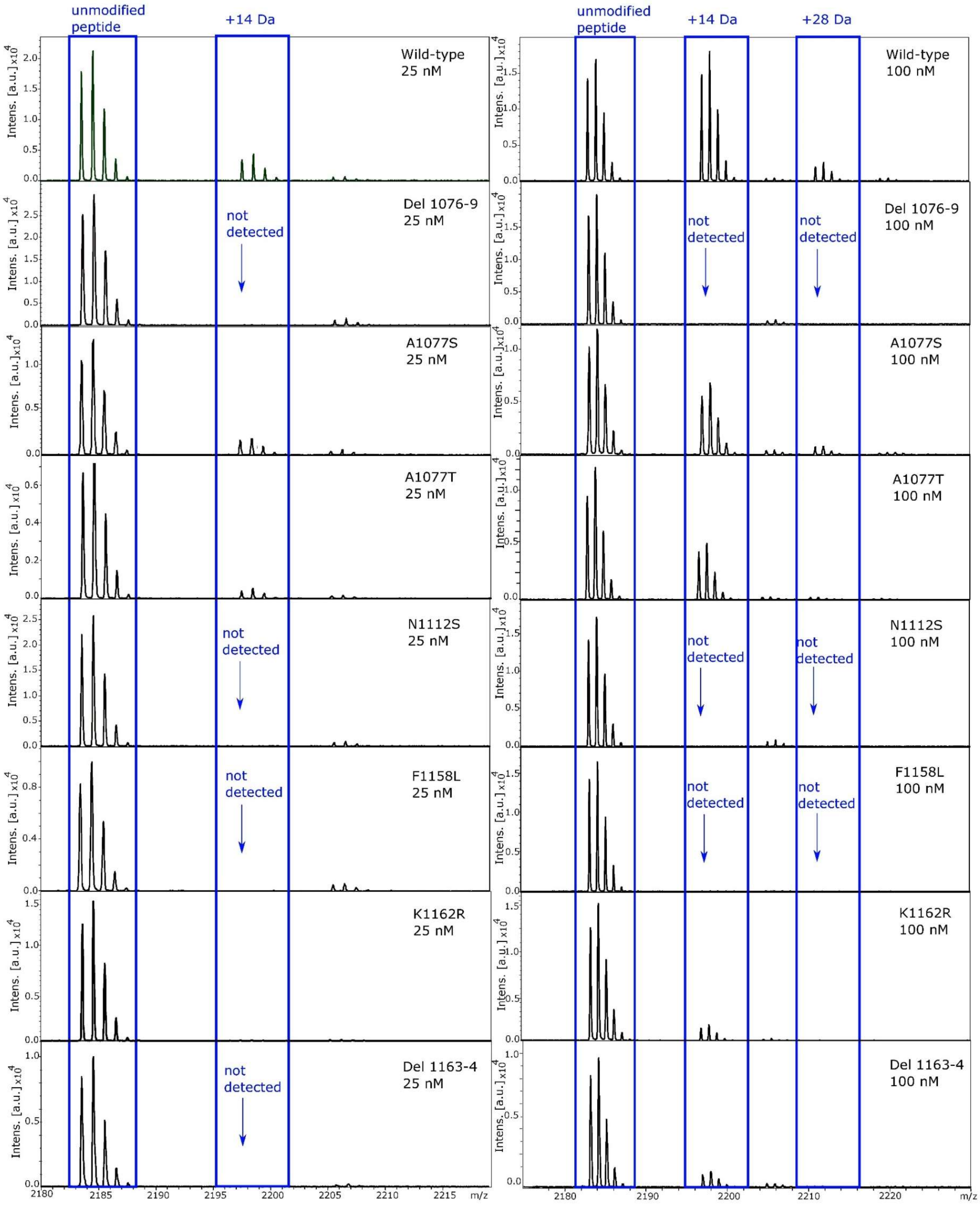

Supplementary Figure 11. Methyltransferase activity of G9a variants with histone 3 peptide (aa 1-20) assessed by MALDI-TOF MS. Reactions were performed for 30 minutes at 30 °C with two different concentrations of the enzyme (25 and 100 nM). Methylation of histones 3 peptides causes an increase in molecular mass by 14 (monomethylation) and 28 Da (dimethylation). Series of histone 3 peptides are highlighted by blue boxes. The mutants exhibited undetectable or decreased residual activity compared to the wild-type protein.

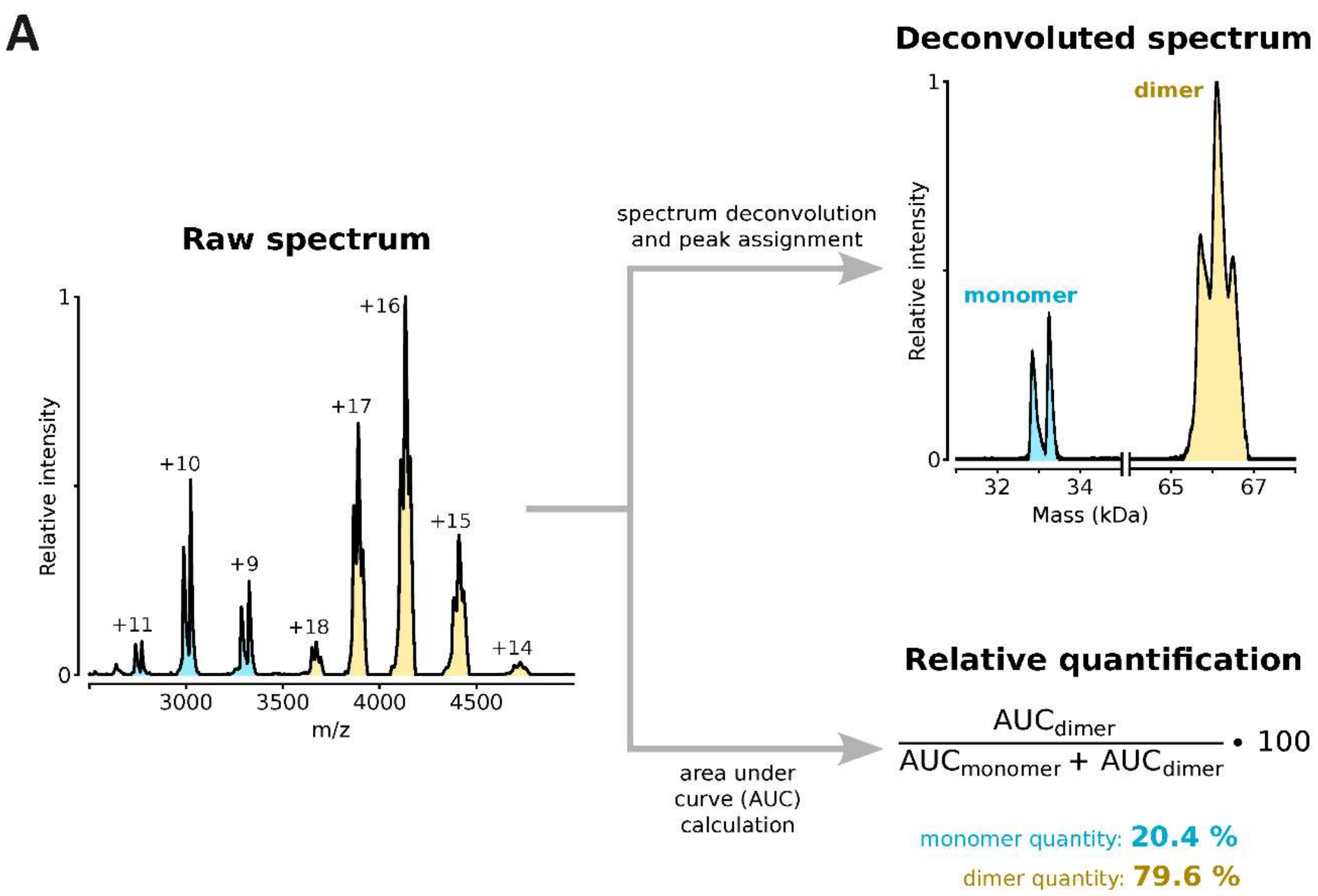

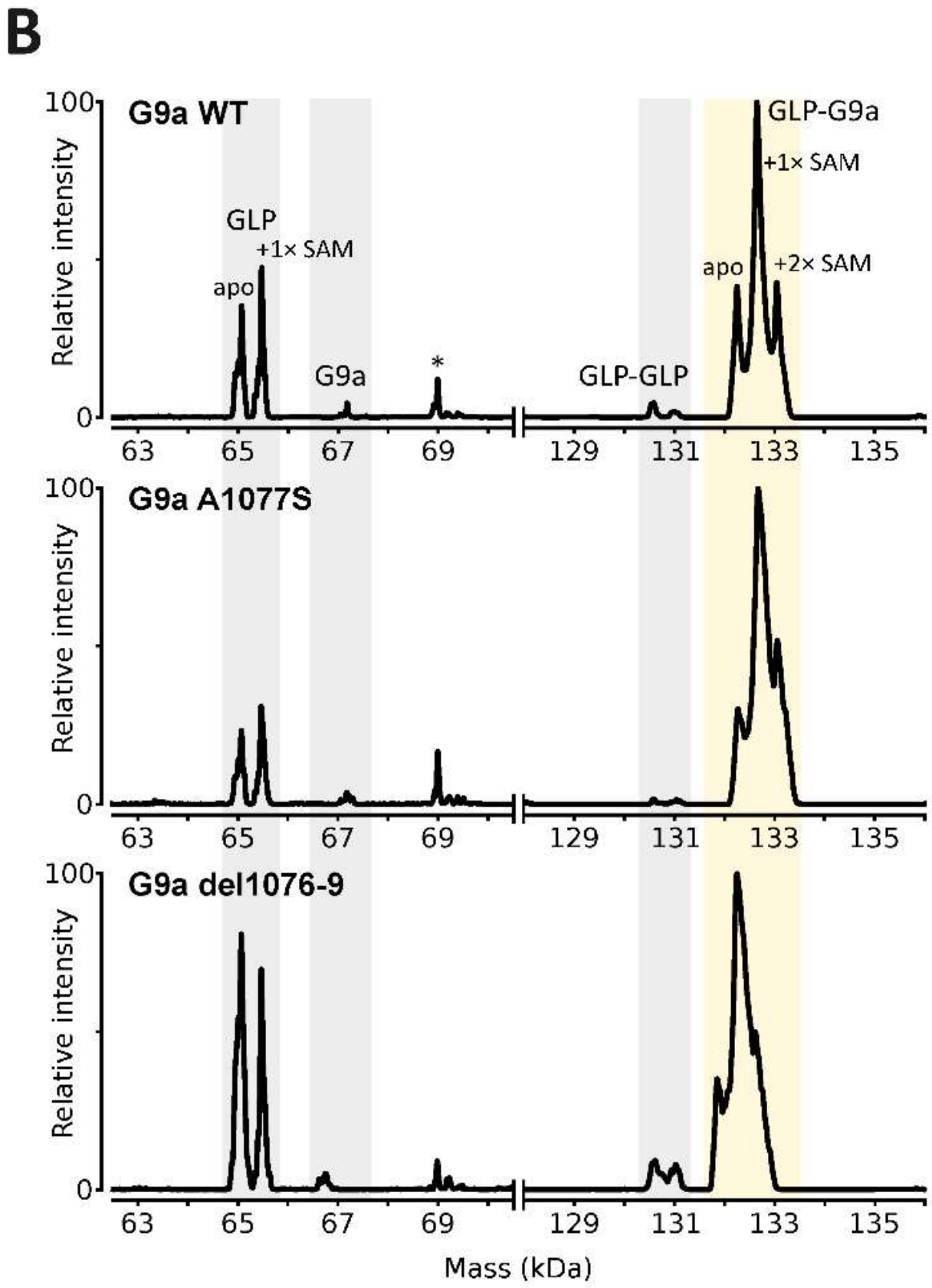

G9a variant	Relative amount of GLP-G9a heterodimer (%)
WT	80.7
A1077S	87.3
del1076-9	65.5

Supplementary Figure 12. Mass spectrometric analysis of G9a and GLP dimerization. **A** Evaluation of the monomer-dimer distribution in G9a and GLP variants using nESI-MS. The procedure is illustrated using representative data from methyltransferase domain of the G9a wild-type. Initially, raw spectra were deconvoluted to facilitate peak assignment. Subsequently, relative quantities of monomeric and dimeric forms were calculated from the areas under curve (AUC) of the corresponding peaks in raw MS spectra. B. Deconvoluted nMS spectra of GLP-G9a heterodimer, comprising ankyrin repeat region and methyltransferase domain, with variants in G9a. Yellow area highlights properly assembled heterodimer. Peak denoted with asterisk was not assigned and likely represents protein contaminant from recombinant expression.

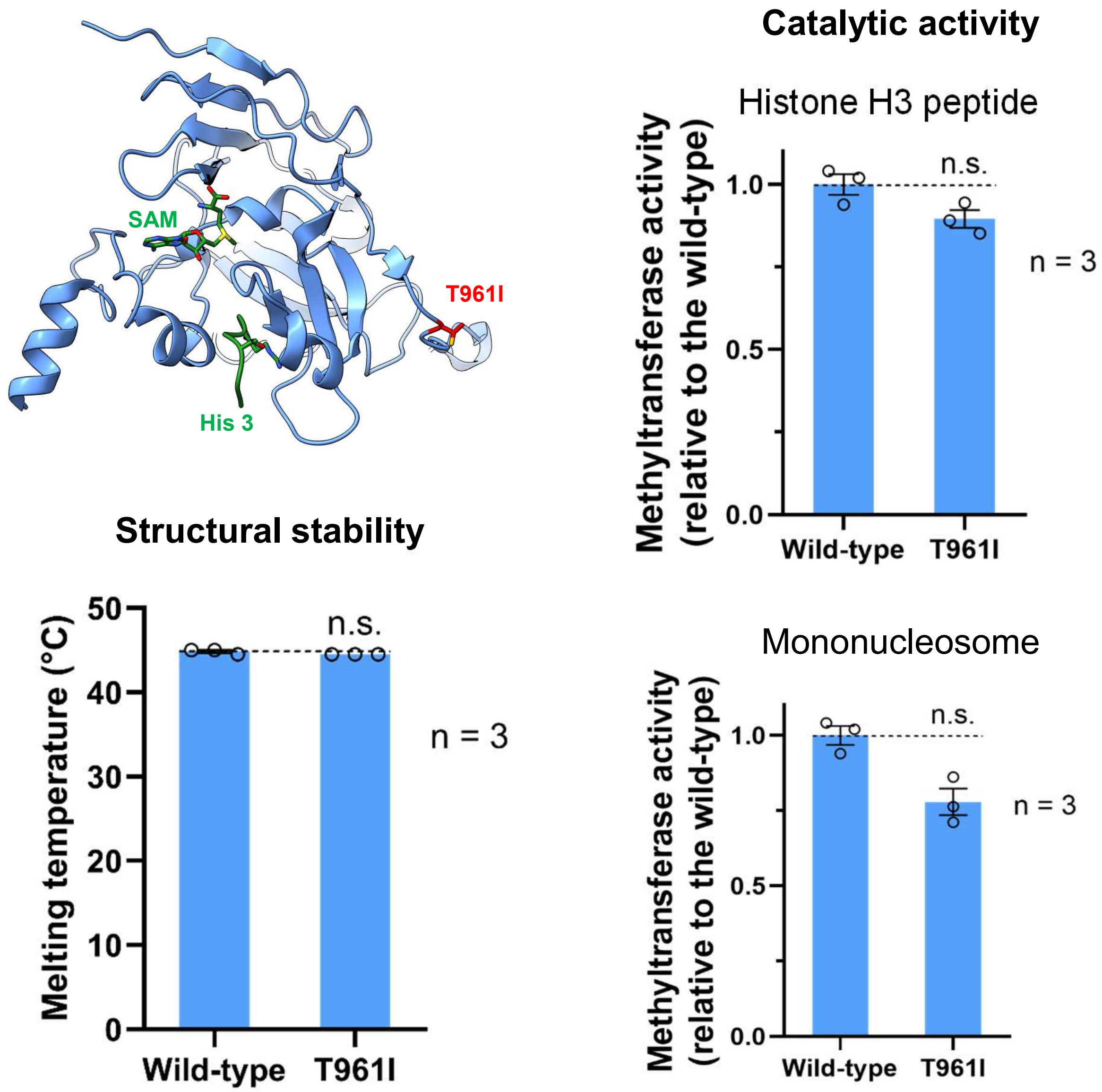

Supplementary Figure 13. Structural topology, catalytic activity and structural stability of the variant T961I with unknown clinical significance. Values represent means with standard errors from three measurements. Statistical significance was determined using two-tailed Student's t-test ($p < 0.01$).

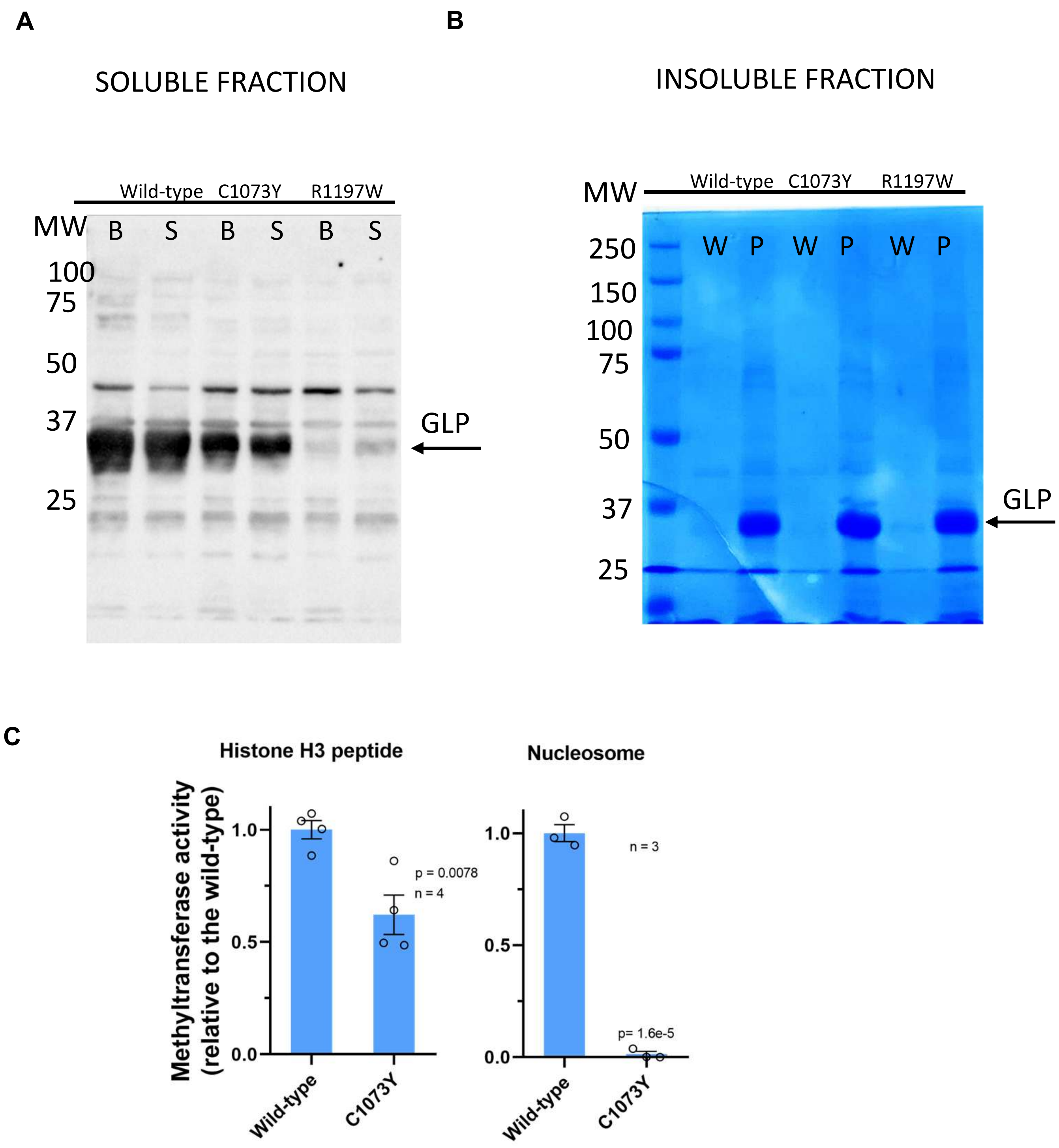

Supplementary Figure 14. Production of GLP methyltransferase domain with histidine tag in *E.coli* and its distribution in soluble and insoluble fraction and its catalytic activity. A. Bacterial lysates were prepared by sonication (S) or extraction using BugBuster (B) followed by centrifugation. The soluble fraction represents supernant (10 µg of total protein) analyzed by western blotting followed by immunodetection against histidine tag. B. The insoluble fraction represents pellet after centrifugation analyzed by Coomassie Blue-stained SDS-PAGE (W – wash of insoluble fraction; P – pellet solubilized by heating) (C) Methylation activity of purified GLP variants with histone H3 peptide and mononucleosome as substrates studied by quantitative bioluminescence-based assay (Mtase-Glo by Promega). The values represent the means of relative activity to the wild-type enzyme with standard errors. Statistical significance was determined using two-tailed Student's t-test ($p < 0.01$).

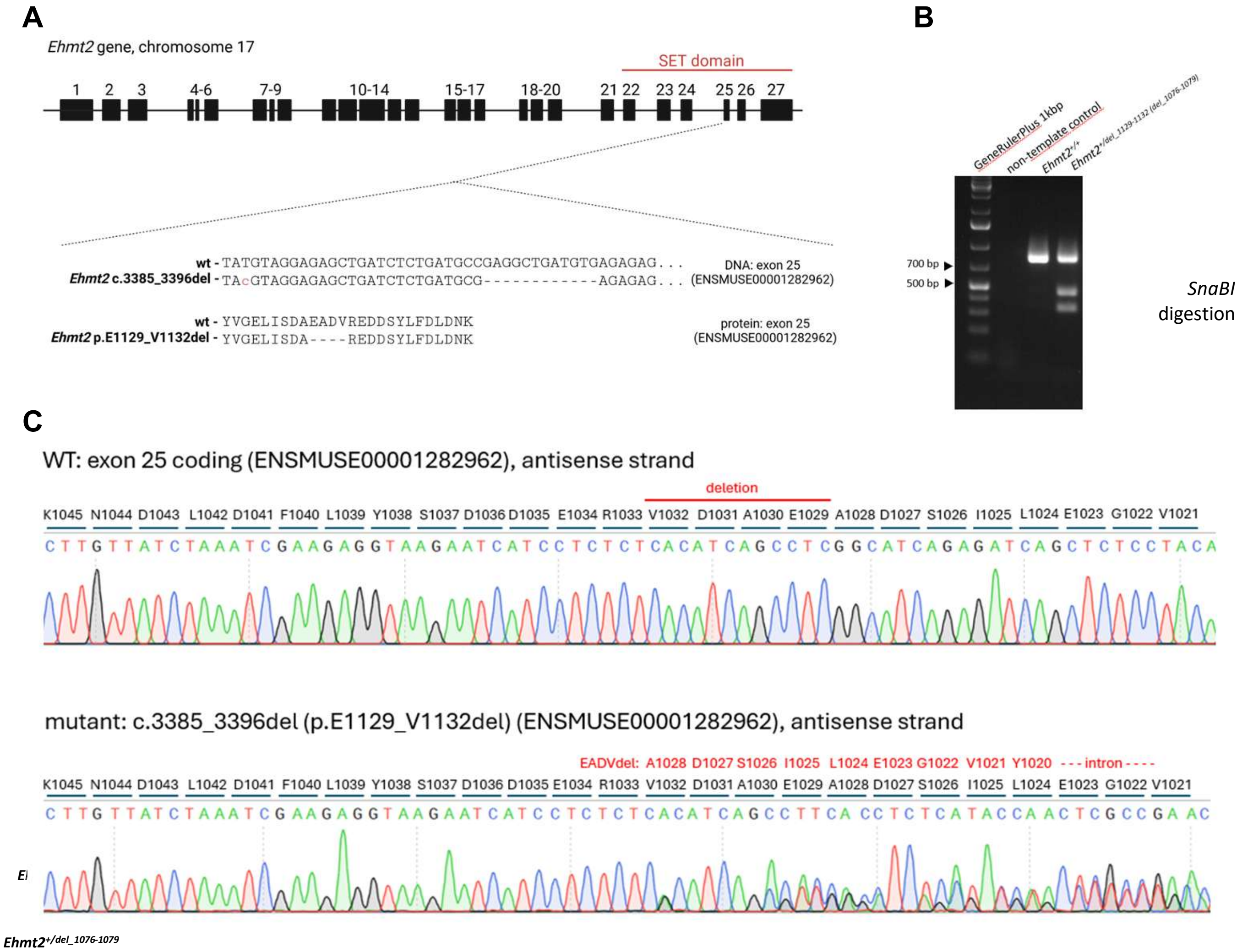

Supplementary Figure 15: Generation of a mouse model. A. Localization and sequence characteristics of the *Ehmt2* c.3385_G3396del (p.E1129_V1132del, [C57BL/6NCrl-Ehmt2^{em1Ccpcz}/Ph) variant that was designed to mimic the c.3225_3236del (p.Glu1076_Val1079del) *EHMT2* variant detected in patient 1.

B. Genotyping strategy using *SnaBI* digestion.

C. Example of direct Sanger sequencing of the critical targeted *Ehmt2* exon 25 in an *Ehmt2*$^{+/+}$ and *Ehmt2*$^{+/del\_1076\text{-}1079}$ mouse. Frame-shift triggered by the deletion of the 12 nucleotides can be seen in the sequence from the *Ehmt2*$^{+/del\_1076\text{-}1079}$ mouse.

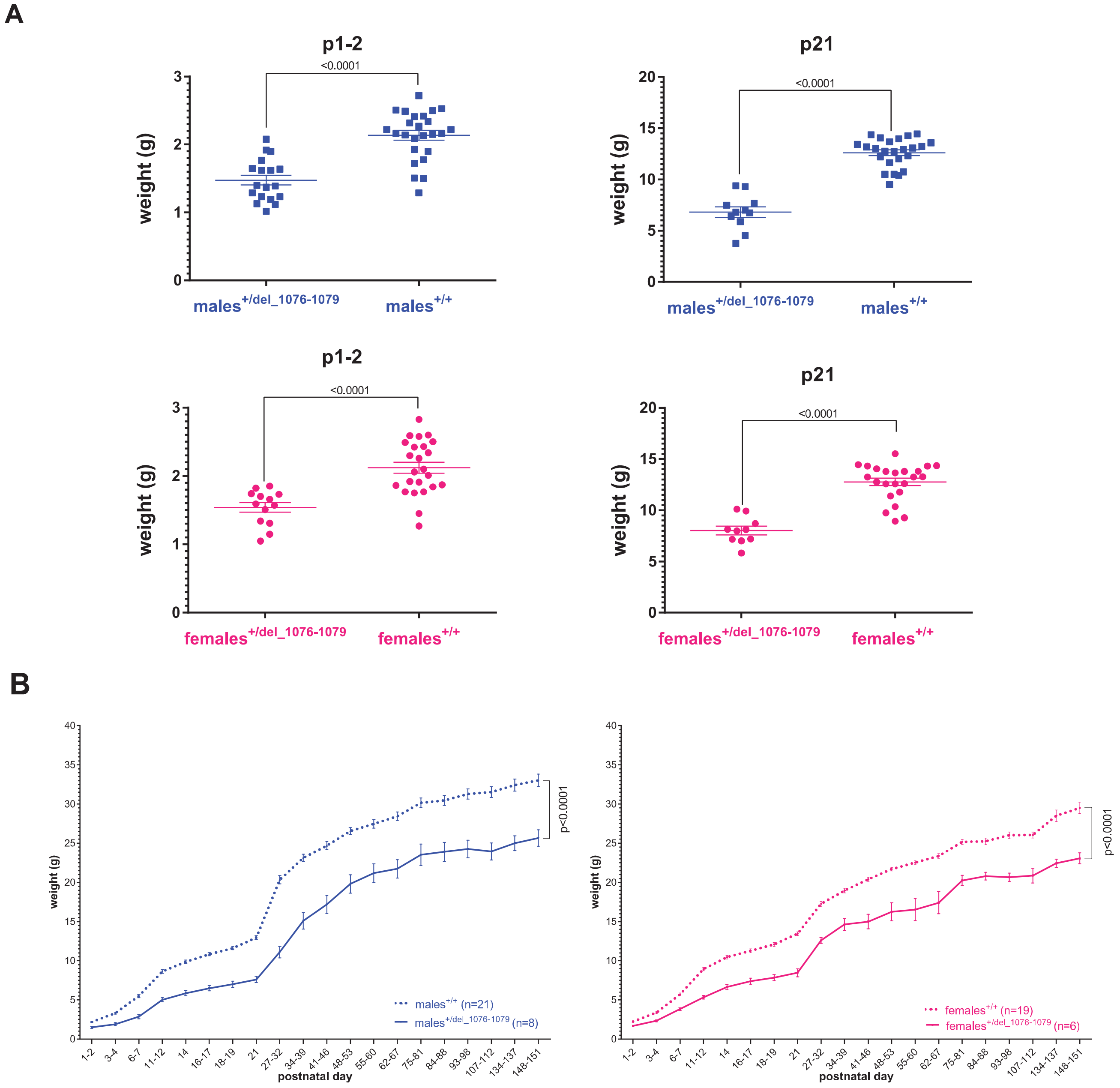

Supplementary Figure 16: A. p1-2 and p21 weight data by sex of the mice. *Ehmt2*$^{+/del\_1076-1079}$ mice have significantly lower body weight regardless of sex at both time points. Statistical significance was calculated using two-tailed Student's t-test.
B. Weight curves of mice surviving the entire 5 month (p151) duration of the study. Compare to weight curve data provided in Figure 6A and 6B. Statistical significance of the differences between the genotypes for each sex of the animals was calculated using two-way repeated measures ANOVA. Average values ±SEM are shown in A and B.

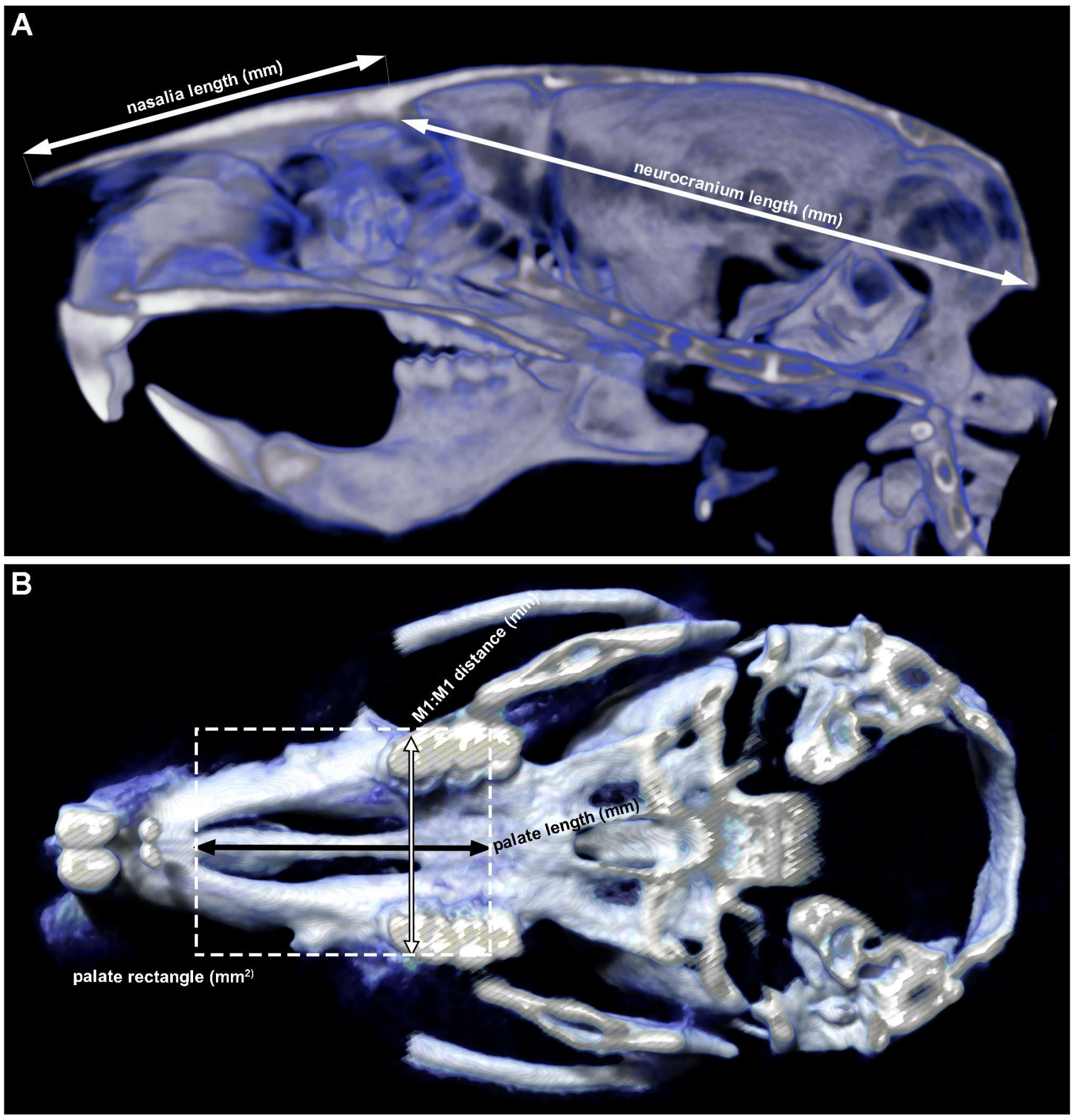

Supplementary Figure 17: Craniometric analyses: A. Sagittal skull section. White arrows show the strategy of nasalia and neurocranium length measurements. B. 3D palate reconstruction. Palate length was assessed from the distal rim of the palate arch (fissure) to the suture between maxilla and palate bone. Palate width corresponds to molar 1: molar 1 (M1:M1) distance. Palate rectangle (dashed) was calculated by multiplying the latter two values. See Figure 6D for further details.

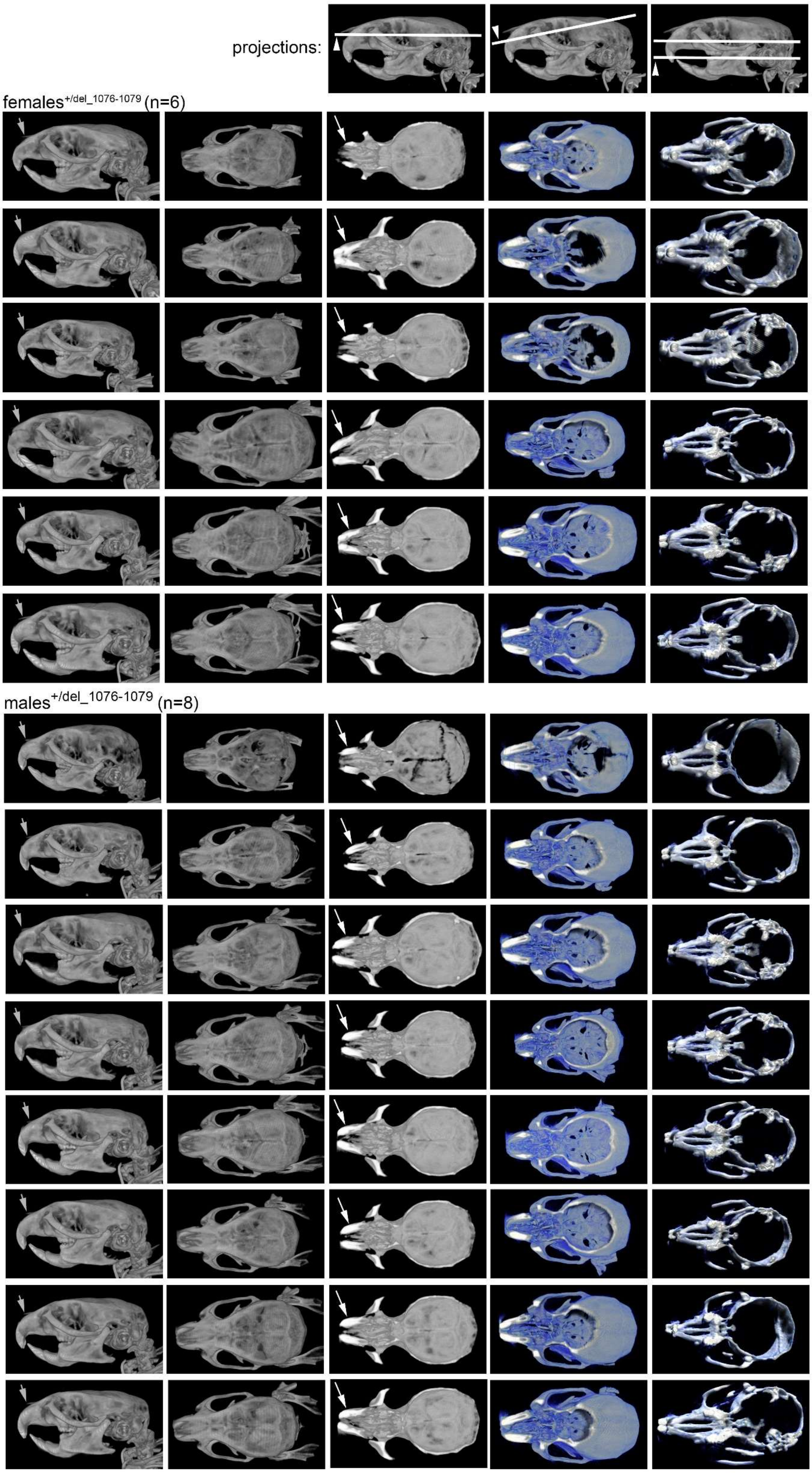

Supplementary Figure 18: Overview of microCT data from all tested *Ehmt2*$^{+/del\_1076-1079}$ animals. Left to right. Lateral view at the 3D skull reconstruction. Top view at the 3D skull reconstruction. For the remaining three columns of the projections - white lines show the section plain and arrowheads highlight the view direction (summarized in three images above the layout). Grey arrows in the first column highlight short nasalia, white arrows in the third column highlight the variability of the "bent" nose phenotype in the tested cohort. Compare to Figure 6C and 6D and Supplementary Figure 19.

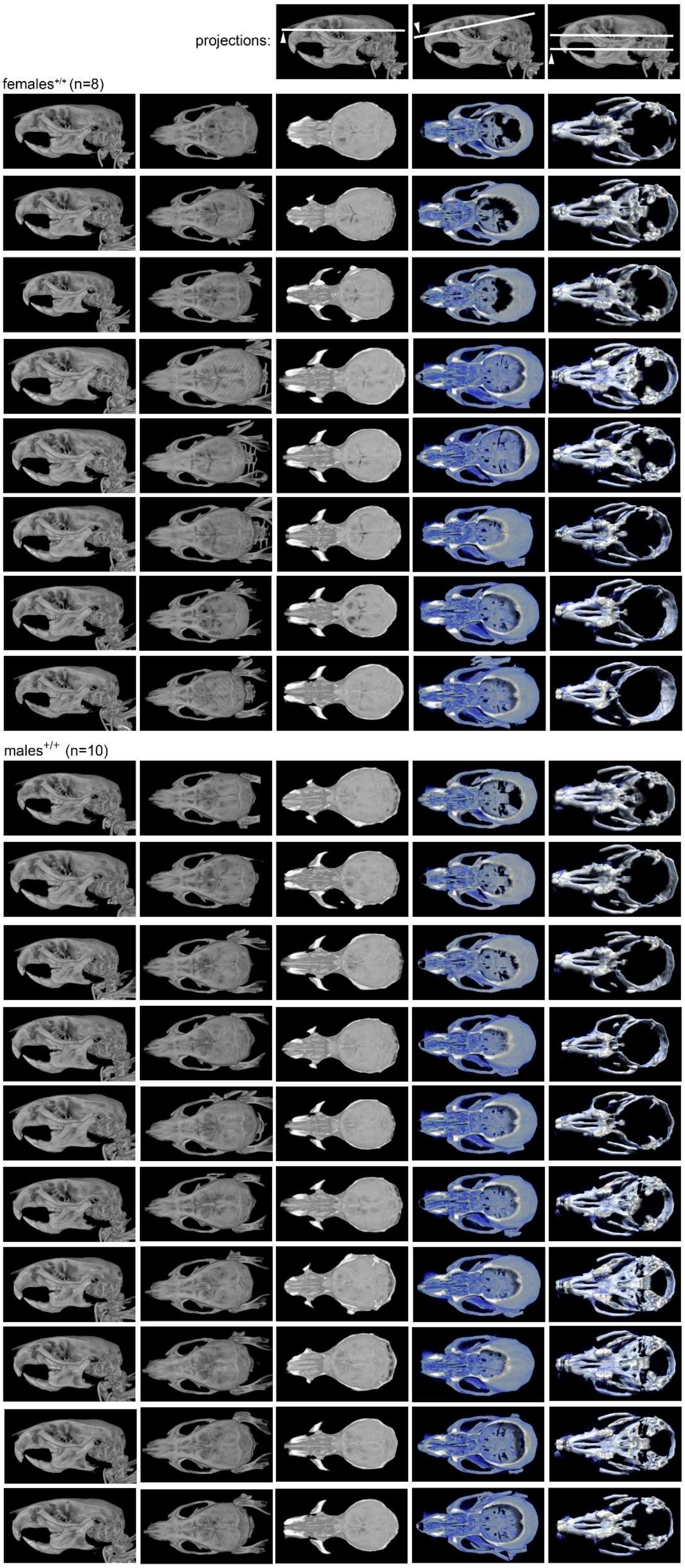

Supplementary Figure 19: Overview of microCT data from all tested *Ehmt2*$^{+/+}$ animals. Left to right. Lateral view at the 3D skull reconstruction. Top view at the 3D skull reconstruction. For the remaining three columns of the projections - white lines show the section plain and arrowheads highlight the view direction (summarized in three images above the layout). Compare to Figure 6C and 6D and Supplementary Figure 18.

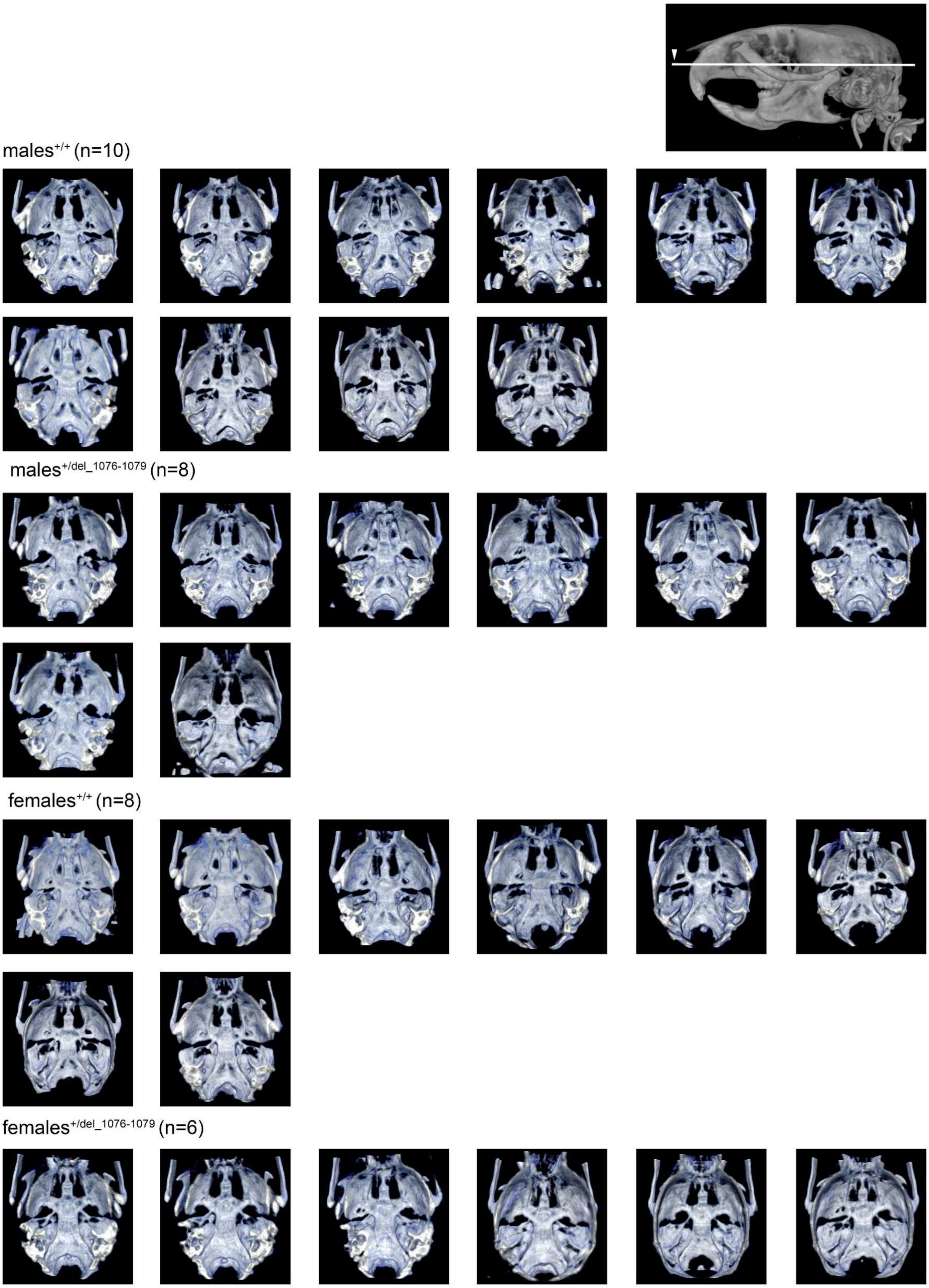

Supplementary Figure 20:3D reconstruction of the skull base viewed from the top. Section plane and view direction is shown in the image above the layout. There do not seem to be any abnormalities in sphenoid bone formation in *Ehmt2*$^{+/del\_1076-1079}$ mice in comparison to *Ehmt2*$^{+/+}$ animals.

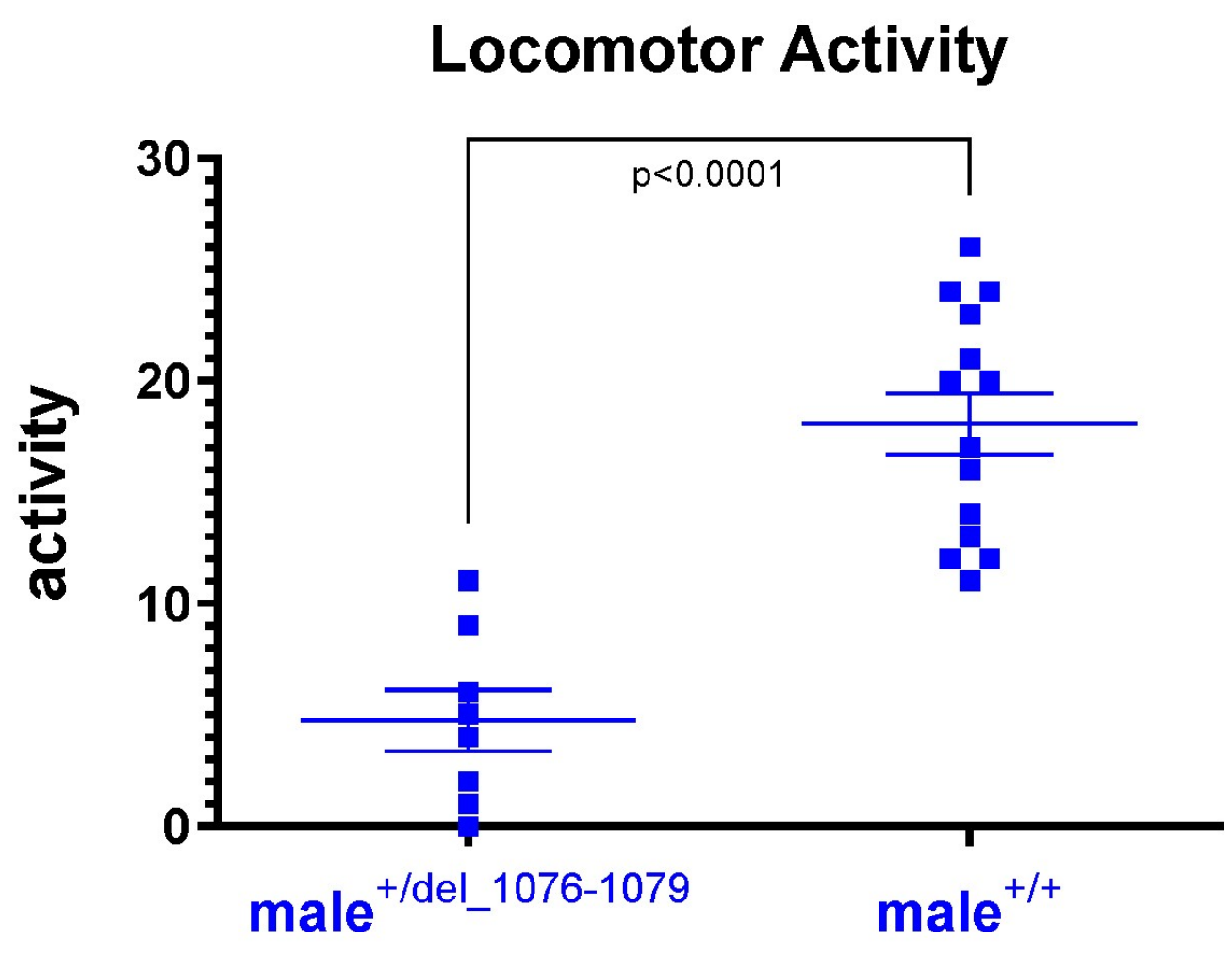

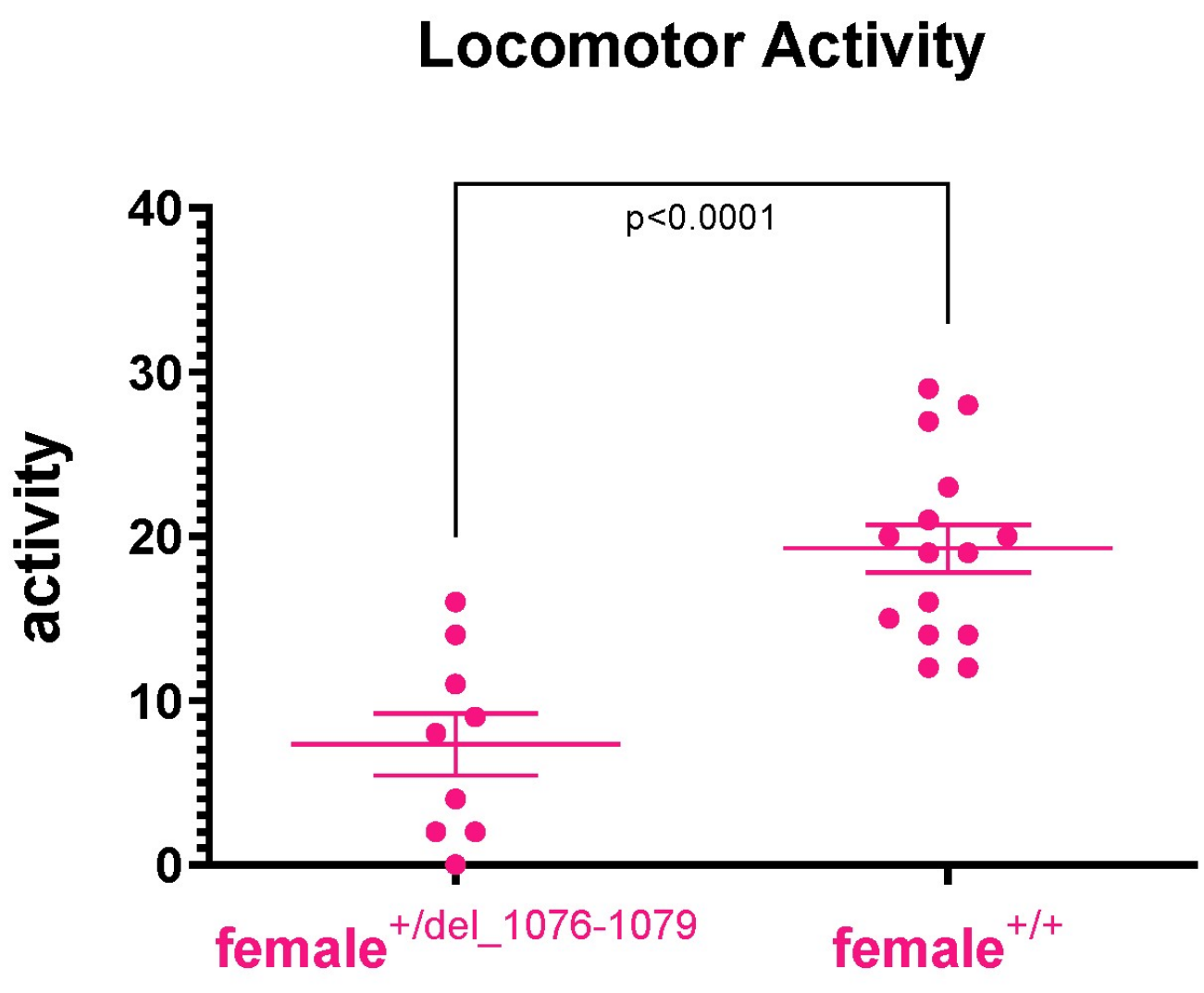

Supplementary Figure 21:Differences in the locomotor activity (scored as squares crossed) which is significantly lower in *Ehmt2*$^{+/del\_1076-1079}$ mice. Statistical significance was calculated using two-tailed Student's t-test. Average values ±SEM are shown.

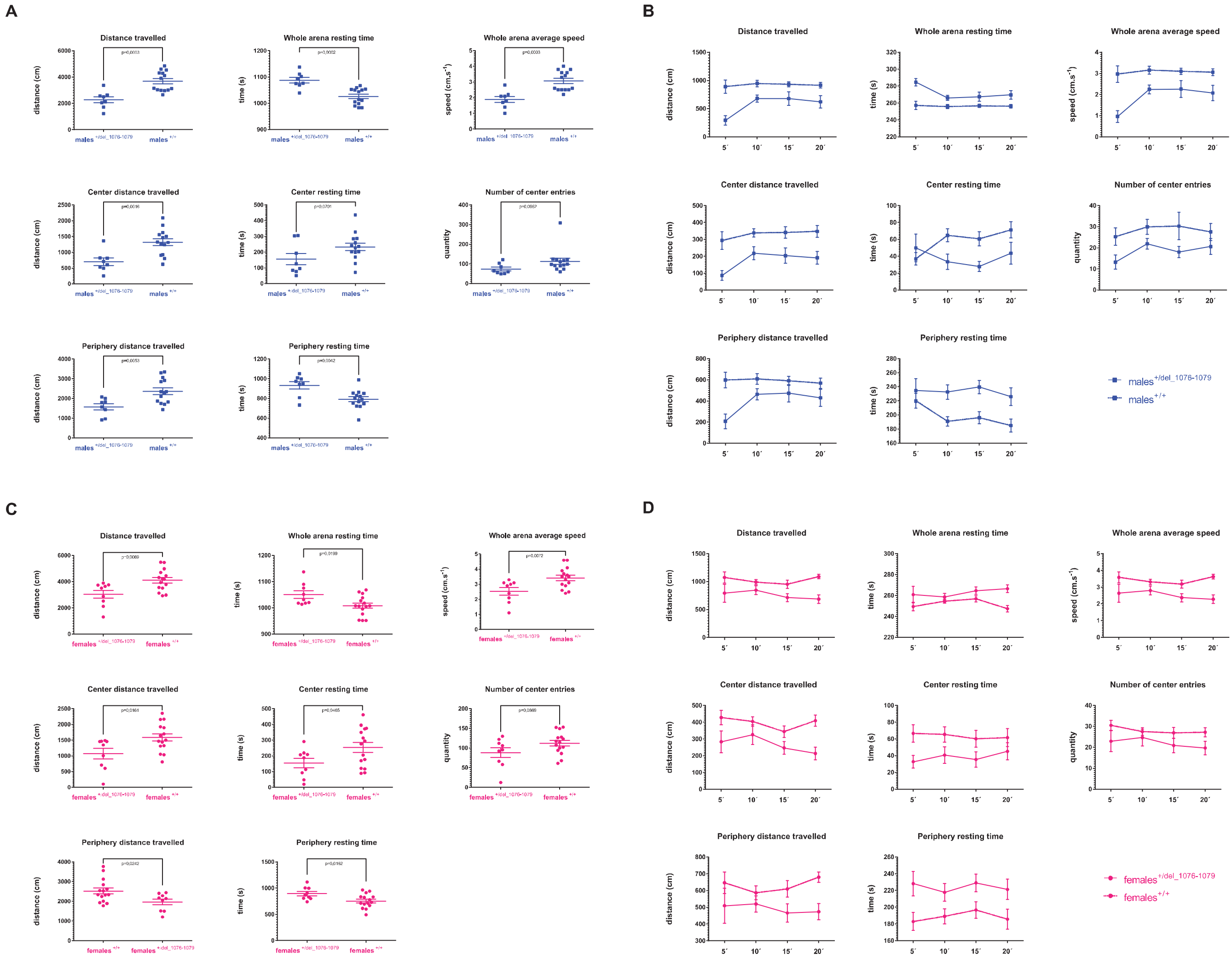

Supplementary Figure 22: Summary data (20 minutes) for individual open-field measures are shown for males (A) and females (C). Corresponding interval data (4x5 minutes) are outlined for both sexes (B and D). Statistical significance was calculated using two-tailed Student's t-test in A and C. Average values ±SEM are shown in A-D.

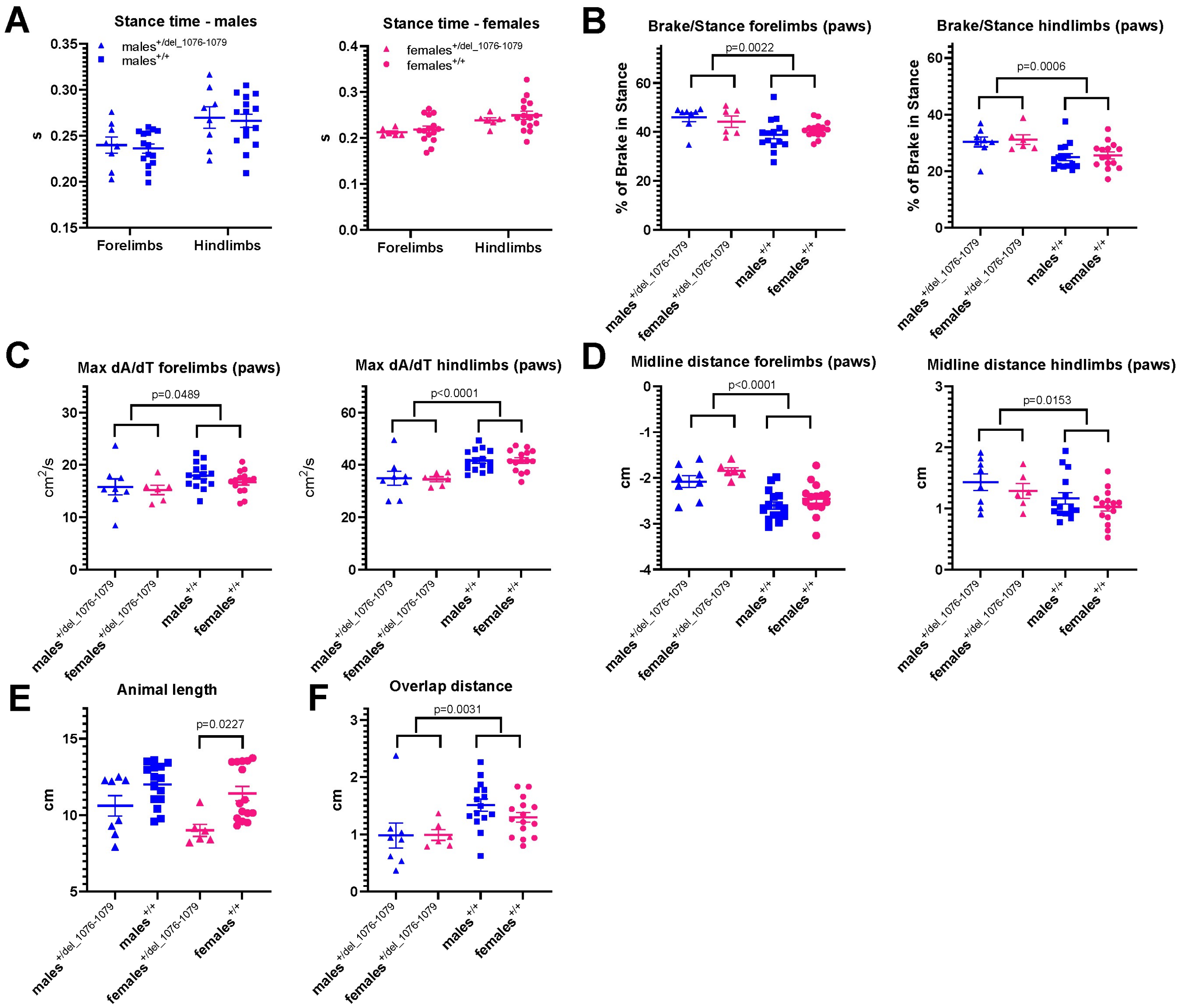

Supplementary Figure 23:*Ehmt2*$^{+/del\_1076-1079}$ mice exhibit significant alterations in gait parameters compared to *Ehmt2*$^{+/+}$ mice.

A. Stance duration (the time during which the paw remains in contact with the treadmill belt) did not differ between the two groups. B. However, the braking phase - „landing“ of the paw to the ground—was significantly prolonged in both the forelimbs and hindlimbs of *Ehmt2*$^{+/del\_1076-1079}$ mice indicating reduced stability and a need for more precise control of load distribution during stance initiation. C. Difficulties with deceleration and limb loading in *Ehmt2*$^{+/del\_1076-1079}$ mice were supported by a reduced Maximal rate of change (Max dA/dT) in paw contact area with the treadmill belt during the braking phase, particularly in the hindlimbs. D. Regarding the posture-related parameters, we observed that the midline distance (the mediolateral distance from the body's transverse midline to the paws) was significantly reduced in *Ehmt2*$^{+/del\_1076-1079}$ mice for the forelimbs (negative midline distance on the *y*-axis is due to the directionality of the measurement) but increased for the hindlimbs. Because midline distance correlates positively with body length, the reduced body length observed in *Ehmt2*$^{+/del\_1076-1079}$ mice E. may partly explain the decreased forelimb midline distance, particularly in females, but partly could be a result of reduced strength to extend the forelimbs to the extent that the *Ehmt2*$^{+/+}$ mice are capable of. However, this morphological difference cannot account for the increased hindlimb midline distance in the mutant mice, which looks like they stretch their strides too far back or carry the paw further back than normal indicating worsened coordination of gait phases and reduced stability during locomotion. F. Finally, *Ehmt2*$^{+/del\_1076-1079}$ mice exhibited decreased overlap distance, meaning that hind paws failed to reach the forepaw placement, resulting in a larger gap and indicating impaired inter-limb coordination.
Statistical significance was calculated using two-tailed Student's t-test. Average values ±SEM are shown.

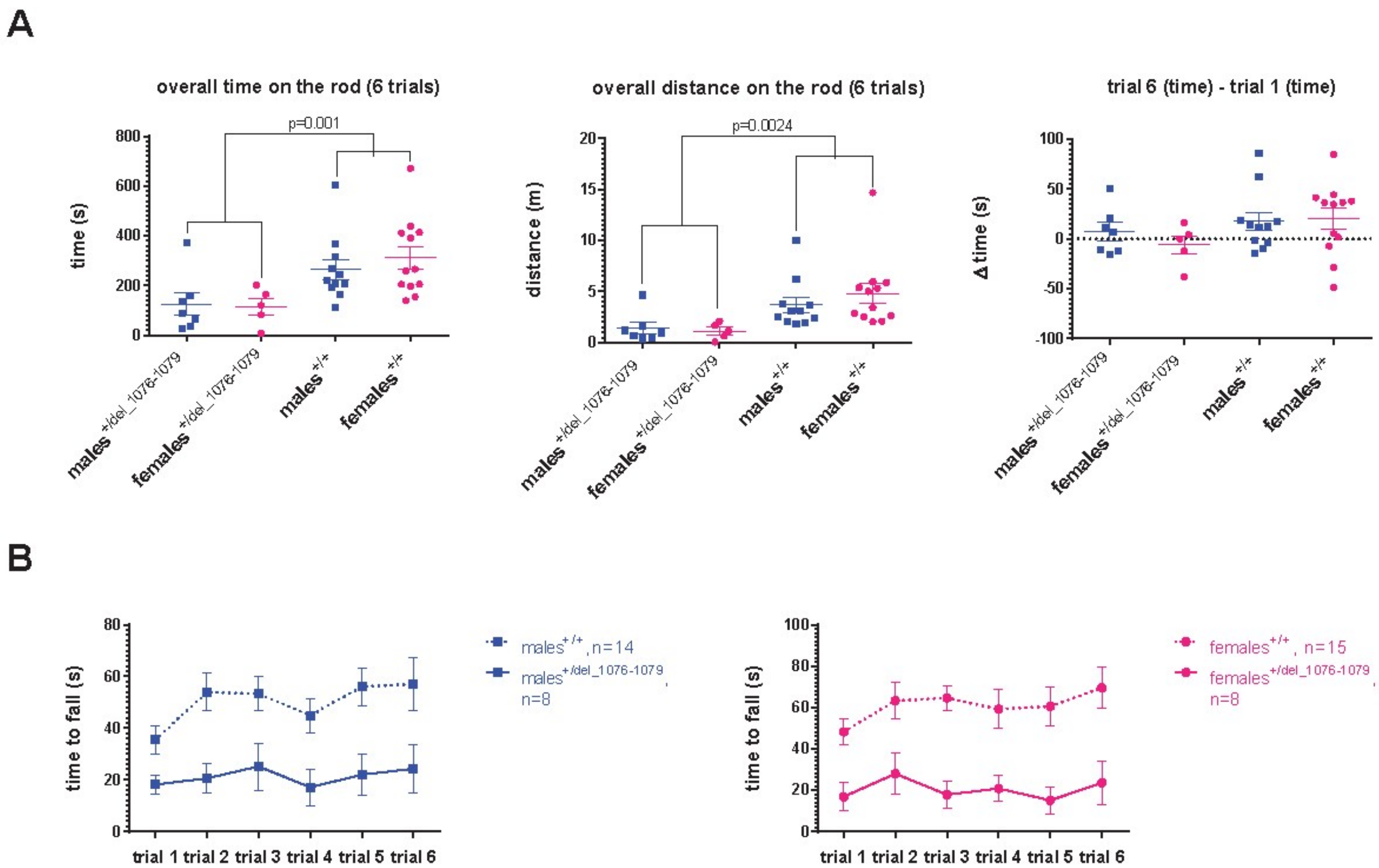

Supplementary Figure 24: A. Summary data (6 trials) for time on the rod and distance travelled on the rod are shown. The difference in time-to-fall in trial 6 and trial 1 (Δ time) is not significantly different between $\textit{Ehmt2}^{+/del\_1076\text{-}1079}$ and $\textit{Ehmt2}^{+/+}$ mice. B. Individual interval data for times-to-fall are shown for comparison to overall data.

Statistical significance was calculated using two-tailed Student's t-test in A. Average values $\pm$SEM are shown in A and B.

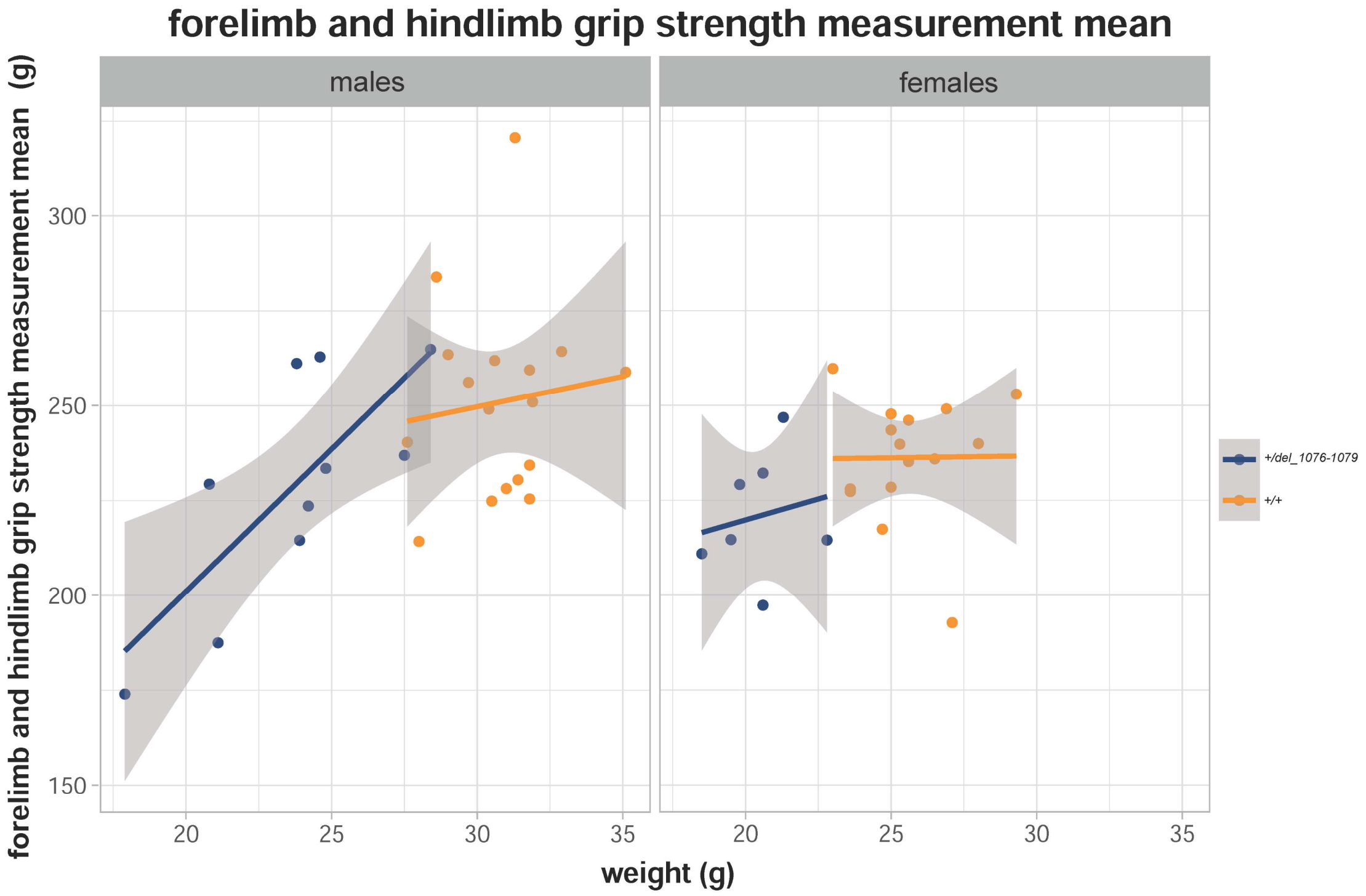

Supplementary Figure 25: Grip strength data were analyzed as follows. Triplicate measurements for each mouse were averaged, and a linear mixed-effects model was applied to assess group differences. Genotype, sex, and their interaction were included as fixed effects, with body weight as a covariate. The date of measurement was included as a random effect to account for variability between sessions. To evaluate group differences, contrasts were specified to compare mutant (females and males combined) versus wild-type animals (also combined), as well as within each sex separately. Additionally, the data were visualized with body weight on the x-axis and grip strength on the y-axis, plotted separately for each sex to match the structure of the statistical model.

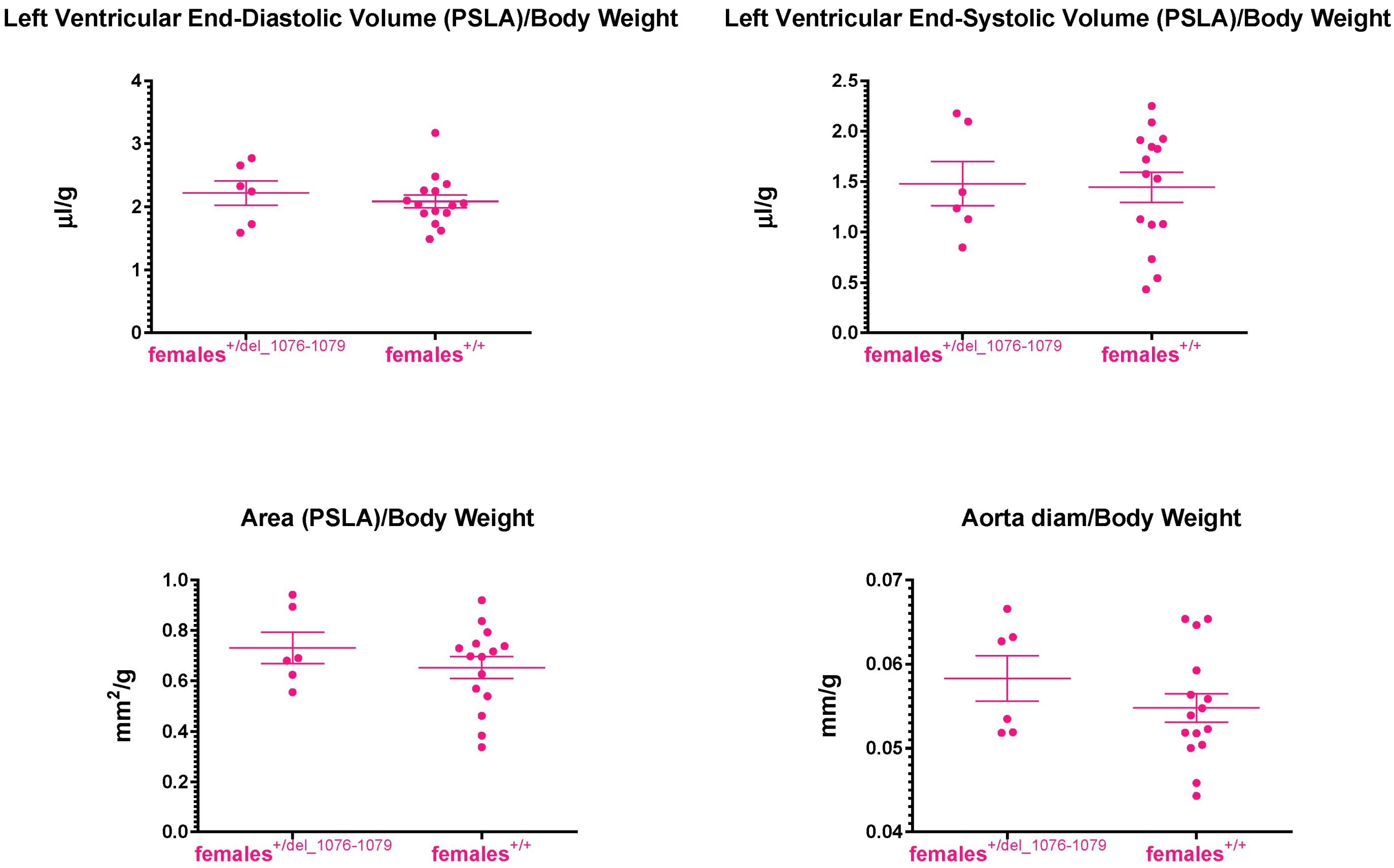

Supplementary Figure 26: Left ventricular morphology in female Ehmt2$^{+/del_1076\text{-}1079}$ mice assessed by echocardiography.

Transthoracic echocardiography revealed that female Ehmt2$^{+/del_1076\text{-}1079}$ mice did not exhibit any significant alterations in left ventricular morphology compared to Ehmt2$^{+/+}$ controls. Left ventricular end-diastolic and end-systolic volumes normalized to body weight were comparable between groups, with no significant differences detected. Similarly, no differences were observed in the parasternal long-axis (PSLA) area normalized to body weight or in the aortic diameter normalized to body weight. Statistical analysis was performed using a two-tailed Student's t-test; none of the comparisons reached statistical significance (significance threshold: $p < 0.05$).

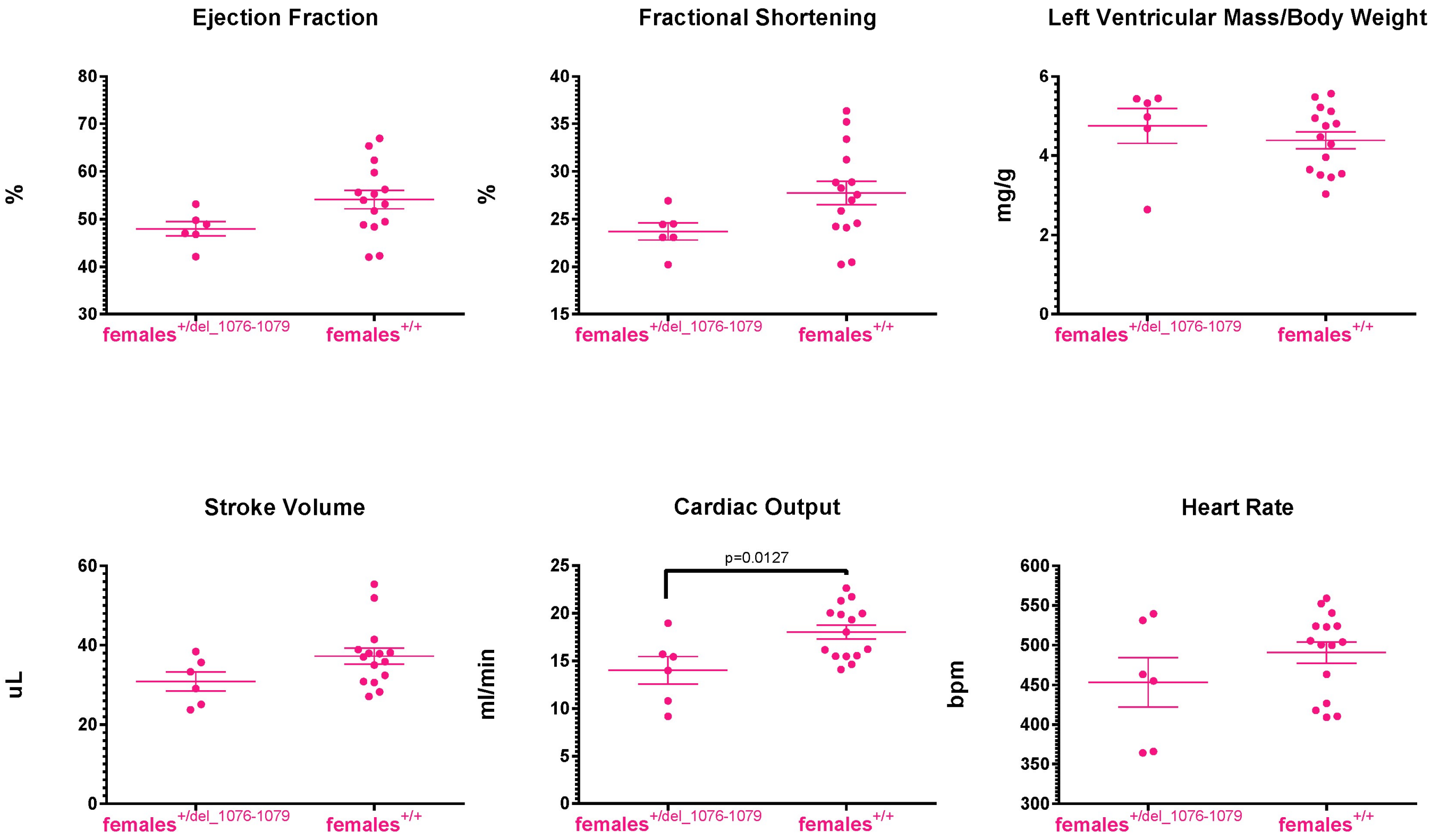

Supplementary Figure 27: Cardiac functional parameters in female Ehmt2$^{+/del_1076-1079}$ mice assessed by echocardiography.

Transthoracic echocardiography showed that female Ehmt2$^{+/del_1076-1079}$ mice did not show significant impairment in functional cardiac parameters compared to Ehmt2$^{+/+}$ controls. Although a mild downward trend in ejection fraction and fractional shortening was observed in mutant animals, these differences did not reach statistical significance. Left ventricular mass normalized to body weight and stroke volume were also comparable between groups. Cardiac output was significantly reduced in Ehmt2$^{+/del_1076-1079}$ mice; however, this decrease was attributable to a lower heart rate rather than impaired ventricular performance. Overall, echocardiographic findings indicate preserved cardiac structure and function in female Ehmt2$^{+/del_1076-1079}$ mice under basal conditions. Statistical analysis was performed using a two-tailed Student's t-test. Statistical significance was defined as $p < 0.05$.

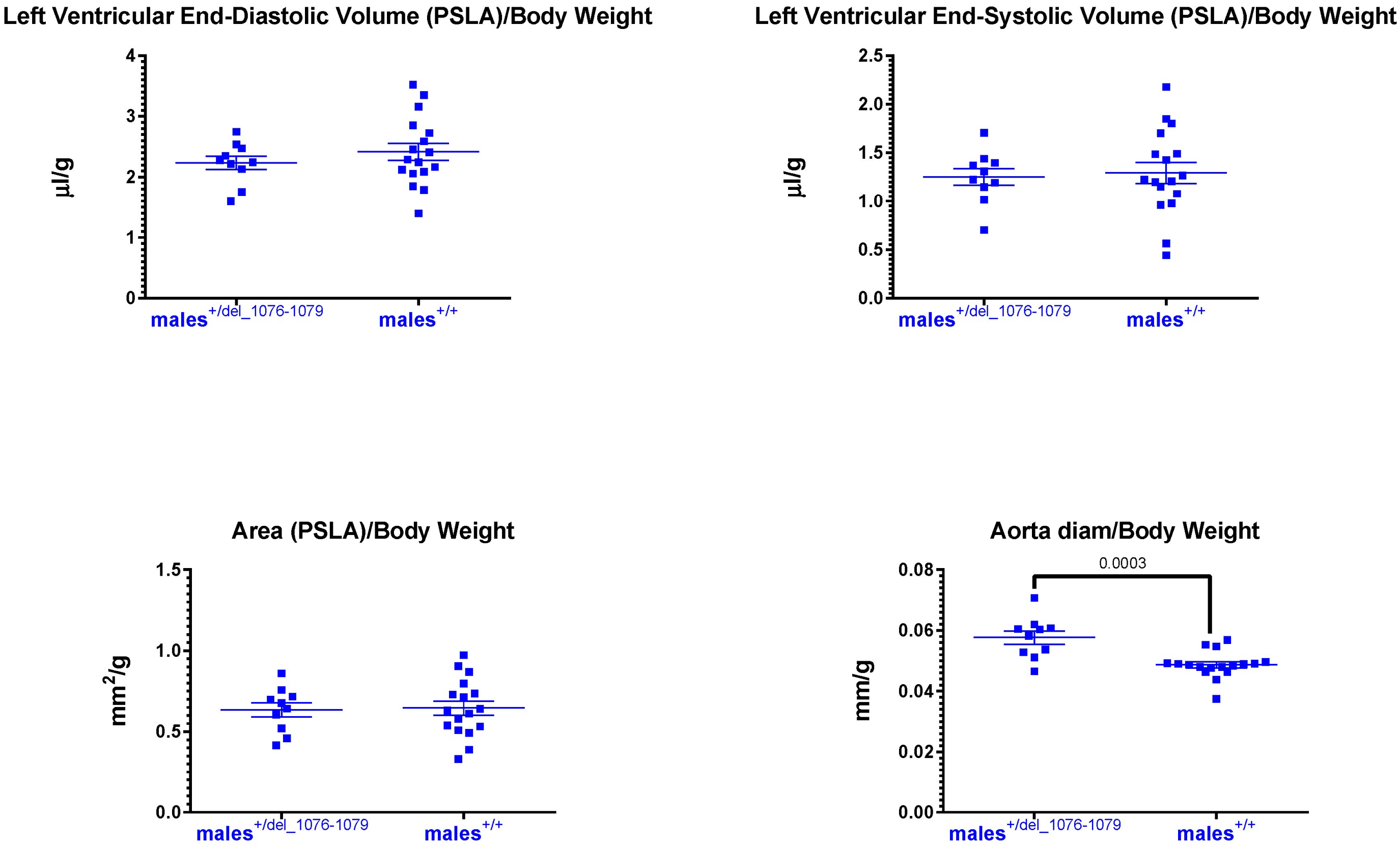

Supplementary Figure 28: Left ventricular morphology in male Ehmt2$^{+/del_1076\text{-}1079}$ mice assessed by echocardiography.

Transthoracic echocardiography revealed that male Ehmt2$^{+/del_1076\text{-}1079}$ mice did not exhibit significant alterations in left ventricular morphology compared with Ehmt2$^{+/+}$ controls. Left ventricular end-diastolic and end-systolic volumes normalized to body weight were comparable between groups, with no significant differences detected. Similarly, no differences were observed in the parasternal long-axis (PSLA) area normalized to body weight. In contrast, the aortic diameter normalized to body weight was significantly increased in Ehmt2$^{+/del_1076\text{-}1079}$ males. This alteration was not accompanied by changes in left ventricular morphology. Statistical analysis was performed using a two-tailed Student’s t-test. Statistical significance was defined as $p < 0.05$.

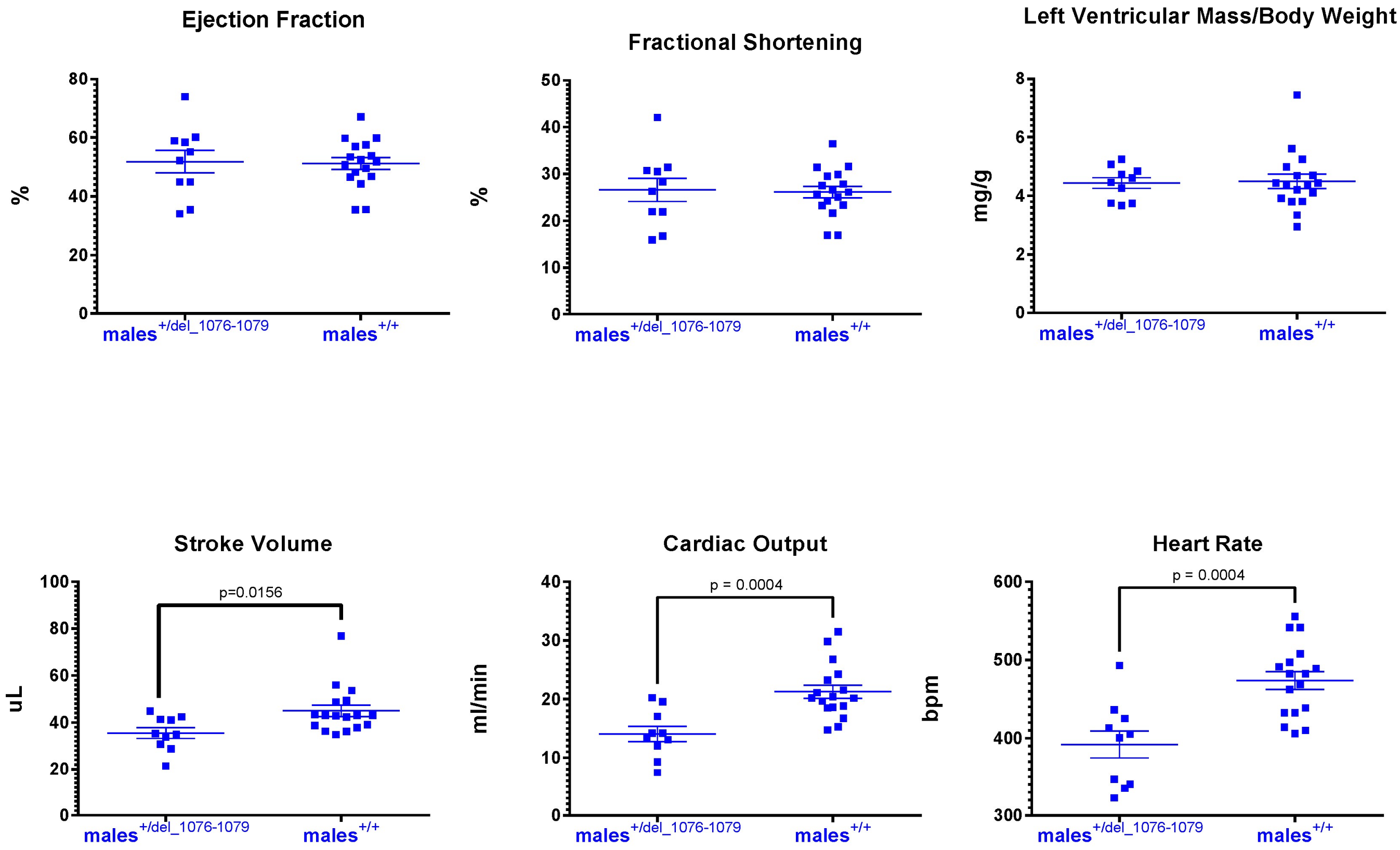

Supplementary Figure 29: Cardiac functional parameters in male Ehmt2$^{+/del_1076\text{-}1079}$ mice assessed by echocardiography.

Transthoracic echocardiography showed that male Ehmt2$^{+/del_1076\text{-}1079}$ mice exhibited no significant differences in ejection fraction or fractional shortening compared with Ehmt2$^{+/+}$ controls. Left ventricular mass normalized to body weight was also unchanged. In contrast, stroke volume was significantly reduced in Ehmt2$^{+/del_1076\text{-}1079}$ mice, accompanied by a significant reduction in cardiac output and heart rate. The reduction in stroke volume occurred in the absence of changes in ejection fraction or left ventricular mass, indicating preserved myocardial contractility. The decrease in cardiac output was primarily driven by the lower heart rate. Statistical analysis was performed using a two-tailed Student’s t-test. Statistical significance was defined as $p < 0.05$.

A Database of Genomic Variants

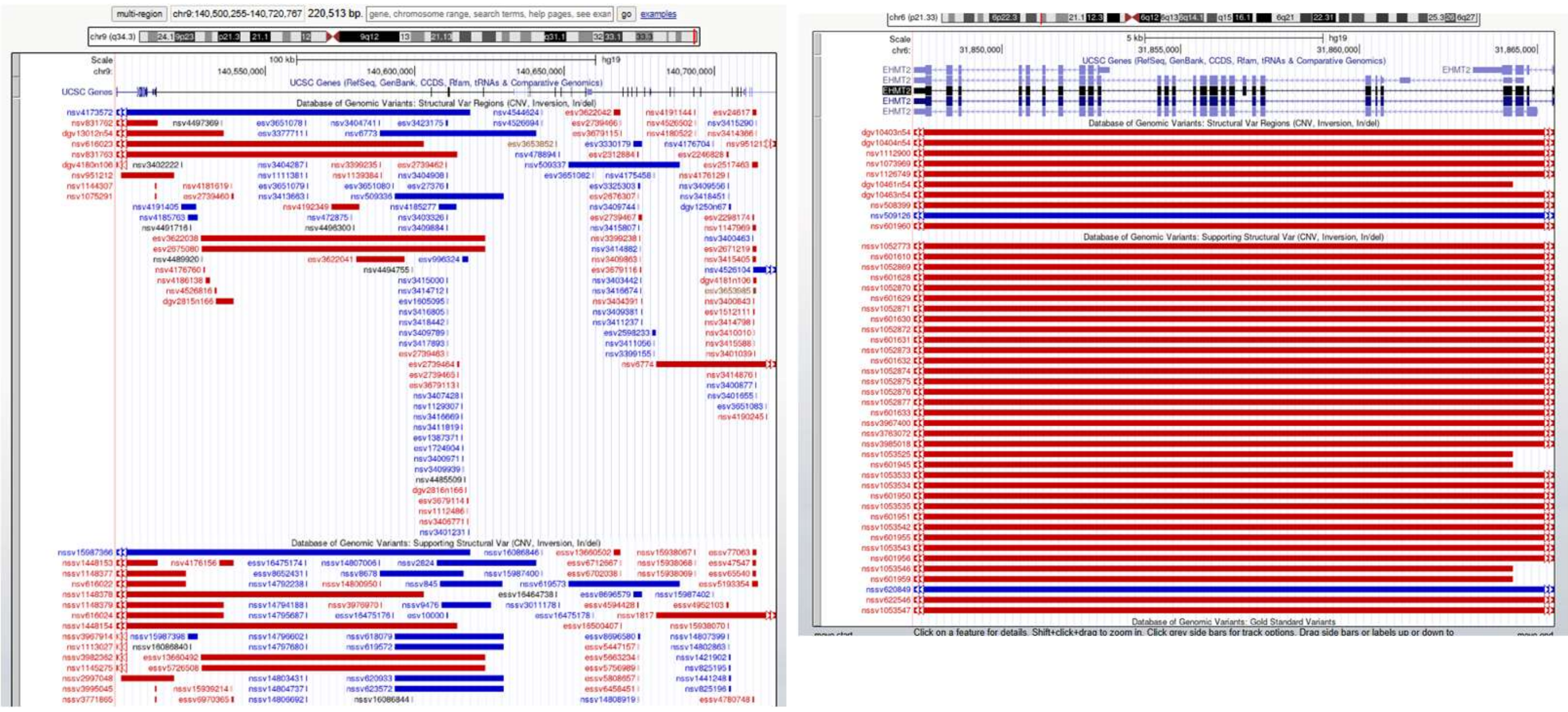

EHMT1 **EHMT2**

B

DECIPHER

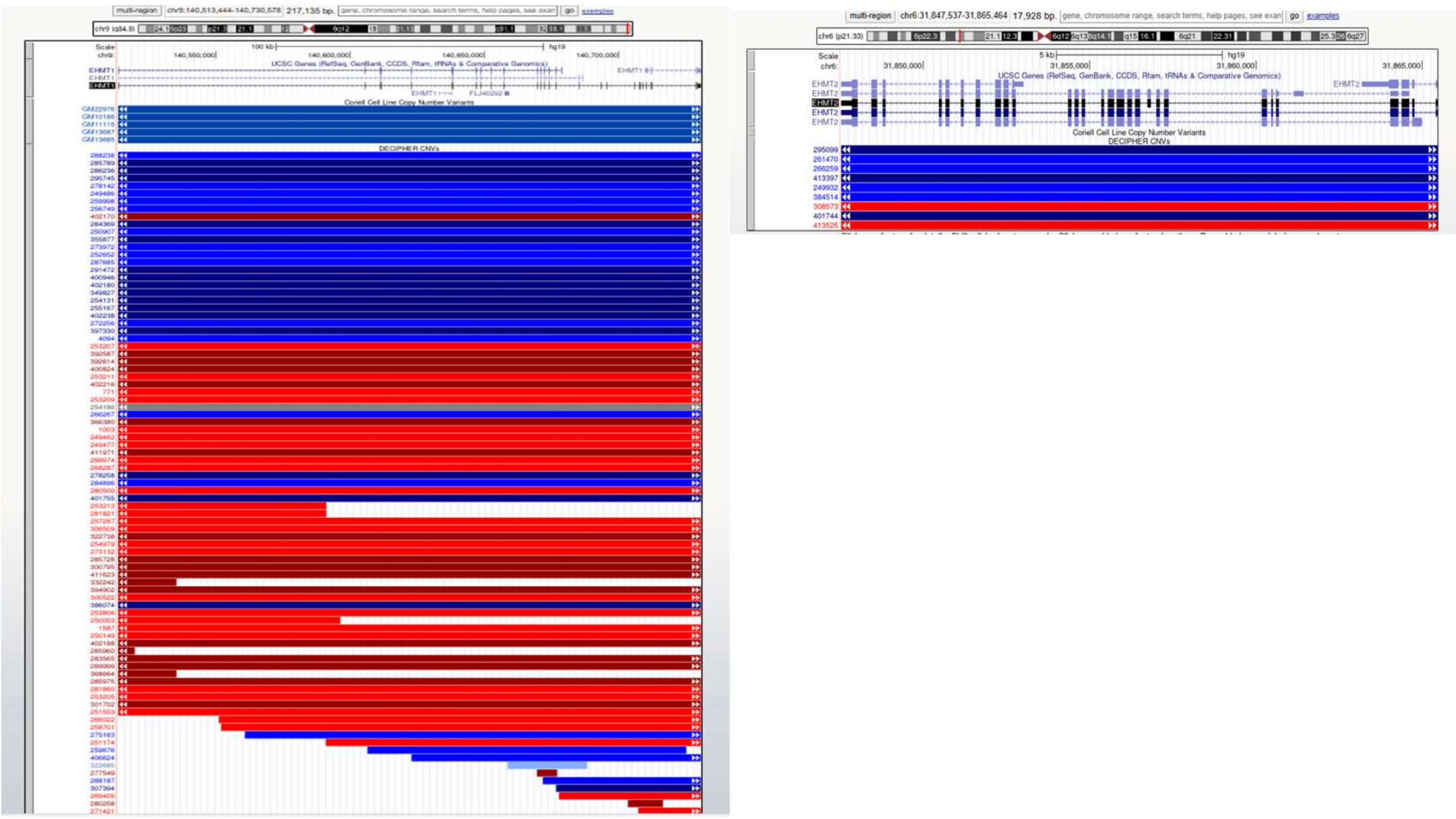

Supplementary Figure 30. A. Database of Genomic Variants (DGV) shows sporadic gene deletions in *EHMT1* and frequent deletion events in *EHMT2.*
B. DECIPHER database shows genic deletions in *EHMT1* but not in *EHMT2.*

Supplementary Table 1. Structural interpretation of the identified G9a variants

Variant	Positioning	Structural effect	Functional impact
Del 1076-9	histone 3 binding site	loss of polar interactions with histone 3 tail	affected binding to histone 3
A1077S	histone 3 binding site	sterical clashes with R8 of histone 3	affected binding to histone 3
A1077T	histone 3 binding site	sterical clashes with R8 of histone 3 and proximal residues (D1074)	affected binding to histone 3
N1112S	SAM binding site	loss of the interaction with an amino group of SAM	affected binding to SAM
F1158L	contact area between SAM and histone 3 sites	creation of an empty space in the catalytic site	disturbed enzymatic catalysis
K1162R	contact area between SAM and histone 3 sites	sterical clashes and/or loss of polar interactions in the catalytic pocket	affected binding to histone 3 and SAM
Del 1163-4	contact area between SAM and histone 3 sites	loss of interactions with residues of the histone 3 binding site	affected binding to histone 3

Supplementary Table 2. Thermostability of the GLP variants determined by DSF. Values represent mean with standard deviation.

Protein	T_{m1} (°C)	T_{m2} (°C)
WT	41.2 ± 0.2	51.7 ± 0.3
C1073Y	39.5 ± 0.3	N.A.[1]
R1197W	N.A.[2]	N.A.[2]

[1] not applicable. The protein exhibited one melting point, only.

[2] not applicable. The protein was insoluble and could not been purified.

Supplementary Table 3. Kinetic parameters of G9a (A1077S) and GLP (C1073Y) variants with detectable residual activity. Data were fitted according to Michaelis-Menten equation.

**Protein**	**Substrate**	**V_{max} (nmol.min^{-1}mg^{-1})**	**K_M (µM)**	**k_{cat} (s^{-1})**	**k_{cat}/K_M (s^{-1}/µM)**
G9a Wild-Type	histone 3	394-+ 20	1.99 +-0.43	0.213 -+ 0.011	0.106 +- 0.023
G9a A1077S	histone 3	183 +-31	19.8 +- 8.1	0.099 -+0.016	0.005 -+ 0.002
G9a Wild-Type	SAM	618 +-64	17.0+-4.7	0.334 -+ 0.035	0.0194 -+ 0.0057
G9a A1077S	SAM	138 +-11	4 -+ 1.1	0.075 -+ 0.006	0.0188 +- 0.0054
GLP Wild-Type	histone 3	309 -+ 11.1	2.88 -+ 0.43	0.169 -+ 0.006	0.059 -+ 0.009
GLP C1073Y	histone 3	457 -+ 41	15.9 -+ 3.2	0.215 -+ 0.022	0.015 -+ 0.003
GLP Wild-Type	SAM	261 -+ 23	4.0 -+ 1.2	0.143 -+ 0.013	0.035 -+ 0.011
GLP C1073Y	SAM	223 -+ 19	6.1 -+ 1.6	0.122 -+ 0.010	0.020 -+0.006

Supplementary Table 4. Number of animals for each time-point/genotype/sex in weight curves.

Time points (days)	females +/del_11076_1079	females +/+	males +/del_11076_1079	males +/+
1-2	24	13	25	18
3-4	24	11	25	15
6-7	24	10	25	15
11-12	23	10	25	13
14	23	10	24	11
16-17	23	10	24	11
18-19	23	10	24	11
21	23	10	21	11
24-25	19	10	24	9
27-32	23	9	24	11
34-39	23	8	24	11
41-46	23	8	24	10
48-53	23	8	24	10
55-60	23	7	24	10
62-67	23	7	24	10
69-74	23	7	24	10
75-81	23	7	24	10
84-88	23	7	24	10
93-98	23	7	24	10
107-112	23	6	24	10
134-137	19	6	21	8
148-157	19	6	21	8

Supplementary Table 5. Primers used for site-directed mutagenesis of expression plasmids producing recombinant proteins

**primers for EHMT2 variants:**
T961I
for: ATCAGCGAAAATTGTGAAACCAGCATTATGAATATTGACCGCAATATTACC
rev: GGTAATATTGCGGTCAATATTCATAATGCTGGTTTCACAATTTTCGCTGAT
Del1076-9
for: CGTGAAGATGATAGCTACCTGTTTGA
rev : GGCATCGGAAATCAGTTCACCA
A1077S:
for: GCTATCATCTTCACGAACATCGCTTTCGGCATCGGAAATCAGTTC
rev: GAACTGATTTCCGATGCCGAAAGCGATGTTCGTGAAGATGATAGC
A1077T:
for: TCTTCACGAACATCGGTTTCGGCATCGGAAATCA
rev: TGATTTCCGATGCCGAAACCGATGTTCGTGAAGA
N1112S
for: GGATCACACAGATGACTAATAAAGCGGCTGATGTTACCATAATAA
rev: TTATTATGGTAACATCAGCCGCTTTATTAGTCATCTGTGTGATCC
F1158L
for: AGAACTGGGTTTTGATTATGGTGATCGTCTGTGGGATATCAAAAGC
rev: GCTTTTGATATCCCACAGACGATCACCATAATCAAAACCCAGTTCT
K1162R
for: GCACTGACAGGTAAAATACTTGCTGCGGATATCCCAGAAACGATCACCATA
rev: TATGGTGATCGTTTCTGGGATATCCGCAGCAAGTATTTTACCTGTCAGTGC
Del 1163-4
for: TATTTTACCTGTCAGTGCGGTAGCGAAAAAT
rev: TTTGATATCCCAGAAACGATCACCATAATCAAAAC
**primers for EHMT1 variants:**
C1073Y
for: GATTGTAGCAGCAGCAATTATATGTGTGGTCAGCTGAG
rev: CTCAGCTGACCACACATATAATTGCTGCTGCTACAATC
R1197W
for: CCCGTTTTTATGGTAATGTGAGCTGGTTTATTAACCATCATTGTGAACC
rev: GGTTCACAATGATGGTTAATAAACCAGCTCACATTACCATAAAAACGGG

Supplementary Notes

1. Clinical description of the patients with *EHMT2 de novo* variants.

Proband 1 (P1) is a girl born at term from the second gravidity, with positive 2nd trimester screening but normal amniocentesis. Birth parameters were normal. The family history is unremarkable. At the age of six months, she underwent surgery for pylorostenosis and was found to be intolerant to cow milk. Stenosis of arteria pulmonalis was found by cardiologic examination. Central hypotonic syndrome was apparent from the age of 6 months, neurological examination ruled out a neuromuscular disorder. The girl shows facial dysmorphism – flat face, hypertelorism, coarse features, high forehead, thin lips, short neck, dysplastic helices; and has unusually big toe. Currently, she suffers from severe developmental delay, cognitive impairment and signs of autism. Emotion-induced stereotypic movements of hands, bruxism and self-mutilation in anger are present in her behavior. EEG showed unspecific bilateral temporal-occipital-parietal focal activity. Central atrophy and atrophy of frontal lobes, enlarged megacisterna magna with arachnoid cyst in posterior fossa, corpus callosum thinning and widening of perioptic CSF spaces were found on MRI. She understands the instructions, but she is unable to speak and is fully dependent on another person care. She is able to walk independently only short distances, while using wheelchair for longer ones. Fine motor skills are suboptimal. She attends a school for children with special needs. Growth and sexual maturation are delayed, with all the growth parameters (height, weight, head circumference) being bellow the third percentile.

Proband 2 (P2) was remitted to the undiagnosed rare diseases program SpainUDP[1] after testing negative for candidate gene panels. The proband suffered from global developmental delay characterized by slow development of gross motor milestones and expressive language delay, hypotonia without weakness, coarctation of aorta, nephrocalcinosis and renal medullar cystic disease. The proband had brachycephaly, plagiocephaly with very flat occiput, broad face and midface hypoplasia, synophrys, sparse medial eyebrows, epicanthus, lateral deviation of upslanted palpebral fissures, mildly everted lower eyelids, palpebral ptosis, anteverted nares, smooth philtrum and carp-like mouth. In addition, the proband had dental diastema, microdontia, persistent fetal fingertip pads and scoliosis. From the clinical point of view, the patient's phenotype overlaps significantly with the clinical characteristics of Kleefstra syndrome. The proband is a male born in 2014 to a 35-year-old mother after a pregnancy complicated by polyhydramnios and intrauterine growth restriction detected at 34 weeks of gestation. The mother was infected by parvovirus B19 during the third trimester of pregnancy, but the newborn's serology was negative for parvovirus (and also for cytomegalovirus). Delivery was induced at 37 weeks of gestation and caesarean section was urgently carried out since a double entanglement and a real knot in the cord were observed. Birth measurements were 2.440 kg of weight (p‹3), 50.5 cm of height (p10-50), a cephalic perimeter of 34.5 cm (p10-50) and an Apgar score of 5/8. The neonate was hospitalized for 15 days after childbirth due to fetal distress assessed during the pregnancy, and clinical evaluation indicated he had jaundice, moderate neonatal depression, membranous septal defect, patent foramen ovale, bilateral cryptorchidism, micropenis, connatal anemia and neonatal hypotonia. At the age of 20 days, he was diagnosed with coarctation of the aorta after an episode of cyanosis and apneas, which required hospitalization and surgical aortic resection and anastomosis. During this hospitalization period, a first electroencephalogram (EEG) showed a pathological profile, although no seizures were observed. However, a second EEG recording revealed no epileptic activity. Neurological examination showed that the baby had dysmorphic facial features and axial hypotonia and therefore he was referred to the neuropediatrician for a follow-up. Three months after birth, he was hospitalized again due to a second episode of cyanosis and apneas, which resolved after non-invasive ventilation. Cerebral ultrasound, video EEG and brain resonance magnetic imaging were performed, but results were normal. Abdominal ultrasound revealed an increased echogenicity of the kidneys and the presence of cysts in the renal medulla, suggesting a renal medullar cystic disease. Anthropometric measurements at that time were 4.590 kg of weight (p‹3), 55 cm of height (p‹3) and a cephalic perimeter of 38 cm (p‹3). At the age of 10 months, his neuropediatrician reported axial hypotonia, unstable sitting, postural plagiocephaly, lower limb hyperreflexia and bilateral inguinal hernia. Weight and height were age appropriate. When reviewed at age 2, he was diagnosed with global developmental delay (mainly characterized by slow development of gross motor milestones and expressive language delay), hypotonia without weakness and nephrocalcinosis. At the age of 3 years, he still showed complete lack of development of speech and certain motor stereotypies. In addition, he underwent orchidopexy for moving his undescended testicles into the scrotum and repairing the associated inguinal hernia (subsequently, this surgical operation has been repeated two more times because it tends to reoccur).

At the age of 6 years, he was hospitalized due to an episode of absence seizure, although this type of episodes have not repeated. EEG was normal and brain resonance magnetic imaging only revealed the presence of partially empty sella turcica, which is being followed-up by his endocrinologist. Currently, he has a severe global developmental delay with no speech, although he is able to understand the speech of others and uses a tablet to communicate needs, wishes or thoughts. He shows multiple stereotypic hand movements. His social behavior is normal and he likes establishing social interactions with children and his parents. At the gross motor level, he has unsteady walk with frequent falls, which is complicated by equinovalgus foot deformity and internal rotation and deformity of the left side of his body, where elevated muscle tone and myotatic reflexes are observed. There is no history of ophthalmological or hearing abnormalities. At the craniofacial level, he has brachycephaly, plagiocephaly with very flat occiput, broad face and midface hypoplasia. Hair on the neck extends more inferiorly than usual. He has synophrys, sparse medial eyebrows, epicanthus, lateral deviation of upslanted palpebral fissures, mildly everted lower eyelids, palpebral ptosis (right eye), anteverted nares, smooth philtrum and carp-like mouth. In addition, he has dental diastema, microdontia, persistent fetal fingertip pads and scoliosis. His current anthropometric measurements are 29 kg of weight (p24, -0.71 SD) and 140 cm of height (p68, 0.47 SD). Prior to his entry in the Spanish Undiagnosed Rare Diseases Program SpainUDP, a Prader-Willi test, an array-CGH (750 k) and a clinical exome panel for neurodevelopmental disorders including 6,110 genes yielded negative results. At the age of 4.8 years, he was admitted into the SpainUDP program and the standard criteria for diagnosis established by this program was performed. Deep phenotyping suggested Kleefstra syndrome as a possible diagnosis.

Proband 3 (P3) is the first child of non-consanguineous Portuguese parents without any medical history. Pregnancy was marked by discovery of congenital cardiopathy (intra ventricular and auricular communication). She was born at term (38 weeks of gestation) with normal parameters: weight was 2824 g (30th p), length was 49 cm (60th p) and occipito-frontal circonference (OFC) was 34 cm (57th p). Neonatal period was marked by acute renal failure with favorable outcome. Ultrasound showed renal asymmetry with an atrophic and cystic left kidney and a right kidney with moderate hyperechogenicity. She also had a hiatal hernia without any complications. Her psychomotor development was normal with independent walk at 11 months and first words at 8 months. She benefited of atrioventricular canal surgery at 4 years old without any complications. At the age of 6.5 years, she presented with suddenly impaired right upper limb function followed by right lower limb function. Consequently, she suffered with numerous falls. Furthermore, she developed progressive dysarthria, paroxysmal movements (hyperkinesia and choreoathetosis of upper limbs) and perioral dyskinesia. Brain MRI was normal as well as etiological assessment (vascular, infectious, inflammatory, autoimmune). At examination, we noticed some dysmorphic features, i.e. synophrys, long eyelashes, hypertelorism. She was treated with L-DOPA with gradual improvement of symptoms. At the age of 7 years (last examination), she doesn`t present with developmental delay or autistic signs. Her cognitive, motor and verbal abilities are normal.

Proband 4 (P4) is a girl born at 39th gestational weeks. Pregnancy was normal and family history was unremarkable. At birth, unilateral postaxial polydactyly of the left foot was noticed. There was facial dysmorphism with frontal bossing, brachycephaly, large anterior fontanelle, short philtrum, and dysplastic cup-shaped and low-set ears. There was a mild pectus excavatum and tapered fingers. There was developmental delay with speech delay, and behavioral anomalies with self-mutilation and aggressivity episodes. The girl walked at age two years. Cardiac examination showed an atrial septal defect. Brain MRI showed prominent folial pattern in the cerebellar vermis. Evolution showed microcephaly (OFC < - 2 SD), moderate deafness, and absent speech.

Proband 5 (P5), a male, is the second child born to healthy unrelated parents. The pregnancy resulted from *in vitro* fertilization, marked by hypoplasia of nasal bones and single umbilical artery at ultrasound and, maternal diabetes during the 3rd trimester. He was born at 41+6 weeks gestation by vaginal delivery with normal birth parameters and an Apgar of 10/10. He presented feeding difficulties in the neonatal period, ventricular muscular septal defect that required surgery at 7 months of age. Posterior urethral valves were surgically repaired at at 20 months of age after an episode of pyelonephritis and febrile seizure. Motor development was delayed as he sat unaided after 9 months and walked unaided at 24 months. He spoke meaningfull words, associated words into sentences and had good social interactions at the age of 2 years. He could swallow and had non-severe chronic constipation. Growth was in the normal range. Facial features involved synophrys, microstomia and crumpled ears. He also had stenosis of one lacrimal duct and frequent mid-ear infections.

Proband 6 (P6) is the second child of healthy, unrelated, white Scottish parents. Antenatal ultrasounds demonstrated a small left ventricle, dilated stomach and duodenum and polyhydramnios. The child was delivered at 39+2 weeks gestation by elective caesarean section. She was born in good condition, with Apgar scores 9 at 1 minute and 9 at 5 minutes. Birthweight was 2840g. Abdomen was distended but soft after birth, with Xray showing distended, gaseous bowel loops. Nonetheless, she passed meconium shortly after birth and a suction rectal biopsy found no evidence of Hirschprung disease. Echocardiogram showed a hypoplastic aortic arch, dysplastic pulmonary valve with mild narrowing and an acute angle turn of both branch pulmonary arteries. At 8 weeks of life, coarctation of the aortic isthmus was repaired by resection and end-to-end anastomosis. The child went on to have significant developmental delay. MRI of brain was reported as unremarkable, except for mild asymmetry of the ventricles and prominence of the extra-axial spaces. EEG at 2 years 7 months revealed abnormal background rhythm (“persistent rhythm 4 Hz theta activity over the temporal lobes. An 8 Hz central mu rhythm was seen while brief passive eye closure evoked a 3.5 Hz posterior rhythm”). Extensive metabolic biochemical investigations were unrevealing. Noisy breathing was noted in early life and sleep studies were abnormal, with clustered desaturations, which persisted despite adenotonsillectomy. She commenced nocturnal BiPAP therapy from around 2.5 years of age. Recurrent abdominal distension has also been noted. Upper gastrointestinal endoscopy and sigmoidoscopy revealed normal mucosa, but evidence of faecal impaction. Symptoms have persisted despite aperients. On most recent review at 3 years 10 months, the child can sit independently and stand with support. She has not spoken any words, but vocalises readily and parents feel these are often meaningful in terms of tone. She can express happiness and enjoys social interactions. There are tongue-clicking stereotypies and stimming behaviors. On examination, she had a brachycephalic head shape with midface retrusion, flat facial profile and prognathism. Head circumference measured 49.2cm (48th centile). Corners of the mouth were downturned, and primary dentition was not fully erupted. Her sclerae appear grey. There was generalised cutis marmorata, and follicular hyperkeratosis affecting the upper arms.

2. Methods of identification of *EHMT2* variants

P1 was ascertained from a series of patients with paediatric-onset rare diseases with unknown genetic basis who underwent whole-exome sequencing. Informed consent for genetic analyses was obtained for all individuals, and genetic studies were performed as approved by the Institutional Review Board of the First Faculty of Medicine of the Charles University, Prague, the Czech Republic. The patient's parents provided written informed consent for the participation in the study, clinical data and specimen collection, genetic analysis and publication of relevant findings.
Genomic DNA extracted from leukocytes of patient and her parents was used for whole-exome sequencing. Exome enrichment was performed on individually barcoded samples using SeqCap EZ MedExome Probes (Roche) and sequencing was performed on Novaseq 6000 platform (Illumina) with 100bp paired-end reads. Reads were aligned to the hg19 reference genome using Novoalign version 3.02.13 (Novocraft) with default parameters.
After genome alignment, conversion of SAM format to BAM and duplicate removal was performed using Picard Tools (2.20.8). The Genome Analysis Toolkit, GATK (3.8)[2] was used for local realignment around indels, base recalibration, variant recalibration, and variant calling. Variants were annotated using the GEMINI framework[3] and filtered based on the population frequencies using several public databases and an in-house database of population-specific variants. Identification of candidate variants was performed for autosomal dominant (de novo variants) and autosomal recessive inheritance patterns. Variants were further prioritized according to the functional impact and conservation score.
Sanger sequencing was used for segregation studies to confirm the variant status in the whole family.

P2 was recruited by the undiagnosed rare diseases program SpainUDP[1] at the Institute of Rare Diseases Research (IIER), Spanish National Institute of Health Carlos III (ISCIII). Peripheral blood samples and skin biopsies were collected from patient and his parents to perform trio-based whole-exome sequencing and to establish fibroblast cultures. Informed consents were signed by the patient's legal representatives. This research project was approved by the ISCIII Research Ethics Committee.
Whole exome sequencing and data analyses were performed in the proband and his unaffected parents. Genomic DNA was extracted from peripheral blood using the Qiagen QIAamp DNA kit. Whole Exome Sequencing (WES) libraries were prepared using the Nimblegen MedExome + ChrMit as enrichment kit and HS2000 v4, 2×100bp sequencing platform in ND095 family and Nextera Felx DNA Library Prep and Illumina NextSeq500 in ND120 family. Data analysis was performed using two different standardized protocols as previously described[1]. These included an *in-house* analysis and a parallel analysis using the Genomic Analysis module of the RD-Connect Genome-Phenome Analysis Platform (GPAP)[4]. All identified rare variants were further analysed, checking all available scientific evidence through detailed searches in public databases (including Gene-Card, NCBI, UniProt, OMIM, Pubmed and ExAc). Sanger sequencing was performed to validate candidate variants.

P3 underwent trio exome sequencing. Libraries of genomic DNA samples were prepared using the Twist Human Core Exome kit (Twist Biosciences, San Francisco, CA), and were sequenced on a NovaSeq 6000 instrument (Illumina, San Diego, CA) according to the manufacturer's recommendations for paired-end 101-bp reads. A mean depth of 93.34 x was reach and 97.1 % of the refseq exons were covered at least by 10 reads.
Variants were identified using a computational platform of the FHU Translad, hosted by the University of Burgundy Computing Cluster (CCuB). Raw data quality was evaluated by FastQC software (v0.11.4). Reads were aligned to the GRCh37/hg19 human genome reference sequence using the Burrows-Wheeler Aligner (v0.7.15). Aligned read data underwent the following processing steps: (a) duplicate paired-end reads were removed by Picard software (v2.4.1), and (b) base quality score recalibration was done by the Genome Analysis Toolkit (GATK v3.8) Base recalibrator. Using GATK Haplotype Caller, Single Nucleotide Variants with a quality score >30 and an alignment quality score >20 were annotated with SNPEff (v4.3t). Rare variants were identified by focusing on nonsynonymous changes present at a frequency less than 1% in the gnomAD database. Copy Number Variants were detected using xHMM (v1.0) and were annotated using in-house python scripts. They were filtered based on their frequency in public databases (DGV, ISCA, DDD).

P4 was recruited by the undiagnosed rare diseases program Solve-RD from ERN ITHACA. Chromosome analysis on lymphocytes, array-CGH (Agilent 105 K) and exome sequencing were normal. Genome sequencing revealed a variant in gene EHMT2 (p.Phe1158Leu).

Whole genome sequencing was performed in **P5** as part of the diagnostic process in France (Plan France Médecine Génomique 2025, PFMG2025) at the genomic laboratory SeqOIA. Genomic DNA was extracted from leukocytes of the patient and his parents using QiaSymphony. Whole genome sequencing libraries were prepared using the mina DNA PCR-free Prep and sequencing was performed on NovaSeq 6000®, Illumina® with 150bp paired-end reads using SBS technology and S4 Flow Cell. Reads were aligned to the GRCh38.92.fa reference genome using bwa-mem2 (v2.2.1). After genome alignment, SNV and indel<50bp were detected using Haplotype caller, GATK (v4.1.9.0). Variants were annotated using the SNPEff (v4.3t) and filtered based on the population frequencies using public databases. Variants were further prioritized according to the inheritance, functional impact and conservation score.

Trio-based whole exome sequencing was performed in **P6** as part of a diagnostic service with the Scottish National Health Service. Libraries were constructed using the Illumina DNA Prep with Enrichment kit and capture was performed using Human Core Exome and Human RefSeq Panel probes from Twist Bioscience. Libraries were sequenced on a NovaSeq 6000 instrument using an S2 flow cell (2x100bp) (Illumina). Initial analysis of the DDG2P gene panel (https://www.ebi.ac.uk/gene2phenotype/panel/DD) using an in-house pipeline revealed no plausible causative variants. Subsequently, prompted by clinical suspicion of Kleefstra syndrome and supportive episignature, targeted analysis was undertaken of EHMT2 using the commercial software platform, Congenica (https://www.congenica.com/). This identified the de novo variant (NM_006709.5:c.3485A>G p.(Lys1162Arg)). Confirmatory Sanger sequencing was performed.

1 McKenna, A. et al. The Genome Analysis Toolkit: a MapReduce framework for analyzing next-generation DNA sequencing data. Genome Res 20, 1297-1303 (2010). https://doi.org:10.1101/gr.107524.110
2 Paila, U., Chapman, B. A., Kirchner, R. & Quinlan, A. R. GEMINI: integrative exploration of genetic variation and genome annotations. PLoS Comput Biol 9, e1003153 (2013). https://doi.org:10.1371/journal.pcbi.1003153
3 López-Martín, E. et al. SpainUDP: The Spanish Undiagnosed Rare Diseases Program. Int J Environ Res Public Health 15 (2018). https://doi.org:10.3390/ijerph15081746
4 Laurie, S. et al. The RD-Connect Genome-Phenome Analysis Platform: Accelerating diagnosis, research, and gene discovery for rare diseases. Hum Mutat 43, 717-733 (2022). https://doi.org:10.1002/humu.24353

Uncropped scans of blots from Supplementary Figures:

Supplementary Figure 3A:

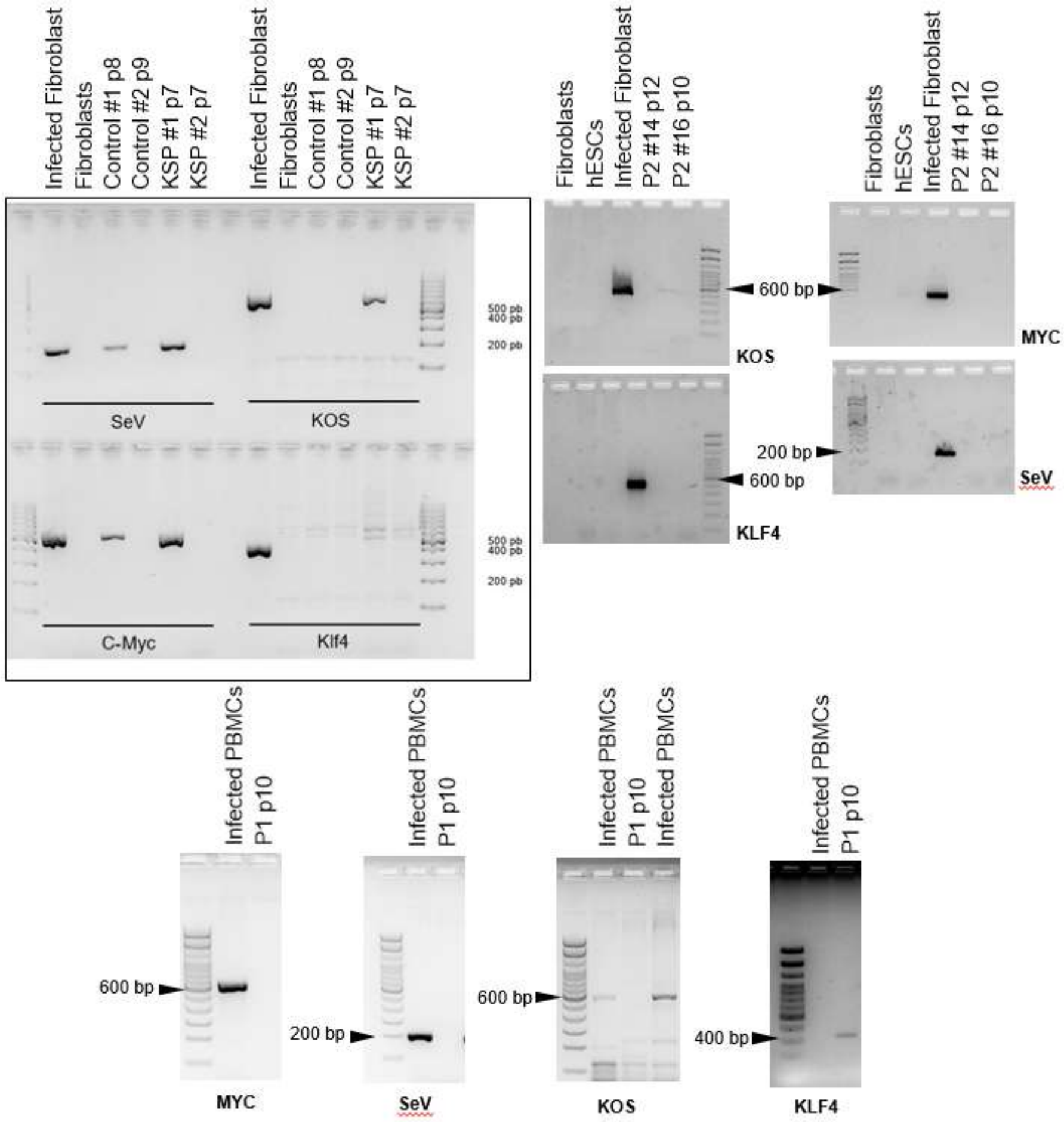

Supplementary Figure 9C:

Whole cell extracts : iPSCs and fibroblast

anti-GLP

Chemiluminescence channel

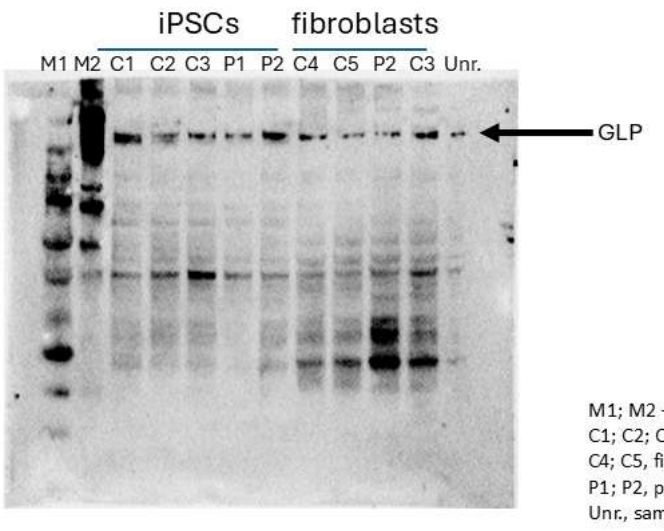

Colorimetric channel

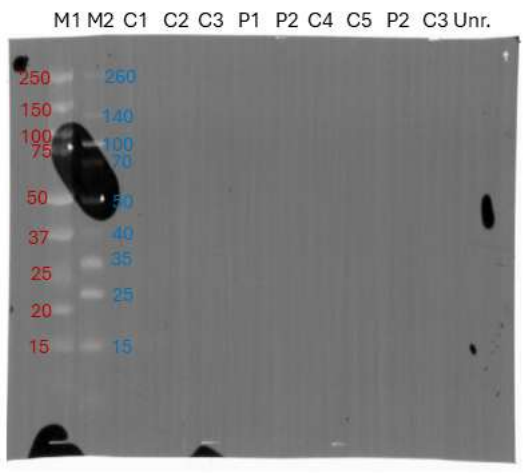

M1; M2 – MW markers
C1; C2; C3, iPSCs control
C4; C5, fibroblast control
P1; P2, patients
Unr., sample unrelated to this study

Whole cell extracts : iPSCs and fibroblast

anti-GAPDH control for GLP immunodetection

Chemiluminescence channel

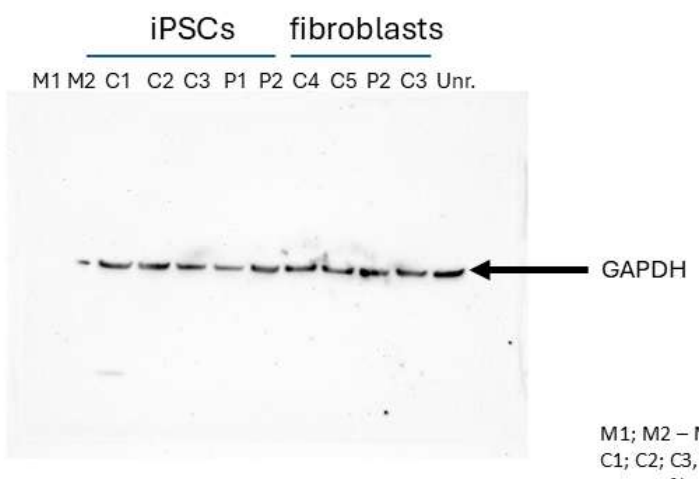

Colorimetric channel

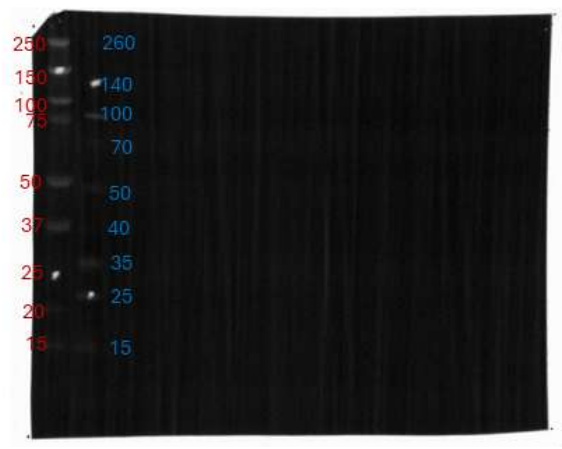

M1; M2 – MW markers
C1; C2; C3, iPSCs control
C4; C5, fibroblast control
P1; P2, patients
Unr., sample unrelated to this study

Whole cell extracts : iPSCs and fibroblast

anti-G9a

Chemiluminescence channel

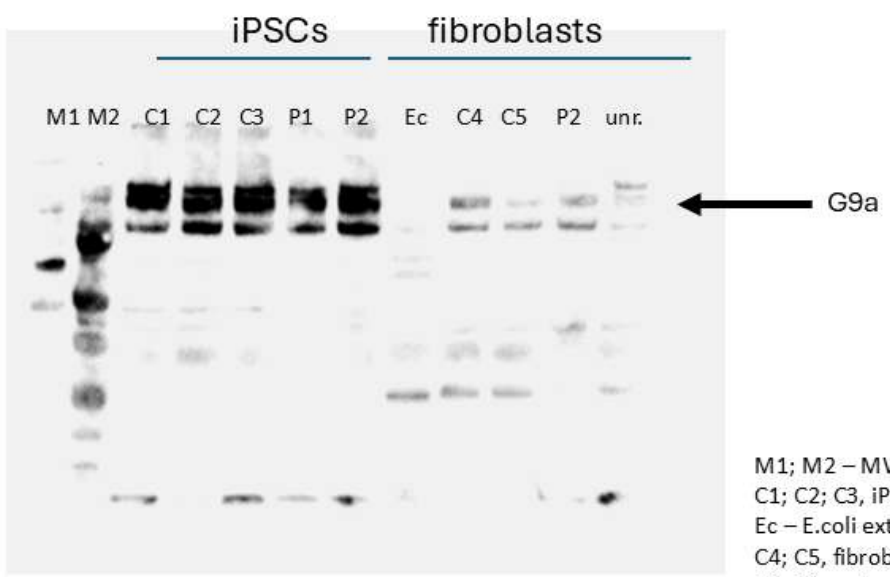

Colorimetric channel

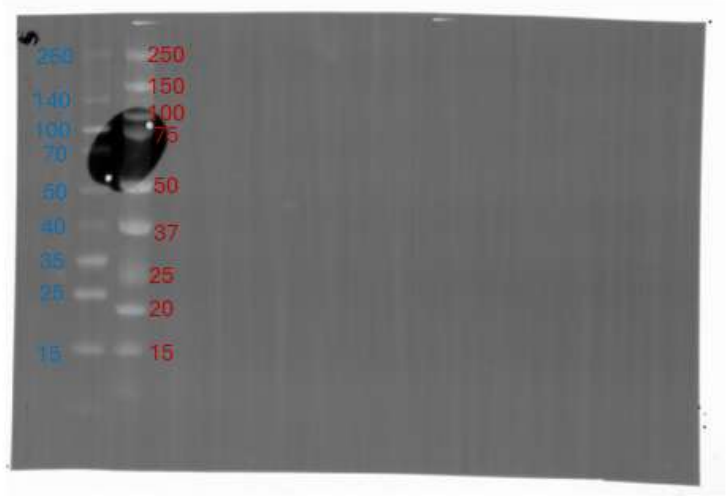

M1; M2 – MW markers
C1; C2; C3, iPSCs control
Ec – E.coli extract
C4; C5, fibroblast control
P1; P2, patients
Unr., sample unrelated to this study

Whole cell extracts : iPSCs and fibroblast

anti-GAPDH control for G9a immunodetection

Chemiluminescence channel

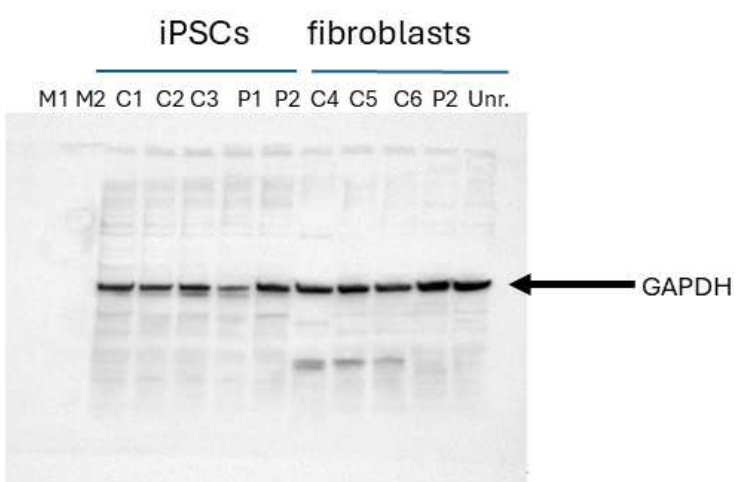

Colorimetric channel

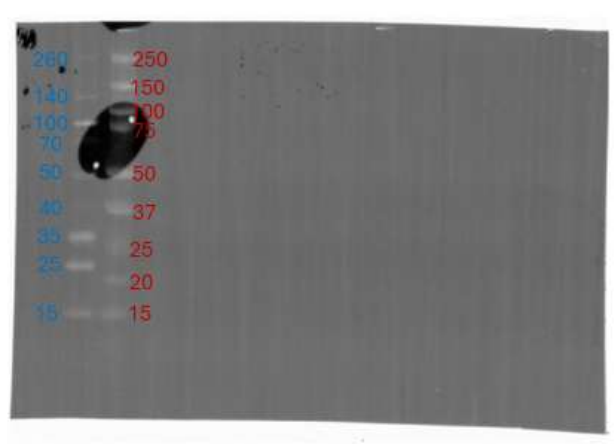

M1; M2 – MW markers
C1; C2; C3, iPSCs control
C4; C5, fibroblast control
P1; P2, patients
Unr., sample unrelated to this study

<u>Supplementary Figure 9D:</u>

Immunoprecipitation of G9a in iPSCs

G9a immunodetection

Chemiluminescence channel

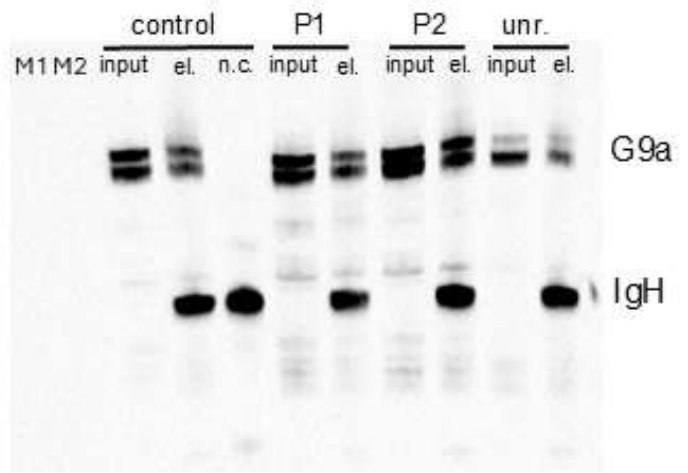

Colorimetric channel

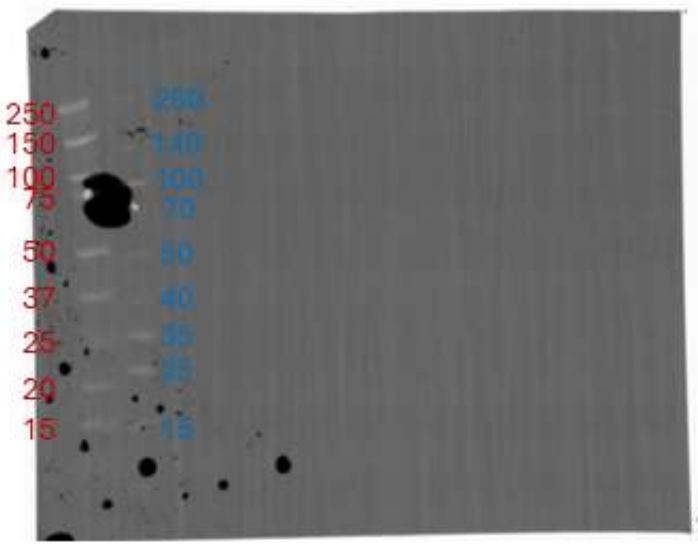

M1; M2 – MW markers
P1; P2, patients
Unr., sample unrelated to this study
El. means elution

Immunoprecipitation of G9a in iPSCs

GLP immunodetection

Chemiluminescence channel

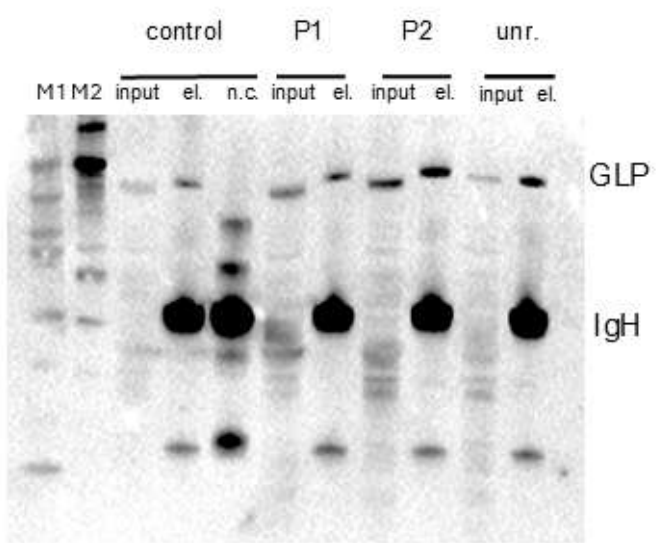

Colorimetric channel

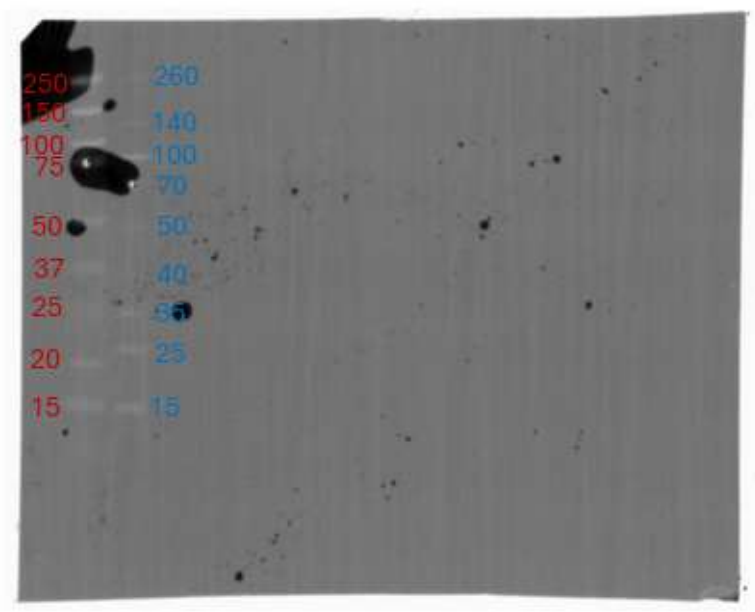

M1; M2 – MW markers
P1; P2, patients
Unr., sample unrelated to this study
El. means elution

<u>Supplementary Figure 14A:</u>

Solubility of GLP mutants upon expression in *E.coli*

His-tag immunodetection

Chemiluminescence channel

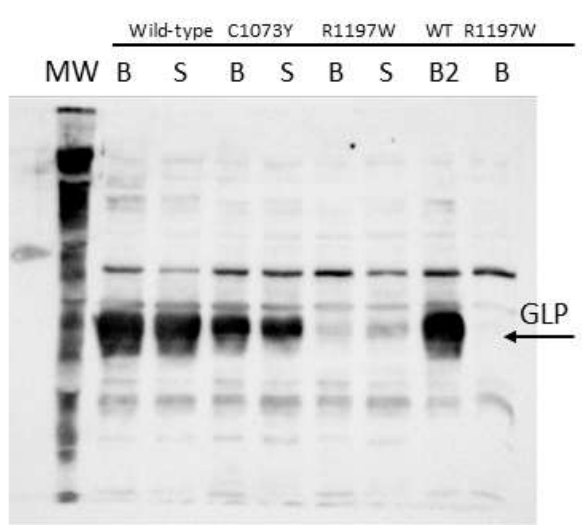

Colorimetric channel

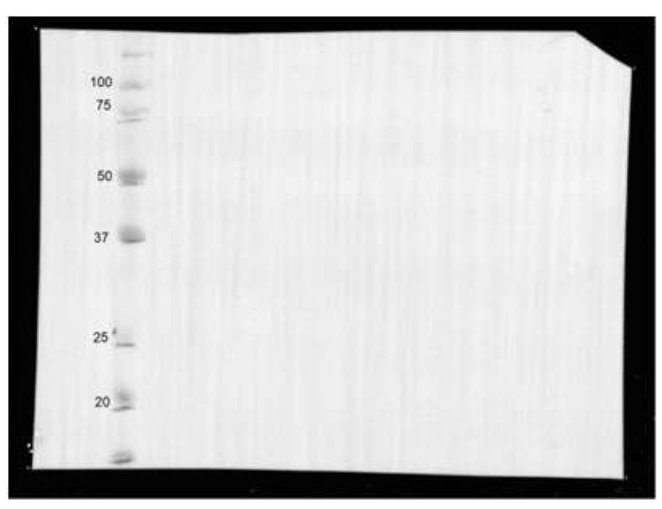

MW – molecular weight marker
B – bug buster extraction (10 µg of total protein)
B2 – half load of bug buster extraction (5 µg of total protein)
S – sonication extract (10 µg of total protein)

Supplementary Figure 14A:

Insoluble fraction containing GLP mutants upon expression in *E.coli*

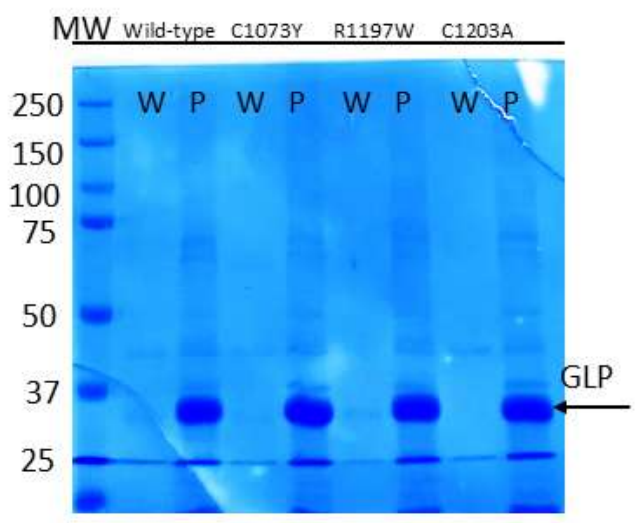

MW – molecular weight marker
W – wash of insoluble fraction
P – pellet solubilized by heating
