# Supplementary material for: De novo EHMT2 variants cause an autosomal dominant EHMT2-related Kleefstra syndrome via loss of G9a methyltransferase activity
Source: Nat Commun. 2026 Jul 1;17:8203. doi: 10.1038/s41467-026-74987-w (PMC13462943; doi:10.1038/s41467-026-74987-w)
Supplement: Supplementary file 2 [file 41467_2026_74987_MOESM2_ESM.pdf]

**Description of Additional Supplementary Files**

**Supplementary Data 1:** Clinical data of patients with EHMT2 variants.

**Supplementary Data 2:** The mean methylation difference between patients and controls. Empirical Bayes moderation was applied, and adjusted p-values were calculated using the Benjamini-Hochberg method to control the false discovery rate.

**Supplementary Data 3:** Quantification of histone modifications in fibroblasts and iPSCs lines by mass spectrometry.

The percent relative abundance is shown in triplicates. Mean and standard deviation of triplicates is indicated. Statistical significance for pairwise comparisons was assessed using a two-sided Welch's t-test. Exact P values are reported.

**Supplementary Data 4:** MS-based identification of immunoprecipitates (iPSCs extract against antiG9a): top 100 hits with -10lgP higher than 100, and not identified in the negative control. Hits relevant to GLP-G9a complex are highlighted with bold font.

The log(p) values were generated by the PEAKS software using a log-transformation (i.e., $\log_{10}$) of p-values that quantify the probability of random peptide–spectrum matches; higher values indicate more confident identifications.

**Supplementary Data 5:** List of SHIRPA scores in individual mice

**Supplementary Movie 1**: Representative ventral view video illustrating the altered and unstable gait pattern observed in Ehmt2+/del_1076- 1079 mutant mice.

**Supplementary Movie 2**: Ehmt2+/+male animal as a comparison to Ehmt2+/del_1076-1079 male in Supplementary Movie 1. Ventral view video of typical Ehmt2+/+ mouse gait pattern.
