# Supplementary material for: De novo EHMT2 variants cause an autosomal dominant EHMT2-related Kleefstra syndrome via loss of G9a methyltransferase activity
Source: Nat Commun. 2026 Jul 1;17:8203. doi: 10.1038/s41467-026-74987-w (PMC13462943; doi:10.1038/s41467-026-74987-w)
Supplement: Supplementary file 3 [file 41467_2026_74987_MOESM6_ESM.pdf]

nature portfolio

Corresponding author(s): Lenka Noskova, Maria J. Barrero

Last updated by author(s): Apr 16, 2026

Reporting Summary

Nature Portfolio wishes to improve the reproducibility of the work that we publish. This form provides structure for consistency and transparency in reporting. For further information on Nature Portfolio policies, see our Editorial Policies and the Editorial Policy Checklist.

Statistics

For all statistical analyses, confirm that the following items are present in the figure legend, table legend, main text, or Methods section.

n/a	Confirmed	
☐	☒	The exact sample size (*n*) for each experimental group/condition, given as a discrete number and unit of measurement
☐	☒	A statement on whether measurements were taken from distinct samples or whether the same sample was measured repeatedly
☐	☒	The statistical test(s) used AND whether they are one- or two-sided<br>*Only common tests should be described solely by name; describe more complex techniques in the Methods section.*
☐	☒	A description of all covariates tested
☐	☒	A description of any assumptions or corrections, such as tests of normality and adjustment for multiple comparisons
☐	☒	A full description of the statistical parameters including central tendency (e.g. means) or other basic estimates (e.g. regression coefficient) AND variation (e.g. standard deviation) or associated estimates of uncertainty (e.g. confidence intervals)
☐	☒	For null hypothesis testing, the test statistic (e.g. *F*, *t*, *r*) with confidence intervals, effect sizes, degrees of freedom and *P* value noted<br>*Give P values as exact values whenever suitable.*
☒	☐	For Bayesian analysis, information on the choice of priors and Markov chain Monte Carlo settings
☐	☒	For hierarchical and complex designs, identification of the appropriate level for tests and full reporting of outcomes
☐	☒	Estimates of effect sizes (e.g. Cohen's *d*, Pearson's *r*), indicating how they were calculated

Our web collection on statistics for biologists contains articles on many of the points above.

Software and code

Policy information about availability of computer code

Data collection	The following softwares were used: Illumina software for DNA sequencing and Illumina GenomeStudio for EPIC array, MGI DNBSEQ platform software for RNA sequencing, Active Motif software for mass spectrometry data, Autoflex Speed software for MALDI-TOF MS-based methyltransferase assay, CFX96 Real-Time System software for differential scanning fluorimetry, Waters Synapt G2Si instrument software and MassLynx 4.1 Spectrometry software for native mass spectrometry, Spark plate reader (TECAN) software for biolumiscence-based methyltransferase assay, SkyScan 1278 software for microCT of mouse skulls, VevoLab software (FUJIFILM VisualSonics Inc.)
Data analysis	The following softwares were used: DNA and RNA sequencing: Novoalign version 3.02.13 (Novocraft), Picard Tools (2.20.8), GATK - more versions, Gemini framework 0.16.0, RD-Connect Genome-Phenome Analysis Platform, FastQC software (v0.11.4), Burrows-Wheeler Aligner (v0.7.15), Picard software (v2.4.1), GATK Haplotype Caller (v4.1.9.0), SNPEff (v4.3t), xHMM (v1.0), bwa-mem2 (v2.2.1), Congenica commercial software platform, episignature analysis: R statistical software version 4.4.1 - packages SeSAMe, matchIt, limma, ggplot2, ggplots, e1071, caret. RNA analysis: Galaxy, STAR mapper, R packages HTseq-count, DEseq, DESeq2, SRPlot, DAVID, Morpheus, mass spectrometry: Skyline, structural mapping: Pymol Viewer 2.1 (Schrodinger, LLC.), UCSF ChimeraX, methyltransferase assay: OriginPro, MALDI-TOF assay: FlexAnalysis 3.4., native mass spectrometry: UniDec v6.0.3, mouse model generation and testing: DECODR.org online software, NRecon software, version 2.2.0.6, CTvox software, version 3.3.0.0, DataViewer, version 1.5.2, Viewer software 3.0.1.452., PEAKS 12.5 software, DigiGait Imaging and Analysis software ver. 16 (Mouse Specifics Inc., Framingham, MA, USA).

For manuscripts utilizing custom algorithms or software that are central to the research but not yet described in published literature, software must be made available to editors and reviewers. We strongly encourage code deposition in a community repository (e.g. GitHub). See the Nature Portfolio guidelines for submitting code & software for further information.

Data

Policy information about availability of data

All manuscripts must include a data availability statement. This statement should provide the following information, where applicable:

- Accession codes, unique identifiers, or web links for publicly available datasets
- A description of any restrictions on data availability
- For clinical datasets or third party data, please ensure that the statement adheres to our policy

The pathogenic variants detected in our study have been submitted to ClinVar (https://www.ncbi.nlm.nih.gov/clinvar/), submission numbers SCV004231847, SCV006279706 to SCV006279710 and SCV007518740. Native MS data are available in ZENODO under https://doi.org/10.5281/zenodo.19630380. Mass spectrometry data from histone extracts are available in MassIVE repository under https://proteomecentral.proteomexchange.org/cgi/GetDataset?ID=PXD079241. Proteomic data from immunoprecipitation experiments are available in Pride (Project Number PXD076523). List of differentially methylated probes, histone modification data, thermostability data and kinetic data are provided in Supplementary Tables and Supplementary Data. Source data file is provided with the paper. RNA sequencing data are available at the European Genome-phenome Archive under accession https://ega-archive.org/studies/EGAS50000001637. Because of the Personal Information Protection Law, individual participant DNA sequencing data beyond that reported in this article are under regulation by the individual institutional review boards. Datasets for episignature analyses used in this study that are available publicly are previously described. The individual genomic and epigenomic or any other personally identifiable data for other samples in the EpiSign Knowledge Database (EKD) are prohibited from deposition in publicly accessible databases due to institutional and ethics restrictions. Specifically, these include data and samples submitted from external institutions to London Health Sciences EKD that are subject to Institutional Material and Data Transfer agreements, data submitted to London Health Sciences for episignature assessment under Research Services Agreements, and research study cohorts under Institutional Research Ethics Approval (Western University REB 106302; and REB 116108). Some of the software packages used in this study are publicly available as described in the Materials and Methods. EpiSign™ is a commercial software and is not publicly available.

Research involving human participants, their data, or biological material

Policy information about studies with human participants or human data. See also policy information about sex, gender (identity/presentation), and sexual orientation and race, ethnicity and racism.

Reporting on sex and gender	The study presents a cohort of seven patients affected by a (ultra)rare monogenic disorder. Their biological sex (male vs. female) is reported as provided by the managing physicians. Gender wasn't considered based on medical history of the patients (see neuropsychiatric history in the manuscript).
Reporting on race, ethnicity, or other socially relevant groupings	Similar to previous, race, ethnicity or other socially relevant groupings are not part of the study given the (ultra)rare nature of the condition.
Population characteristics	Not applicable - study of cohort of seven patients with (ultra)rare disease. Variant population frequencies referenced in the manuscript originate from publicly available databases (gnomAD).
Recruitment	The cohort was established based on personal communication of the authors (GeneMatcher, RD-connect platforms).
Ethics oversight	The study adheres to the principles set out in the Declaration of Helsinki. The study was approved by the corresponding author's institutional ethical review board (Ethics Committee of the General University Hospital in Prague, no. 34/22).

Note that full information on the approval of the study protocol must also be provided in the manuscript.

Field-specific reporting

Please select the one below that is the best fit for your research. If you are not sure, read the appropriate sections before making your selection.

☒ Life sciences ☐ Behavioural & social sciences ☐ Ecological, evolutionary & environmental sciences

For a reference copy of the document with all sections, see nature.com/documents/nr-reporting-summary-flat.pdf

Life sciences study design

All studies must disclose on these points even when the disclosure is negative.

Sample size	The study included seven patients with specific rare variants in EHMT2 gene.
Data exclusions	No data were excluded from the analyses.
Replication	Strategies used to establish technical and/or biological replicates differed based on the individual analyses/methods. Details are provided in the Methods and Supplementary Materials.
Randomization	Not applicable given the size of the cohort.
Blinding	Not applicable for patients given the size of the cohort. Evaluators were blinded to the genotype of individual mice.

Reporting for specific materials, systems and methods

We require information from authors about some types of materials, experimental systems and methods used in many studies. Here, indicate whether each material, system or method listed is relevant to your study. If you are not sure if a list item applies to your research, read the appropriate section before selecting a response.

Materials & experimental systems

n/a	Involved in the study	
☐	☒	Antibodies
☐	☒	Eukaryotic cell lines
☒	☐	Palaeontology and archaeology
☐	☒	Animals and other organisms
☐	☒	Clinical data
☒	☐	Dual use research of concern
☒	☐	Plants

Methods

n/a	Involved in the study	
☒	☐	ChIP-seq
☒	☐	Flow cytometry
☒	☐	MRI-based neuroimaging

Antibodies

Antibodies used	anti-Sox2 AB5603 Sigma and PA1-094 Fisher Scientific (1:400), anti-NANOG AF1997-SP R&D (1:50), anti-Oct3/4 (C-10) sc-5279 Santa Cruz Biotechnology, anti-TRA-1-60 sc-21705 Santa Cruz Biotechnology, anti-Alpha-1-fetoprotein A000829-2 Agilent Dako (1:500), Alpha-Smooth Muscle A5228 Sigma (1:500), TUBB3 Biolegend 801202(1:500), G9a (rabbit anti-G9a, 1:1000, Sigma, G 6919), GAPDH (mouse Anti-GAPDH, 1:10000, Sigma G8795), Anti-6X His tag primary antibody (ab9108 from Abcam), Rabbit IgG (H+L) Secondary Antibody (31460 from ThermoFischer Scientific), GLP (rabbit anti-GLP, 1:1000, Sigma, 09-078), rabbit anti-G9a (ab185050, Abcam), rabbit anti-PFAS IgG, ARP46181 Aviva Systems Biology)
Validation	All antibodies were validated and compared with product data sheets. Expected protein size and cell localization corresponded to the available information provided by the manufacturers. No custom-made antibodies were used. The samples omitting the primary antibody was used as a control (see manuscript for further details).

Eukaryotic cell lines

Policy information about cell lines and Sex and Gender in Research

Cell line source(s)	Primary fibroblast cultures from patient P2 (XY), control (XY) and Kleefstra syndrome patient (XX), iPSCs from patient P1 (XX), patients P2(XY) and controls (XY and XX) were used in the study.
Authentication	Genotypes of the primary fibroblast cultures were established by sequencing of EHMT1 and/or EHMT2 variants. iPSCs differentiation protocols are described in detail in the manuscript.
Mycoplasma contamination	All cell lines used in the study tested negative for mycoplasma.
Commonly misidentified lines (See ICLAC register)	N/A

Animals and other research organisms

Policy information about studies involving animals; ARRIVE guidelines recommended for reporting animal research, and Sex and Gender in Research

Laboratory animals	C57BL/6NCrl mice
Wild animals	Not applicable
Reporting on sex	Animals of both sexes (males and females) and genotypes (heterozygous knock-ins vs. wild type littermates) were analyzed. Comparisons were made between the two genotypes separately for each sex. If not statistically different between sexes, the data for each of the genotypes was pooled (see manuscript for details). Sex of the animals was established by postnatal visual inspection and genotyping.
Field-collected samples	Not applicable
Ethics oversight	The animal study was approved by the Animal Care and Use Committee of the Institute of Molecular Genetics (AVCR 7341/2021 SOV II, AVCR 6144/2022 SOV II) and conducted in accordance with national and EU guidelines for the care and use of laboratory animals.

Note that full information on the approval of the study protocol must also be provided in the manuscript.

Clinical data

Policy information about clinical studies

All manuscripts should comply with the ICMJE guidelines for publication of clinical research and a completed CONSORT checklist must be included with all submissions.

Clinical trial registration	N/A
Study protocol	N/A
Data collection	Informed consent for genetic analyses was obtained for all individuals, and genetic studies were performed as approved by the review boards of the participating institutions. The patients' parents or legal guardians provided written informed consent for the participation in the study, clinical data and specimen collection, genetic analyses and publication (patients photos included) of relevant findings. All data were de-identified.
Outcomes	N/A

Plants

Seed stocks	N/A
Novel plant genotypes	N/A
Authentication	N/A
