# Supplementary material for: De novo EHMT2 variants cause an autosomal dominant EHMT2-related Kleefstra syndrome via loss of G9a methyltransferase activity
Source: Nat Commun. 2026 Jul 1;17:8203. doi: 10.1038/s41467-026-74987-w (PMC13462943; doi:10.1038/s41467-026-74987-w)
Supplement: Supplementary file 4 [file 41467_2026_74987_MOESM7_ESM.pdf]

nature portfolio

Peer Review File

De novo *EHMT2* variants cause an autosomal dominant *EHMT2*-related Kleefstra syndrome via loss of G9a methyltransferase activity

Corresponding Author: Dr Lenka Noskova

This file contains all reviewer reports in order by version, followed by all author rebuttals in order by version.

Version 0:

Reviewer comments:

Reviewer #1

(Remarks to the Author)
In this manuscript, the authors present strong evidence that pathogenic de novo variants in EHMT2 correlate with phenotypic features very similar to patients with Kleefstra Syntrome, which is caused by EHMT1 haploinsufficiency. EHMT1 and EHMT2 encode GLP and G9a, respectively, which functionally interact to catalyze the methylation of H3K9. In contrast to clinical EHMT1 loss-of-function variants, the authors show mutations in EHMT2 result in stable, but catalytically inactive G9a enzyme. The de novo variants in EHMT2 lead to similar, but identifiably distinct, epi-signatures compared to Kleefstra Syndrome in cell lines. In addition a mouse model for one of the variants (Ehmt2+/del_1076-1079) demonstrates phenotypes associated with Kleefstra Syndrome. The authors state the mechanism likely differs from that of the haploinsufficiency of EHMT1, and suggest a dominant negative mechanism. Overall, the data in the manuscript strongly support the causality of the variants and the mechanisms proposed. However, which the “dominant negative” mechanism is plausible, the study lacks the in vitro experiments in live cells to formally address the relative role of heterodimers and homodimers.

1. The authors state “Overall, our observations indicate that mutations in EHMT1 and EHMT2 affect the same epigenetic module, albeit with specific functional differences.”
Methylation patterns of EHMT2 variants resemble episignatures of EHMT1 related Kleestra Syndrome, measured by a MVP score. However, there was some uniqueness in the episignatures, highlighted by differences in histone modifications compared to Kleefstra Syndrome samples included in the study as well as published studies. Despite differences in histone modifications, changes in gene expression and DAVID enrichments were similar across P2 and KSP fibroblasts and P1, P2, and KSP iPSCs. The authors highlighted shared differentially expressed genes and enrichment terms but did not do any DAVID enrichment/GO term enrichment for differentially expressed genes not shared between EHMT2 and EHMT1 mutations. This may speak to further functional differences between the mutations and the pathogenic mechanisms.

2. The authors describe a new de novo mutation in EHMT2 discovered by having the same episignature (Patient 7). However, they rule the variant likely benign due to the variant being outside of the methyltransferase domain and having normal methyltransferase activity and protein stability. The authors do not discuss potential reasons why the patient has a matching episignature and clinical phenotypes similar to Kleefstra Syndrome. I think this highlights a weakness in the idea that this episignature is unique to EHMT2 variants that genocopy Kleefstra Syndrome and could be used for identifying candidates. The manuscript would benefit from further discussion or investigation of other variants the patient may have that is driving the episignature.

3. The manuscript suggests that the pathogenic mechanism of EHMT2 variants differs from the haploinsufficiency observed in EHMT1 variants. However, this distinction remains unclear. Given that EHMT2 variants still produce a stable G9a protein capable of forming heterodimers with GLP, one could argue that these variants may deplete the pool of GLP proteins available for forming functional heterodimers. This scenario could mimic the effects of EHMT1 haploinsufficiency, where a reduction in functional heterodimers contributes to disease pathology. A major limitation of this study is all analysis of the GLP/G9a interaction is performed using purified protein, rather than an in vitro cellular system. Thus, if the authors propose that EHMT1 homodimers compensate for the loss or reduction of functional heterodimers, it would strengthen the argument to demonstrate an increase in EHMT1 homodimer formation in patient fibroblasts or other cell line.

Additionally, if GLP-G9a heterodimers are preferentially formed and have higher catalytic activity compared to G9a homodimers, this could potentially explain the milder phenotypes observed in G9a+/– mice relative to the Ehmt2+/del_1076-1079 variant, where the GLP pool is not depleted by non-functional heterodimers. Clarifying this point with supporting biochemical or functional data would strengthen the mechanistic interpretation.

4. The observation that approximately 50% of the mice in this Ehmt2+/del_1076-1079 model died between P30–40 is interesting. However, all phenotyping analyses were conducted on animals that survived beyond this timepoint. To more comprehensively characterize the model and similarities to Kleefstra Syndrome, I would recommend including assessments of mice during prior to the P30-40 timepoint. This could help uncover early-onset phenotypes and provide insight into potential causes of lethality. Specifically, evaluating cardiac anomalies or more pronounced craniofacial abnormalities may reveal underlying defects contributing to early mortality. The potential identification of cardiac anomalies would also strengthen the model for Kleefstra Syndrome.

Typo:
In the body of the manuscript the variant a mouse model was generated for was reported to be the Ehmt2+/del_1076-1079 (line 323), but thereafter it is referred to as Ehmt2+/del_1076-1076 (line 329, 331 etc).

Reviewer #2

(Remarks to the Author)
I co-reviewed this manuscript with one of the reviewers who provided the listed reports. This is part of the Nature Communications initiative to facilitate training in peer review and to provide appropriate recognition for Early Career Researchers who co-review manuscripts.

Reviewer #3

(Remarks to the Author)
Key results:
This paper describes novel mutations in the EHMT2 substrate binding site in patients exhibiting similar symptoms to Kleefstra syndrome. The authors show that the patients have a specific episignature, distinct from Kleefstra, and other similar syndromes. The authors provide a number of analyses to show that these mutations lead to functional decreases in enzymatic activity, and provide further data to show that these are either due to a lack or reduction of binding to H3, or SAM. They generated a new mouse model to demonstrate that one of these mutations is sufficient to recapitulate some physical and developmental problems observed in the patient, something that has not been observed previously with heterozygous loss of Ehmt2. Based on these findings, they propose a plausible model suggesting that these mutations function in a dominant negative manner to reduce the activity of the EHMT1/2 heterodimeric complex on chromatin.
Validity:
For the most part the data interpretation and conclusions drawn are sensible in light of the data presented and references drawn upon. There are a couple of overreaches in interpretation, which I have highlighted in the suggested improvements below.
Significance:
This paper makes an important contribution to the field by providing the first evidence of an EHMT2 syndrome caused by potential dominant negative mutations in EHMT2. Some minor edits would increase clarity as suggested below.
Data and methodology:
Changes to histone methylation were associated with changes in gene expression in cell lines generated from patients. Appropriate healthy controls and KSP controls were used. Multiple different functional assays were used to convincingly demonstrate a change in enzyme activity caused by the novel mutations, including direct measures of changes to histone methylation. The use of the animal model to demonstrate Ehmt2 mutations as causative of features of the syndrome was appropriate and added strength to the conclusions, however, it was underutilised and demonstration of some key developmental anomalies seen in patients would significantly strengthen their conclusions. I have highlighted in the suggested improvements where I feel the data presented could be improved to increase clarity or confidence in the data presented.
Analytical approach
There is no mention of numbers of experimental or technical replicates for many of the experiments. The numbers of replicates, sex and statistical tests applied should be stated explicitly in the figure legends. No statistical test appears to have been applied to the assay measuring methyltransferase activity – likely due to the high number of assays which had no detectable activity. Number of replicates was also not reported for data reported in this figure (Fig 5), making it hard to evaluate how robust this data is. This should be reported, and ideally individual replicates should be plotted as datapoints on the graph to increase transparency. The animal data presented is more robust, with a sufficient number of replicates (as made visible by individual data points being plotted), and proper attention paid to potential sex differences. However, number of animals used per group is not reported in the main text or the figure legend and should be visible in at least one of these places. Methods should explicitly state whether researchers were blind to genotype, especially for measuring craniofacial features reported in Fig. 6D.

Clarity and context:
The manuscript is clearly written with good inclusion of previous work to understand context.

References:
The authors have used appropriate references. The addition of a reference should be included to support the speculation on line 222 that the genes regulated in the variants are direct targets of G9a if one is available. If not, this statement should be supported by experimental data.

Suggested improvements:
1. The numbers of replicates, sex and statistical tests applied should be stated explicitly in all figure legends.
2. Fig. 3C is too high level to be sufficiently informative. I suggest replacing it with a more informative plot e.g. Suppl. Fig 6A,B.
3. From my understanding of the design of the dimerization assays used, these were measures of homodimer formation – is data available for rate of heterodimer formation? I ask because as the authors state in the introduction, heterodimers are the preferred form in vivo with maximal activity. Ratio of hetero:homo dimer formation with the variant compared to a control may also be biologically interesting if the data is available (refers to Fig 5B and Supp Fig. 12).
4. Methods should explicitly state whether researchers were blind to genotype, especially for measuring craniofacial features reported in Fig. 6D.
5. To validate the mouse model the authors assess the mice by SHIRPA, show reduced activity and cranioskeletal changes. These are insufficient to draw the conclusion that the mice represent a good model for EHMT2 Kleefstra syndrome. Can the authors assess and report the incidence of a couple of other key features such as the incidence of cardiac and kidney/urogenital anomalies characteristic of the condition? These are relatively straight-forward to assess and score and will substantially increase confidence in this conclusion.
6. The description and interpretation of the DNAme analyses should be improved and discussed. The episignature data presented (e.g. in Fig. 2B) shows that EHMT2 syndrome samples group together, distinct from “classical” Kleefstra syndrome and controls. However, in the summary the authors state that episignatures are similar to Kleefstra syndrome. Can the authors clarify these interpretations and statements?

Minor edits:
7. Supplementary figure 1 legend: at the end of this there appears to be a sentence copied from the supplementary figure 3 legend.
8. Inclusion of all the differential methylation data is appreciated but addition of a visual summary of supplementary table 1 would be appreciated for ease of interpretation.
9. Inclusion of negative controls would increase confidence in the immunofluorescent staining shown in Supplementary figure 4.
10. Line 208. Please reference the papers describing the established standard of iPSC culture generation & definition.
11. Fig 6B – please insert statistics.
12. Methods section for solubility of GLP requires revisions for typos.
13. Please make breakdown of SHIRPA test available as a supplementary table.
14. Line 347-348: As this was not statistically significant this feels like an overinterpretation, especially as the difference between the Δ is not large. The labelling of supplementary fig 23 is not clear and there are typos in the legend.
15. Supplementary figure 25 legend: typos.
Expertise:
Mouse models of chromatin disorders.

Reviewer #4

(Remarks to the Author)
I co-reviewed this manuscript with one of the reviewers who provided the listed reports. This is part of the Nature Communications initiative to facilitate training in peer review and to provide appropriate recognition for Early Career Researchers who co-review manuscripts.

Reviewer #5

(Remarks to the Author)
The authors are to be congratulated on an important report of a novel disease causing gene and its associated phenotype. The authors provide clear functional evidence for the reported variants in EHMT2. Functional studies are convincing and well explained. The patients are described in appropriate detail in the table and the provided photographs.
Particularly interesting is the distinctive episignature, which identified an additional patient (P7).

Specific comments:
- Why is the 7th patient not counted in the total number of cases in the abstract? It should be included.
- In the title and throughout the manuscript the term "genocopy" is used. While the term "phenocopy" is used for a condition that looks just like another condition, a similar term "genocopy" is not commonly used. The authors should decide what they consider this newly reported condition. Is this EHMT2-related neurodevelopmental disorder? Is it EHMT2-related Kleefstra syndrome? Is it EHMT2-related Kleefstra syndrome type 2? Once this decision is made, the manuscript should be carefully edited for consistency. It is at times unclear when the term Kleefstra syndrome is used if this refers to EHMT1-related Kleefstra syndrome or the novel condition or a combination of these. For example in the figure showing the effects of the

variants in the different genes, both are referred to as "Kleefstra syndrome". In summary, the messaging regarding the phenotype and its naming needs to be tightened and explained, then applied to the entire manuscript.
- For clinicians, the facial features are important in order to identify the patients. The authors did well in showing several patients. If additional photographs of the 7th patient can be shown, please include these.
- The usefulness of the episignature analysis, potentially in combination with genome or exome analysis, should be stressed (even more). This newer tool is underutilized and should become a standard in the evaluation of undiagnosed patients with neurodevelopmental disorders.

Version 1:

Reviewer comments:

Reviewer #1

(Remarks to the Author)
The authors have provided thorough and thoughtful responses to my critique. I have no further concerns.

Reviewer #2

(Remarks to the Author)
I co-reviewed this manuscript with one of the reviewers who provided the listed reports. This is part of the Nature Communications initiative to facilitate training in peer review and to provide appropriate recognition for Early Career Researchers who co-review manuscripts.

Reviewer #3

(Remarks to the Author)
The authors have addressed all the points raised in our review and I am happy to recommend acceptance of the revised manuscript.

Reviewer #4

(Remarks to the Author)
I co-reviewed this manuscript with one of the reviewers who provided the listed reports. This is part of the Nature Communications initiative to facilitate training in peer review and to provide appropriate recognition for Early Career Researchers who co-review manuscripts.

Reviewer #5

(Remarks to the Author)
This reviewer's concerns have been addressed, though the term "genocopy" is still used (line 556) in the text.

Open Access This Peer Review File is licensed under a Creative Commons Attribution 4.0 International License, which permits use, sharing, adaptation, distribution and reproduction in any medium or format, as long as you give appropriate credit to the original author(s) and the source, provide a link to the Creative Commons license, and indicate if changes were made.
In cases where reviewers are anonymous, credit should be given to 'Anonymous Referee' and the source.
The images or other third party material in this Peer Review File are included in the article's Creative Commons license, unless indicated otherwise in a credit line to the material. If material is not included in the article's Creative Commons license and your intended use is not permitted by statutory regulation or exceeds the permitted use, you will need to obtain permission directly from the copyright holder.
To view a copy of this license, visit https://creativecommons.org/licenses/by/4.0/

REVIEWER COMMENTS

Reviewer #1 (Remarks to the Author):

In this manuscript, the authors present strong evidence that pathogenic de novo variants in EHMT2 correlate with phenotypic features very similar to patients with Kleefstra Syntrome, which is caused by EHMT1 haploinsufficiency. EHMT1 and EHMT2 encode GLP and G9a, respectively, which functionally interact to catalyze the methylation of H3K9. In contrast to clinical EHMT1 loss-of-function variants, the authors show mutations in EHMT2 result in stable, but catalytically inactive G9a enzyme. The de novo variants in EHMT2 lead to similar, but identifiably distinct, epi-signatures compared to Kleefstra Syndrome in cell lines. In addition a mouse model for one of the variants (Ehmt2+/del_1076-1079) demonstrates phenotypes associated with Kleefstra Syndrome. The authors state the mechanism likely differs from that of the haploinsufficiency of EHMT1, and suggest a dominant negative mechanism. Overall, the data in the manuscript strongly support the causality of the variants and the mechanisms proposed. However, which the "dominant negative" mechanism is plausible, the study lacks the in vitro experiments in live cells to formally address the relative role of heterodimers and homodimers.

1. The authors state "Overall, our observations indicate that mutations in EHMT1 and EHMT2 affect the same epigenetic module, albeit with specific functional differences." Methylation patterns of EHMT2 variants resemble episignatures of EHMT1 related Kleestra Syndrome, measured by a MVP score. However, there was some uniqueness in the episignatures, highlighted by differences in histone modifications compared to Kleefstra Syndrome samples included in the study as well as published studies. Despite differences in histone modifications, changes in gene expression and DAVID enrichments were similar across P2 and KSP fibroblasts and P1, P2, and KSP iPSCs. The authors highlighted shared differentially expressed genes and enrichment terms but did not do any DAVID enrichment/GO term enrichment for differentially expressed genes not shared between EHMT2 and EHMT1 mutations. This may speak to further functional differences between the mutations and the pathogenic mechanisms.

We thank the reviewer for this thoughtful comment. To address this point, we performed additional pathway enrichment analyses focusing specifically on genes upregulated exclusively in EHMT2-mutant cell lines. This analysis revealed that EHMT2 mutant cell-lines exhibited a coordinated stress-adaptation signature characterized by activated ER stress and unfolded protein response, accompanied by autophagy and altered cholesterol biosynthesis in fibroblasts, and upregulated oxidative phosphorylation and aerobic respiration in iPSCs.

We have added this observation in the Results section, lines 249-255, in the Discussion lines 473-477 and we present these data in revised Figure 3D and E.

Overall, comparative analysis of genes upregulated in EHMT1- and EHMT2-mutant cell lines revealed largely overlapping transcriptional programs, consistent with shared roles in repressing gene expression. However, consistent with the presence of an EHMT2-specific episignature, we identified gene sets and functional enrichments that were selectively upregulated in EHMT2 mutant cell lines. This point is described in Results section, lines 255-258 of the revised manuscript.

2. The authors describe a new de novo mutation in EHMT2 discovered by having the same episignature (Patient 7). However, they rule the variant likely benign due to the variant being outside of the methyltransferase domain and having normal methyltransferase activity and protein stability. The authors do not discuss potential reasons why the patient has a matching episignature and clinical phenotypes similar to Kleefstra Syndrome. I think this highlights a weakness in the idea that this episignature is unique to EHMT2 variants that genocopy Kleefstra Syndrome and could be used for identifying candidates. The manuscript would benefit from further discussion or investigation of other variants the patient may have that is driving the episignature.

We thank the reviewer for this comment, which shows that we did not sufficiently emphasize the difference between the pathogenic variant in patient P7 (A1077T) and the likely benign variant (T961I) previously reported in the literature in a patient with autism.

Patient P7 (A1077T) was added to the study during preparation of the original manuscript based on a matching Kleefstra syndrome 1 episignature, genotype and phenotypic overlap. Patient P7's DNA methylation profile served as a test sample for the EHMT2-specific episignature established using patients P1-P6 (see in lines 213-219).

In response to reviewers' comment, we performed biochemical experiments to assess the enzymatic activity, ligand binding capacity and structural stability of the A1077T variant. These analyses showed significantly unaffected structural stability but decreased methylation activity due to reduced histone H3 tail binding, consistent with the behavior of other pathogenic EHMT2 variants identified in our study. These results have been added to the Results section (lines 293, 297 and 325) and are presented in the revised Figure 5.

The heterozygous G9a variant p.T961I was previously reported as a variant of unknown significance in a patient with an autistic disorder, with no phenotypic overlap to Kleefstra syndrome 1 with unknown episignature profile. Biochemical behaviour of this variant differs from that of the pathogenic variants identified in this study. Therefore, we classify this variant as likely benign. To clarify our findings this point and emphasize the distinct behavior of the T961I variant, we have revised the Results section lines 346-351.

In summary, variant A1077T was found in a patient with clinical overlap with Kleefstra syndrome 1 and demonstrated to be pathogenic, while variant T961I was described in a patient with no phenotypic overlap with Kleefstra syndrome 1 and it is likely benign.

3. The manuscript suggests that the pathogenic mechanism of EHMT2 variants differs from the haploinsufficiency observed in EHMT1 variants. However, this distinction remains unclear. Given that EHMT2 variants still produce a stable G9a protein capable of forming heterodimers with GLP, one could argue that these variants may deplete the pool of GLP proteins available for forming functional heterodimers. This scenario could mimic the effects of EHMT1 haploinsufficiency, where a reduction in functional heterodimers contributes to disease pathology. A major limitation of this study is all analysis of the GLP/G9a interaction is performed using purified protein, rather than an in vitro cellular system. Thus, if the authors propose that EHMT1 homodimers compensate for the loss or reduction of functional heterodimers, it would strengthen the argument to demonstrate an increase in EHMT1 homodimer formation in patient fibroblasts or other cell line.

We acknowledge the reviewers remark noting that data from *in vitro* cellular system would strengthen our suggestions about the pathogenetic mechanism of EHMT2 variants. To address this point, we performed additional experiments as follows:

Firstly, we assessed the levels of *EHMT1/2* transcripts and GLP/G9a protein in blood and patient-derived cell lines. The transcript levels of *EHMT1/2* were not systematically changed, despite minor alterations observed in fibroblasts and iPSCs. The amount of GLP and G9a protein levels were also similar in controls and patient-derived samples. These data have been added to the Resullts Section lines 265-270 and Supplementary Figure 9B and C.

Secondly, we performed immunoprecipitation of G9a from iPSCs, followed by western blotting and mass spectrometry, to assess its binding to GLP. We observed that GLP, alongside with other known G9a interactors such WIZ and ZNF462, was efficiently co-immunoprecipitated in both control and patient cell lines. Together, these additional experiments provide further evidence that the identified EHMT2 variants do not impair the formation of the GLP–G9a heterodimers, strongly supporting their dominant-negative effect. We have added these results to the Results section (lines 270-276), Supplementary Figure 9 and Table S3 and Methods (lines 170-201).

We would like to comment on the reviewer's statement *"authors propose that EHMT1 homodimers compensate for the loss or reduction of functional heterodimers, it would strengthen the argument to demonstrate an increase in EHMT1 homodimer formation in patient fibroblasts or other cell line."*
We would like to clarify that this statement refers to scenario involving reduced amount of G9a protein (e.g., in $Ehmt2^{+/-}$ mice) and is therefore not applicable to the identified EHMT2 missense variants. We have revised the corresponding paragraph accordingly (Discussion, lines 553-559).

Overall, the data presented in the current manuscript strongly suggests that these variants act in dominant-negative manner. A plausible mechanism is that the inactive mutant complexes occupy chromatin target sites thereby competing with other functional complexes for

chromatin binding. This contrasts with the scenario of EHMT1 haploinsufficiency in which the level of assembled heterodimers is reduced. We have added these ideas to the Discussion section (lines 555-559).

Additionally, if GLP-G9a heterodimers are preferentially formed and have higher catalytic activity compared to G9a homodimers, this could potentially explain the milder phenotypes observed in G9a+/− mice relative to the Ehmt2+/del_1076-1079 variant, where the GLP pool is not depleted by non-functional heterodimers. Clarifying this point with supporting biochemical or functional data would strengthen the mechanistic interpretation.

We thank the reviewer for this insightful comment. In response, we performed additional experiments to evaluate the effects of the variants Del1076–1079 and A1077S on GLP–G9a heterodimerization and enzymatic activity. We adapted our experimental design according to previously published work that demonstrated preferential formation of GLP-G9a heterodimers and its enhanced activity on nucleosomes (Sanchez, N.A et al. J. Biol Chem 297, 101276 (2021)). These effects were attributed to regions comprising the ankyrin repeats and the methyltransferase domain. Accordingly, we co-expressed the corresponding GLP and G9a constructs containing both the ankyrin and the methyltransferase domains, purified the resulting complexes and analyzed them using our established laboratory protocols. Native mass spectrometry revealed the predominant formation of heterodimeric complexes for all variants tested. Methylation activity assays showed only a modest reduction with histone H3 peptide (to ≈ 70 % of wild-type), whereas activity with nucleosomes was markedly diminished (to less than 35 % of wild-type). Importantly, nucleosomal methylation activity was reduced below 50% of wild-type levels for both mutant variants, suggesting that heterodimer-associated GLP cannot compensate for catalytically impaired G9a mutants.

Overall, the preserved G9a-GLP heterodimerization together with reduced nucleosome methylation further supports our proposed mechanistic interpretation of EHMT2 mutations. These data have been added to the Results sections, lines 304-314, to Figure 5A (left panel), Supplementary Figure 12 and Methods section lines 212-215 and 256-257.

4. The observation that approximately 50% of the mice in this Ehmt2+/del_1076-1079 model died between P30–40 is interesting. However, all phenotyping analyses were conducted on animals that survived beyond this timepoint. To more comprehensively characterize the model and similarities to Kleefstra Syndrome, I would recommend including assessments of mice during prior to the P30-40 timepoint. This could help uncover early-onset phenotypes and provide insight into potential causes of lethality. Specifically, evaluating cardiac anomalies or more pronounced craniofacial abnormalities may reveal underlying defects contributing to early mortality. The potential identification of cardiac anomalies would also strengthen the model for Kleefstra Syndrome.

We thank the reviewer for this important and insightful comment. We agree that analysis of mice prior to the P30–40 timepoint could provide valuable information regarding early-onset phenotypes and potential causes of lethality.

It should be noted that Ehmt2+/del_1076-1079 animals failed to reproduce. Thus, all the mice presented in the studies had to be generated by *in vitro* fertilization, which represents a significant logistical and experimental challenge and substantially limits breeding capacity and animal numbers. To respond and prepare the revised version of the manuscript, we reviewed the data that we generated prior the original submission within the complex phenotyping pipeline of the Czech Centre for Phenogenomics.

The majority of male and female pups died earlier than p10 and p20, respectively. As a result, a substantial fraction of these non-surviving animals was eaten-up by the mothers prior being even noticed by the care-taking personnel. In total, we were able to retrieve the carcass and/or perform an urgent autopsy in 7 moribund animals. These seven pups/carcasses were gross anatomically and histologically evaluated and no cardiac developmental defects were identified.

To reflect the reviewer´s comment, we incorporated a note about the post-mortem findings in the animals that we were able to retrieve either as a carcass or via an urgent necropsy. In addition, we incorporated morphological and functional echocardiographic data in surviving mice at 4 months of age. Changes were made in the Results (lines 388-390) and in the Methods. Four additional Supplementary Figures 26-29 were compiled showing minimal anatomic or functional cardiac abnormalities.

Cardiac developmental defects are frequent in Kleefstra syndrome 1 patients (Xie et al, 2025), however, their nature is heterogeneous and ranges from ventricular and atrial (septal) formation defects, valvular and large vessel(s) anomalies to cardiomyopathy and/or coronary artery abnormalities. We report a similarly heterogeneous spectrum of cardiac issues in our patients with *de novo* variants in EHMT2. However, cardiac abnormalities have not been consistently reported in Ehmt1 haploinsufficient mouse models, which parallels our observations in the Ehmt2 mice model. Therefore, the absence of apparent cardiac defects in the examined Ehmt2 mutant animals does not necessarily argue against the relevance of the model for Kleefstra-related disease mechanisms.

We agree that elucidating the causes of early-postnatal mortality is an important goal. However, to obtain statistically meaningful data for early mortality (50% loss), a study of similar size to the one presented in the originally submitted manuscript would be needed, and prenatal and/or early postnatal imaging methodologies would need to be implemented. We believe that such analyses are beyond the scope of the current manuscript, but represent an important direction for future work.

Please, see also our responses to Reviewer 3, who raised similar comments.

Typo: In the body of the manuscript the variant a mouse model was generated for was reported to be the Ehmt2+/del_1076-1079 (line 323), but thereafter it is referred to as

Ehmt2+/del_1076-1076 (line 329, 331 etc).

We thank the reviewer for noticing this error. We corrected all the incorrect variants.

We thank the reviewer for the suggestions. We hope that the additional experiments and corrections address the reviewer's concerns and that the revised manuscript is significantly improved.

Reviewer #2 (Remarks to the Author):

I co-reviewed this manuscript with one of the reviewers who provided the listed reports. This is part of the Nature Communications initiative to facilitate training in peer review and to provide appropriate recognition for Early Career Researchers who co-review manuscripts.

Reviewer #3 (Remarks to the Author):

Key results:
This paper describes novel mutations in the EHMT2 substrate binding site in patients exhibiting similar symptoms to Kleefstra syndrome. The authors show that the patients have a specific episignature, distinct from Kleefstra, and other similar syndromes. The authors provide a number of analyses to show that these mutations lead to functional decreases in enzymatic activity, and provide further data to show that these are either due to a lack or reduction of binding to H3, or SAM.
They generated a new mouse model to demonstrate that one of these mutations is sufficient to recapitulate some physical and developmental problems observed in the patient, something that has not been observed previously with heterozygous loss of Ehmt2. Based on these findings, they propose a plausible model suggesting that these mutations function in a dominant negative manner to reduce the activity of the EHMT1/2 heterodimeric complex on chromatin.
Validity:
For the most part the data interpretation and conclusions drawn are sensible in light of the data presented and references drawn upon. There are a couple of overreaches in interpretation, which I have highlighted in the suggested improvements below.
Significance:
This paper makes an important contribution to the field by providing the first evidence of an EHMT2 syndrome caused by potential dominant negative mutations in EHMT2. Some minor edits would increase clarity as suggested below.
Data and methodology:
Changes to histone methylation were associated with changes in gene expression in cell lines generated from patients. Appropriate healthy controls and KSP controls were used. Multiple different functional assays were used to convincingly demonstrate a change in enzyme activity caused by the novel mutations, including direct measures of changes to histone

methylation. The use of the animal model to demonstrate Ehmt2 mutations as causative of features of the syndrome was appropriate and added strength to the conclusions, however, it was underutilised and demonstration of some key developmental anomalies seen in patients would significantly strengthen their conclusions. I have highlighted in the suggested improvements where I feel the data presented could be improved to increase clarity or confidence in the data presented.

Analytical approach

There is no mention of numbers of experimental or technical replicates for many of the experiments. The numbers of replicates, sex and statistical tests applied should be stated explicitly in the figure legends. No statistical test appears to have been applied to the assay measuring methyltransferase activity – likely due to the high number of assays which had no detectable activity. Number of replicates was also not reported for data reported in this figure (Fig 5), making it hard to evaluate how robust this data is. This should be reported, and ideally individual replicates should be plotted as datapoints on the graph to increase transparency. The animal data presented is more robust, with a sufficient number of replicates (as made visible by individual data points being plotted), and proper attention paid to potential sex differences. However, number of animals used per group is not reported in the main text or the figure legend and should be visible in at least one of these places. Methods should explicitly state whether researchers were blind to genotype, especially for measuring craniofacial features reported in Fig. 6D.

Clarity and context:

The manuscript is clearly written with good inclusion of previous work to understand context.

References:

The authors have used appropriate references. The addition of a reference should be included to support the speculation on line 222 that the genes regulated in the variants are direct targets of G9a if one is available. If not, this statement should be supported by experimental data.

Three different references that support that upregulated genes are the most likely G9a targets have been added to the manuscript.

Suggested improvements:

1. The numbers of replicates, sex and statistical tests applied should be stated explicitly in all figure legends.

Based on these remarks, we have added information on the number of replicates and the statistical tests used to the corresponding figure legends throughout the manuscript. Moreover, we modified the bar plots to display individual data points. In the revised version of manuscript, the animal data are mostly presented as dot plots so that the numbers of animals of each genotype and sex are easily identifiable. In cases in which dots are not displayed (Figure 6A and 6B), animal numbers are indicated in the figure or its legend. The only exception are the weight data in Figure 6B. Not to make the Figure 6 and Figure 6

legend overloaded with data, we added animal numbers for each time-point/genotype/sex to the Supplementary Table S8.

2. Fig. 3C is too high level to be sufficiently informative. I suggest replacing it with a more informative plot e.g. Suppl. Fig 6A, B.

We thank the reviewer for this suggestion. Figure 3C is a correlation matrix that was included to facilitate comparison of gene expression changes across multiple cell lines, and it is a common representation for assessing global similarity between conditions. In response to the reviewers point, we have improved its interpretability, by adding the Pearson correlation values directly to the matrix and specifying “Pearson correlation” in the heatmap color legend. Detailed gene-by-gene correlation plots can be found in Supplementary Figure 6A and B, which allow inspection of the underlying relationships at higher resolution. We hope that the revisions made to Figure 3C improve its clarity and facilitate interpretation.

3. From my understanding of the design of the dimerization assays used, these were measures of homodimer formation – is data available for rate of heterodimer formation? I ask because as the authors state in the introduction, heterodimers are the preferred form in vivo with maximal activity. Ratio of hetero:homo dimer formation with the variant compared to a control may also be biologically interesting if the data is available (refers to Fig 5B and Supp Fig. 12).

We thank the reviewer for arising this important point. Based on this remark, we assessed the effects of EHMT2 variants on heterodimer formation and activity in additional experiments.

We examined GLP–G9a heterodimerization of wild-type G9a and mutant variants Del1076–1079 and A1077S based on a previously reported work (Sanchez, N.A et al. J. Biol Chem 297, 101276 (2021)). This study demonstrated the preferential formation of GLP-G9a heterodimer and its enhanced activity on nucleosomes is maintained by GLP and G9a proteins comprising the ankyrin repeat region and methyltransferase domain. Accordingly, we co-expressed the corresponding GLP and G9a proteins, purified resulting complexes and analyzed their composition using native mass spectrometry. These analyses revealed predominant formation of heterodimeric complexes for all variants tested. Moreover, mutant heterodimers showed reduced activity with nucleosomes. These data have been added to Results, lines 304-314, Figure 5A and to Supplementary Figure 12B.

Furthermore, the formation of heterodimers was validated in-vivo by immunoprecipitation of G9a from patient-derived and control iPSCs, followed by western blotting and mass spectrometry. We observed that GLP, alongside with other known G9a interactors such WIZ and ZNF462, was efficiently co-immunoprecipitated in both control and patient-derived cell lines. These results have been added to Results (lines 270-276), Supplementary Figure 9 and Table S3.

Together, these data demonstrate that GLP-G9a heterodimer formation is favored for both wild-type and mutant EHMT2 variants. This conclusion has been added to the Results (lines 320-322).

4. Methods should explicitly state whether researchers were blind to genotype, especially for measuring craniofacial features reported in Fig. 6D.

Yes, the researchers were blind to the genotype of the mice when performing all the tests. A note has been added to Methods and to legend to Figure 6.

5. To validate the mouse model the authors assess the mice by SHIRPA, show reduced activity and cranioskeletal changes. These are insufficient to draw the conclusion that the mice represent a good model for EHMT2 Kleefstra syndrome. Can the authors assess and report the incidence of a couple of other key features such as the incidence of cardiac and kidney/urogenital anomalies characteristic of the condition? These are relatively straight-forward to assess and score and will substantially increase confidence in this conclusion.

The murine study represents one piece of data in the mosaic of the evidence presented in the manuscript that jointly document the deleterious effects of de novo EHMT2 pathogenic variants.

Indirectly supporting our gain-of-function hypothesis, heterozygous Emht2 KO mice (Ehmt2$^{+/-}$, Iacono et al 2018) presented with a minimal or no phenotype. Contrary to Ehmt2$^{+/-}$, Ehmt1$^{+/-}$mice show a phenotype that is considered relevant to EHMT1 Kleefstra syndrome 1.

As is outlined in the manuscript, we designed the mouse model (Ehmt2$^{+/del_1076-1079}$) to carry an Ehmt2 variant equivalent to the one identified in Patient 1 (P1). The key reasons for such a strategy were the absence or minimal phenotype in Ehmt2$^{+/-}$ mice and to test a phenocopy of the human variant at the organismal level. Characterization of our model was carried to mirror the published studies in the Ehmt1$^{+/-}$ animals. Similar to these papers by Balemans et al, we focused on phenotypic traits in the mice systematically present in Kleefstra syndrome 1 patients. The presented results are the initial characterization of the model. We definitely plan to utilize the model for further biochemical, cellular and tissue analyses, which, nonetheless, are, in our opinion, beyond the scope of this manuscript.

Cardiac developmental defects are frequent in Kleefstra syndrome 1 patients (e.g. Xie et al 2025), however, their nature is heterogenous and ranges from ventricular and atrial (septal) formation defects, valvular and large vessel(s) anomalies to cardiomyopathy and/or coronary artery abnormalities. We report a similarly heterogenous spectrum of cardiac issues in our patients with *de novo* variants in EHMT2. Urogenital defects do not systematically appear in our patient cohort (see the clinical data Table 1).

It should be noted that Ehmt2$^{+/del_1076-1079}$ animals failed to reproduce. As a result, all the mice presented in the studies were generated by *in vitro* fertilization. To respond and prepare the revised version of the manuscript, we reviewed the data that we generated prior the original

submission within the complex phenotyping pipeline of the Czech Centre for Phenogenomics.

Majority of male and female pups died earlier than p10 and p20, respectively. As a result, a substantial fraction of these non-surviving animals was eaten-up by the mothers prior being even noticed by the care-taking personnel. In total, we were able to retrieve the carcass and/or perform an urgent autopsy in 7 moribund animals. These 7 pups/carcasses were gross anatomically and histologically evaluated and no cardiac developmental defects were identified.

To reflect the reviewer´s comment, we incorporated a note about the post-mortem findings in the animals that we were able to retrieve either as a carcass or via an urgent necropsy. In addition, we incorporated morphological and functional echocardiographic data in surviving mice at 4 months of age. Changes were made in the Results (lines 388-390 and 421-425) and in the Methods. Four additional Supplementary Figures 26-29 were compiled showing minimal anatomic or functional cardiac abnormalities.

To complement the neurological-developmental evaluation of the patients in the motor abilities domain, we added data on a subtle, yet, systematically present gait defect in the $Ehmt2^{+/del\_1076-1079}$ mice. Changes were introduced into the Results (lines 409-415), Methods, and a novel Supplementary Figure 23 was compiled. We also attached two illustrative videos (S1 and S2) comparing gait patterns in $Ehmt2^{+/del\_1076-1079}$ and $Ehmt2^{+/+}$ animals.

We consider this mouse model to be relevant for EHMT2-related disease. First, the model has strong genetic validity, since it reproduces the same in-frame deletion identified in patient 1. Second, the model shows developmental abnormalities, reduced viability, craniofacial abnormalities, growth delay, and neuro-motor phenotypes, supporting its relevance as a model for EHMT2-related neurodevelopmental disease. Therefore, similar to the $Ehmt1^{+/-}$ mouse model, our mouse model reproduced phenotypic traits systematically present in EHMT2 patients. Importantly, the severity of the phenotype supports a dominant-negative mechanism of action for the EHMT2 variants rather than haploinsufficiency, as the $Ehmt2^{+/-}$ mice display a very mild phenotype.

6. The description and interpretation of the DNAme analyses should be improved and discussed. The episignature data presented (e.g. in Fig. 2B) shows that EHMT2 syndrome samples group together, distinct from "classical" Kleefstra syndrome and controls. However, in the summary the authors state that episignatures are similar to Kleefstra syndrome. Can the authors clarify these interpretations and statements?

We agree that this section should be clarified. EHMT2-related disorder and Kleefstra syndrome 1 share overlapping genome-wide DNA methylation patterns, consistent with their shared related molecular functions and regulatory pathways. This supported the pathogenicity assessment of the tested EHMT2 variants.

Next, we were able to identify a distinct set of differentially methylated probes that forms a robust, condition-specific episignature for EHMT2. Using our EpiSign Discovery Pipeline, including support vector machine (SVM) training, this probe set provides a highly accurate, sensitive, and specific classifier that clearly differentiates EHMT2 from controls and Kleefstra syndrome 1 patients and patients with other disorders of known episignatures.

These observations are described in Results lines 210-222. To address reviewer's remarks, we have revised the paragraph in Discussion, lines 444-450.

Minor edits:
7. Supplementary figure 1 legend: at the end of this there appears to be a sentence copied from the supplementary figure 3 legend.

Thank you for pointing out the error, we corrected corresponding figure legends accordingly.

8. Inclusion of all the differential methylation data is appreciated but addition of a visual summary of supplementary table 1 would be appreciated for ease of interpretation.

As requested by the reviewer we have added a volcano plot showing mean methylation differences and p-values of all probes, with the episignature probes highlighted in red to Supplementary Figure 2A.

9. Inclusion of negative controls would increase confidence in the immunofluorescent staining shown in Supplementary figure 4.

Negative controls for the immunostaining have been added to Supplementary Figure 4. Supplementary Figure 4B shows that fibroblasts stain negative for iPSCs markers OCT4, SOX2, NANOG and TRA1.60. Supplementary Figure 4C shows that iPSCs stain negative for differentiation markers AFP, TUBIII and ASMA.

10. Line 208. Please reference the papers describing the established standard of iPSC culture generation & definition.

References for the standard characterization of iPSCs have been added to Results, line 232.

11. Fig 6B – please insert statistics.

Statistics for both sexes at all the presented times were already summarized in the legend to Figure 6B. As the differences are significant throughout the entire testing period, we did not insert the statistical annotations in the figure itself to maintain clarity. The additional information about data distribution and statistical analysis are included in Supplementary Figure 16, Supplementary Table S8, which are referenced in Legend to Figure 6.

12. Methods section for solubility of GLP requires revisions for typos.

We have thoroughly updated and revised the whole Methods section and corrected the typos.

13. Please make breakdown of SHIRPA test available as a supplementary table.

As requested by the reviewer, SHIRPA scores have been provided in the revised version of the manuscript in Supplementary Table S7. The Supplementary Figure 21 has been modified and contains cumulative locomotor activity data only (see Methods for further details).

14. Line 347-348: As this was not statistically significant this feels like an overinterpretation, especially as the difference between the Δ is not large. The labelling of supplementary fig 23 is not clear and there are typos in the legend.

We agree with the reviewer that the statement allows ambiguous interpretation. We therefore removed the sentence “suggesting their better ability to learn in this motor coordination task” from this paragraph. Additionally, we have corrected the typos in the legend.

15. Supplementary figure 25 legend: typos.

The typos in Supplementary Figure 25 legend have been corrected.

Expertise:
Mouse models of chromatin disorders.

Reviewer #4 (Remarks to the Author):

I co-reviewed this manuscript with one of the reviewers who provided the listed reports. This is part of the Nature Communications initiative to facilitate training in peer review and to provide appropriate recognition for Early Career Researchers who co-review manuscripts.

Reviewer #5 (Remarks to the Author):

The authors are to be congratulated on an important report of a novel disease causing gene and its associated phenotype. The authors provide clear functional evidence for the reported variants in EHMT2. Functional studies are convincing and well explained. The patients are described in appropriate detail in the table and the provided photographs.
Particularly interesting is the distinctive episignature, which identified an additional patient (P7).

We appreciate positive feedback from the reviewer.

Specific comments:
Why is the 7th patient not counted in the total number of cases in the abstract? It should be included.

Patient P7 had been added to the study during the finalization of the original manuscript based on a genotype, phenotypic overlap and matching Kleefstra syndrome 1 episignature. The P7 served as a test sample for EpiSign Discovery Pipeline for EHMT2-specific signatures. During the revision of the manuscript, we performed the biochemical characterization of the variant identified in this patient. These results have been added to Results lines 293, 297 and 325 and to Figure 5). As suggested by the reviewer, the patient P7 has been included in fully characterized patient cohort.

In the title and throughout the manuscript the term "genocopy" is used. While the term "phenocopy" is used for a condition that looks just like another condition, a similar term "genocopy" is not commonly used.

Thank you for raising this issue. We used the term “genocopy” because it accurately describes that the new condition caused by variants in EHMT2 results in phenotypic features consistent with Kleefstra syndrome 1. We agree with the reviewer that this term is not commonly used, so to avoid confusion we have changed the title to: “*De novo EHMT2* variants **cause an autosomal dominant *EHMT2*-related Kleefstra syndrome** via loss of G9a methyltransferase activity”.

The authors should decide what they consider this newly reported condition. Is this EHMT2-related neurodevelopmental disorder? Is it EHMT2-related Kleefstra syndrome? Is it EHMT2-related Kleefstra syndrome type 2? Once this decision is made, the manuscript should be carefully edited for consistency. It is at times unclear when the term Kleefstra syndrome is used if this refers to EHMT1-related Kleefstra syndrome or the novel condition or a combination of these. For example in the figure showing the effects of the variants in the different genes, both are referred to as "Kleefstra syndrome". In summary, the messaging regarding the phenotype and its naming needs to be tightened and explained, then applied to the entire manuscript.

We thank the reviewer for bringing this important issue to our attention. The nomenclature of Kleefstra syndrome in OMIM is indeed confusing. OMIM currently lists two entries related to Kleefstra syndrome. Kleefstra syndrome 1 (KLEFS1, #610253) refers to conditions caused by subtelomeric deletions of chromosome 9q that include the EHMT1 gene, as well as pathogenic variants within EHMT1 itself. The second entry, Kleefstra syndrome 2 (KLEFS2, #617768), is associated with de novo heterozygous mutations in the KMT2C gene. However, pathogenic variants in KMT2C lead to a neurodevelopmental disorder that is clinically distinct from Kleefstra syndrome 1. Although Prof. Kleefstra has argued against this naming convention in OMIM, the terminology remains in use. Therefore, we choose not to use the

term Kleefstra syndrome 2 to refer to patients with EHMT2 mutations since it might be confused with mutations in KMT2C.

Taking into account this information and following the reviewer’s advice, we have adopted a consistent nomenclature for the syndromes in the manuscript. We have used the term Kleefstra syndrome 1 when referring to patients with EHMT1 alterations. For patients with alterations in EHMT2, we have used the term EHMT2-related syndrome/disorder through the manuscript. In the Title, Abstract and Discussion lines 565-567, we propose to call this newly identified syndrome autosomal dominant EHMT2-related Kleefstra syndrome, given the phenotypic and molecular similarities with Kleefstra syndrome. We are also aware that autosomal recessive “AR EHMT2-related” Kleefstra syndrome is under active investigation.

We modified the naming throughout the manuscript referring to Kleefstra syndrome 1 when mentioning patients with pathogenic variants in EHMT1. We also included proposed nomenclature to Figure 7 that should clearly distinguish between Kleefstra syndrome 1 and AD EHMT2-related Kleefstra syndrome.

For clinicians, the facial features are important in order to identify the patients. The authors did well in showing several patients. If additional photographs of the 7th patient can be shown, please include these.

We re-contacted the patients' families and the family of P7 agreed to share the photographs. We included these in Figure 1.

The usefulness of the episignature analysis, potentially in combination with genome or exome analysis, should be stressed (even more). This newer tool is underutilized and should become a standard in the evaluation of undiagnosed patients with neurodevelopmental disorders.

We agree with this remark. Episignature analysis—especially when paired with genome or exome sequencing—is still underused, but not yet available in routine genetic testing. Following the advice of the reviewer, we added the following sentence to the Discussion (lines 455-456) “These findings further highlight the value and clinical relevance of episignature analysis in assessing variants of uncertain significance.”

We thank this reviewer for the constructive suggestions. We hope that the revisions and corrections introduced in the revised version of the manuscript satisfactorily address the reviewer’s comments.
